# Tracing the introduction of the invasive common myna using population genomics

**DOI:** 10.1038/s41437-023-00621-w

**Published:** 2023-05-17

**Authors:** Kamolphat Atsawawaranunt, Kyle M. Ewart, Richard E. Major, Rebecca N. Johnson, Anna W. Santure, Annabel Whibley

**Affiliations:** 1 School of Biological Sciences, University of Auckland, Auckland, New Zealand; 2 Australian Museum Research Institute, Australian Museum, Sydney, NSW Australia; 3 School of Life and Environmental Sciences, University of Sydney, Sydney, NSW Australia; 4 National Museum of Natural History, Smithsonian Institution, Washington D.C., DC USA

**Keywords:** Genetic variation, Evolutionary biology

## Abstract

The common myna (*Acridotheres tristis*) is one of the most invasive bird species in the world, yet its colonisation history is only partly understood. We identified the introduction history and population structure, and quantified the genetic diversity of myna populations from the native range in India and introduced populations in New Zealand, Australia, Fiji, Hawaii, and South Africa, based on thousands of single nucleotide polymorphism markers in 814 individuals. We were able to identify the source population of mynas in several invasive locations: mynas from Fiji and Melbourne, Australia, were likely founded by individuals from a subpopulation in Maharashtra, India, while mynas in Hawaii and South Africa were likely independently founded by individuals from other localities in India. Our findings suggest that New Zealand mynas were founded by individuals from Melbourne, which, in turn, were founded by individuals from Maharashtra. We identified two genetic clusters among New Zealand mynas, divided by New Zealand’s North Island’s axial mountain ranges, confirming previous observations that mountains and thick forests may form barriers to myna dispersal. Our study provides a foundation for other population and invasion genomic studies and provides useful information for the management of this invasive species.

## Introduction

Invasive species are organisms which are not native to a particular area that may reproduce and expand demographically and spatially (Pyšek and Richardson 2010), and potentially cause economic or environmental harm or harm to human health (Matheson and McGaughran 2022). In the last 50 years, the financial cost of invasive species has been estimated to exceed $USD 1.288 trillion globally (1970–2020; Zenni et al. 2021). Invasive species are one of the leading causes of biodiversity loss and ecosystem degradation, with considerable impacts on native species via predation and competition (Clavero and García-Berthou 2005; Pyšek and Richardson 2010; Simberloff et al. 2013; Luque et al. 2014). The impacts of invasive species are a growing concern as increases in global connectivity, habitat modification and climate change have led to an increase in the number and distribution of invasive species in recent decades (Hulme 2009; Turbelin et al. 2017).

Information on invasive species, such as their origin, dispersal characteristics, and population dynamics informs their management and can help predict risk of invasion into novel areas (Rollins et al. 2009; Cassey et al. 2014; Evans et al. 2018; Fournier et al. 2019). Population genetic methods can be used to provide information on invasion history, population connectivity and demographics (Rollins et al. 2006; Marrs et al. 2008; Kekkonen et al. 2011), and can provide insight into the existing or novel adaptations that have enabled invasion success (Prentis et al. 2008).

Common mynas (*Acridotheres tristis*, hereafter mynas) have established from their native range in Central to Southeast Asia to become a globally invasive species and are one of only three bird species on the IUCN ‘100 of the World’s Worst Invasive Alien Species’ list (Global Invasive Species Database 2021). In the 18th century, the French deliberately introduced Indian mynas to Mauritius and Reunion Island to control insect pests (Cheke and Hume 2008), making this one of the world’s first attempts at biological pest control (Safford and Hawkins 2013). Mynas were subsequently intentionally introduced to at least 13 locations across the world, but have also successfully self-introduced globally (Long 1981). Mynas are now invasive in Africa, North America, Europe, the Middle East, Australia, and numerous island nations including Madagascar, Fiji, Maldives, and New Zealand (Feare and Craig 1999; Peacock et al. 2007; Hart et al. 2020; CAB International 2021).

Mynas have a generalist, omnivorous diet and can themselves be agricultural pests (Dawson and Bull 1970; Hone 1978; Peacock et al. 2007) and can have negative impacts on native ecosystems, especially island ecosystems with high level of endemism (Hart et al. 2020). These impacts include direct competition with native birds and mammals for food and nests, including taking over native birds’ nest cavities, predation on eggs and chicks (Hart et al. 2020), and assisting with the dispersal of invasive and exotic plants (Pimentel et al. 2000; Parkes 2006; Saavedra 2009). In addition, mynas may also impact human health and livelihood through the spread of disease, noise pollution, and property damage (Yap et al. 2002; Parkes 2006; Saavedra 2009; Clark et al. 2015). Eradication of mynas on islands has been found to positively impact the native fauna, for example, the increase in numbers of the native Seychelles magpie robins and Seychelles paradise flycatchers accelerated after removal of 90% of mynas from Denis Island, Seychelles (Feare et al. 2021).

Mynas are thought to have been introduced to New Zealand from Australia in the 1870s (Thomson 1922; Long 1981; CAB International 2021), where they had been introduced from the native range, likely India, in the 1860s (McCoy et al. 1885; Ewart et al. 2019). However, a review of historical data and literature by Beesley et al. (2023) on myna introduction history and distribution in New Zealand suggests some uncertainty in the timing, the number of individuals, the number of introductions, and their source. For instance, there is some evidence that the first introduction of mynas in New Zealand may be earlier than the 1870s (e.g. Huddleston 1868), and it is unclear which of the initial introductions to New Zealand survived and formed the current populations. Since their initial introduction into New Zealand, mynas have been translocated/introduced around the country (e.g. to Whanganui, New Plymouth, Hawke’s Bay), but their exact colonisation history remains unclear. In addition, mynas are no longer seen at many of their initial introduction points into New Zealand (e.g. Nelson, Christchurch, Dunedin, and Wellington).

Population genetic data can help elucidate the colonisation history, and by extension invasion pathways, and identify gene flow between populations. Restriction-site associated DNA sequencing (RADseq) and similar genotyping by sequencing (GBS) methods subsample the genome by using restriction enzymes to fragment genomic DNA and then sequence adjacent to the cut sites with next-generation sequencing (Baird et al. 2008; Wright et al. 2019). This generates genome-wide high-throughput sequencing data, making it a suitable genotyping method for obtaining individual-level genotype information that can be compared across individuals and populations. Consequently, RADseq has been used in numerous ecological, evolutionary, and conservation genomic studies to address varied questions (Andrews et al. 2016), such as describing population structure and connectivity, detecting hybrids, resolving phylogenies, and identifying genome regions under selection (Near et al. 2018; Ewart et al. 2019; Hofmeister et al. 2021; Stuart et al. 2021; Forsdick et al. 2021). Although RADseq does not require a reference genome (Baird et al. 2008; Peterson et al. 2012), the use of a reference genome typically improves the reliability of genotype calls and downstream inferences (Shafer et al. 2017).

Here, we utilise single nucleotide polymorphism (SNP) markers – identified from RADseq reads mapped against a draft myna genome assembly - from 814 myna individuals from six countries to 1) identify the likely sources of invasive population(s), and more specifically of the New Zealand myna population, 2) clarify our current understanding of the myna’s colonisation history in New Zealand, and 3) identify population structure and genetic diversity in New Zealand mynas. This study provides a foundation for further invasion genomic studies and useful insights for species management.

## Materials and methods

Data filtering and analyses performed in R were conducted in R version 4.1.2 (R Core Team 2021), with figures plotted using the ‘ggplot2’ R package version 3.3.6 (Wickham 2016) unless noted. See Table S2.1 for a summary of the software and packages used.

### Sampling, DNA extraction, sequencing and processing

**Fig. 1:**
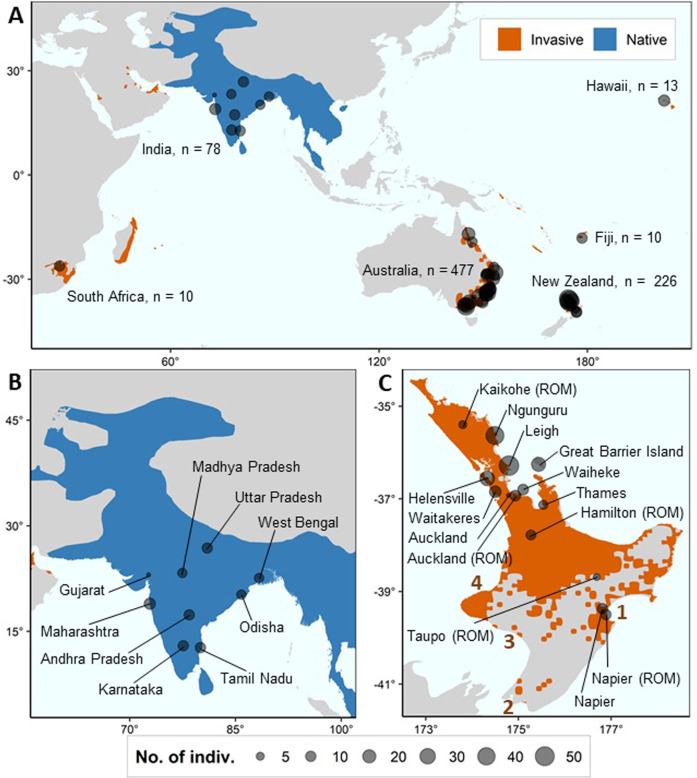
Map of locations of individuals sampled overlayed on top of the range distribution (BirdLife International and Handbook of the Birds of the World 2016). Blue represents the native range and orange represents the invasive range. Note that this figure does not cover the entire invasive range of the myna. All samples from India, Fiji, South Africa, and Hawaii were provided by the Royal Ontario Museum (ROM). Some samples from Australia and New Zealand were also provided by the ROM. Map of sample locations include: **A** entire dataset, **B** India, and **C** New Zealand. Within New Zealand’s North island (**C**), early introduction points pre-1980 are marked in bold brown numbers, namely, (1) Hawke’s Bay (Napier), (2) Wellington, (3), Whanganui, and (4) New Plymouth.

A total of 183 myna tissue samples in ethanol from India, New Zealand, Australia, South Africa, Hawaii and Fiji between 1975–1989 (Baker and Moeed 1980,1987; Fleischer et al. 1991) were received from the Royal Ontario Museum (ROM). A further 193 euthanized mynas were obtained from myna control programmes from contributors in New Zealand between 2017–2020, and muscle tissue was subsampled from each individual. DNA was extracted from the ROM tissue samples using the DNeasy Blood & Tissue Kit (Qiagen) following the manufacturer’s protocols. DNA was extracted from the New Zealand tissue samples using the Monarch Genomic DNA Purification Kit (NEB) following the manufacturer’s protocols. DNA concentration was measured using a Qubit 2.0 Fluorometer (Thermo Fisher Scientific). DNA was diluted to standardised concentrations of 50–100 ng/μL, and sent to Diversity Arrays Technology Pty Ltd company (DArT P/L) for further processing (Kilian et al. 2012; Cruz et al. 2013). Samples from 363 individuals were successfully sequenced using the proprietary Diversity Arrays Technology platform and protocol (DArTseq™). We included 13 duplicate samples. DArTseq also includes internal replicates of samples as part of its protocol. DArTseq is a restriction enzyme‐based genome complexity reduction method that has been used to generate SNP data for a range of studies (Melville et al. 2017; Ketema et al. 2020) including a previous population genomic study on Australian mynas (Ewart et al. 2019). We utilised the *PstI*-*SphI* restriction enzyme double digest following Ewart et al. (2019), enabling our samples to be processed and co-analysed with the DArTseq data from 451 mynas from Australia from the aforementioned study (mynas sampled in 2014–2015). In total, our study dataset comprised 814 mynas sampled from six countries (Fig. 1, Tables S1.1 and S1.2).

### Variant calling

Barcodes in DArTseq raw read sequences were removed using the *process_radtags* program in STACKS version 2.58 (Catchen et al. 2013) and adaptors trimmed using fastp version 0.20.0 (Chen et al. 2018). Quality control checks were performed using fastqc version 0.11.9 (Andrews 2010) and multiqc version 1.9 (Ewels et al. 2016). Reads were then aligned to a draft common myna reference genome generated from an Australian individual (see step 3 in Appendix S2 for more details) using BWA version 0.7.17 (Li and Durbin 2009). Reads were subsequently sorted and indexed using SAMTOOLS version 1.12 (Li et al. 2009). As detailed in Appendix S2.1, SNPs were called through the *mpileup* and *call* commands in BCFtools version 1.13 (Li 2011) based on three subsets of individuals – the entire dataset (ALL dataset: 814 individuals; 1024 samples including replicates), a New Zealand-only dataset (NZ dataset: 226 individuals, 282 samples) and an India-only dataset (IND dataset: 78 individuals, 80 samples). All analyses described below were performed on the genotypes called by BCFtools, but to ensure results were consistent, population structure analyses were also performed on SNP datasets called from other pipelines (STACKS and DArT P/L DArTsoft14 SNP calling algorithm). Population structure was congruent across the three SNP datasets (see Fig. S2.1 and Appendix S10 for more details).

### Data filtering

As described below, the BCFtools SNP datasets were filtered based on locus data quality (e.g. SNP quality score, read depths, genotype quality scores), sample data missingness, locus data missingness, and linkage disequilibrium, and finally filtered to only retain SNPs that are under Hardy-Weinberg equilibrium (HWE). Depending on the analyses performed, the datasets were filtered using different stringencies (see Fig. S2.2 for more details). Variants were output in binary variant call format (BCF) and were converted to variant call format (VCF) and compressed VCF (vcf.gz) using BCFtools for uses in different software.

#### SNP quality filters

The SNP datasets were filtered to only retain biallelic SNPs using the *view* and *filter* commands in BCFtools. VCFtools version 0.1.15 (Danecek et al. 2011) was used to filter the dataset to only retain SNPs with SNP quality scores (QUAL) ≥30 and recode genotype calls to NA where genotype quality scores (GQ) were <30 or where the coverage fell outside of minimum (<15) and maximum read depth thresholds. The maximum read depth filters varied according to the population dataset due to variation in the distribution of read depths across populations (see Appendix S3.2 for more details). A maximum depth of 100 was applied to the New Zealand and India datasets and 125 to the entire dataset.

#### Removal of replicates, admixed Australian samples, and samples with heterozygote excess

Ewart et al. (2019) suggests three introduction points in Australia (Melbourne, Sydney, and Gold Coast) with some levels of admixture in some populations (Northern NSW and southern Queensland). The inclusion of admixed individuals is known to affect some downstream analysis and assignment of admixed individuals to a single population can be arbitrary (Pritchard et al. 2000; Luu et al. 2017). Further, the introduction of mynas to New Zealand likely occurred in the 1870s and 1880s before the establishment of some of the other populations in Australia. To simplify global population structure analyses and the identification of introduction source(s) of New Zealand mynas, we used BCFtools to retain only Australian samples from Melbourne, Sydney, and Gold Coast in the ALL dataset prior to further filtering and subsampling (see Fig. S1.1 for locations of Australian samples retained).

One sample from each set of the replicates (including DArTseq internal replicates) was retained at random prior to further SNP filtering. A further three samples (two from Sydney, and one from Odisha) were removed from the ALL and IND dataset due to the samples exhibiting heterozygote excess (highly negative *F*_*IS*_) (see Appendix S3 for more details). A total of 226, 77, and 467 individuals were retained in the NZ, IND, and ALL datasets respectively.

#### SNP data missingness, and singletons/doubletons

NZ, IND, and ALL datasets were filtered to remove SNPs with >20% missingness using BCFtools. For data used in the population structure analyses, singletons and doubletons (SNPs only occurring in one sample) were removed from the dataset using VCFtools to remove potential artefactual alleles (see section 3.5 and 3.6 in Appendix S3 for more details). For data used for calculating the site frequency spectrum, filters for singletons and doubletons were not performed to prevent introducing SNP ascertainment bias and altering the site frequency spectrum.

#### Linkage disequilibrium

We calculated pairwise linkage disequilibrium (LD) and plotted this against pairwise distance using PopLDdecay (Zhang et al. 2019) to visualise LD in our datasets (Figs. S4.1–4.3). To remove SNPs in LD, we retained 1 SNP per every 100,000 base pairs when found on the same reference contig using the --thin option in VCFtools (see Appendix S4 for more details). After thinning, we retained 5474, 5978, and 5904 SNPs in the NZ, IND, and ALL dataset, respectively.

#### Population delineations

Further downstream analyses, such as population pairwise-*F*_*ST*_ calculations and tests for Hardy Weinberg Equilibrium (HWE), require prior knowledge of the population structure. However, incorrect delineation of populations may result in inaccurately clumping genetically distinct populations, or separating genetically homogenous populations.

Consequently, initial principal component analysis (PCA) and sparse non-negative matrix factorisation (sNMF) population genetic structure analysis were performed on the NZ, IND, and ALL dataset prior to HWE filtering to avoid the inadvertent introduction of a Wahlund effect (Pearman et al. 2022). PCA was performed and visualised using the ‘dartR’ R package version 1.9.9.1 (Gruber et al. 2018). sNMF was performed using the ‘LEA’ R package version 3.2.0 (Frichot and François 2015) with default settings, regularisation parameter (α)= 100, and 10 replicates per K value (number of genetic groups) and plotted using the ‘pophelper’ R package version 2.3.1 (Francis 2017).

Based on these preliminary population clustering results, a total of 32 populations were delineated for further analyses. These included separating the six individuals from Maharashtra (India) as a separate population (‘IND: Maharashtra subpopulation A’ or ‘Maharashtra subpopulation A,’ hereafter) from other Maharashtra individuals due to separate clustering. Other populations were also separated based on the location and timing of the sample collection (samples from the ROM were collected in 1970s–1980s, while the rest were collected in 2014–2020). This delineation of populations will be referred to as ‘popdef1’ hereafter (see Tables S1.2 and S1.3 for details).

#### Hardy Weinberg Equilibrium

Using populations as delineated above (popdef1), loci were filtered to remove non-neutral loci by testing for HWE using the *HWExactMat* function from the ‘HardyWeinberg’ R package version 1.7.2 (Graffelman and Camarena 2008; Graffelman 2015), which tests for departure from HWE using the Fisher’s exact test. All loci with *p* values < 0.01 in any population were removed from the dataset. A *p* value threshold of < 0.01 was chosen instead of a correction for multiple testing because the distributions of the *p* values did not follow a uniform distribution (i.e., the *p* value distribution had a peak near 1 and 0; see Appendix S6). All subsequent analyses except for the site frequency spectrum calculations were conducted with the HWE filtered SNP dataset (5037, 5900, 5584 SNPs for the NZ, IND, ALL dataset).

#### Subsampling of the global (ALL) dataset for further PCA analyses

Uneven sample sizes across genetic populations have been shown to potentially affect analyses such as principal component analysis (PCA) (Privé et al. 2020) and population structure analyses (Puechmaille 2016). Therefore, we explored the effects of subsampling and the delineation of populations in the global (ALL) dataset. PCA on full and subsampled datasets suggests that there is an effect of uneven sampling in the ALL dataset, distorting the principal components and inflating the differences between the more well-sampled and inbred populations (i.e., Australian and New Zealand samples) and the other populations (Appendix S5).

Consequently, samples in the ALL dataset from the same geographic region that were genetically very similar based on the preliminary clustering analyses (section 2.3.5, above) were grouped together and populations were delineated as follows for subsampling: IND: Maharashtra subpopulation A, IND: Other, AUS: Melbourne, AUS: Sydney, AUS: Gold Coast, NZ: Napier, NZ: Other, South Africa, Fiji and Hawaii. This delineation of populations will be referred to as ‘popdef2’ hereafter (see Tables S1.2 and S1.3 for more details). We explored different subsampling strategies (Appendix S5), and decided to subsample the ALL dataset so that we retained 20 individuals per introduced population when we performed the PCA. The uneven sampling is not expected to affect the results of other analyses so the full ALL dataset was used for these (see below). Further, given relatively even sample numbers across populations within India and New Zealand, we did not subsample the IND and NZ datasets for PCA.

### Population structure

PCA analyses were repeated on the HWE-filtered SNP datasets using the popdef2 delineated populations. Unlike ADMIXTURE and STRUCTURE, sNMF does not assume HWE. However, the final sNMF analysis were performed on the HWE-filtered dataset to ensure that we were detecting neutral population structure. We also performed both PCA and sNMF on different subsets of the dataset (MAF > 0, MAF > 0.05, MAF > 0.1) to ensure consistency of results (Appendix S8.6 and S8.7).

Inter-population genetic differences with the HWE-filtered dataset were examined across the dataset using pairwise-*F*_*ST*_ values calculated using the *gl.fst.pop* function in the ‘dartR’ R package (100 bootstraps performed) for all population combinations.

Isolation by distance analysis based on the Mantel test (IBD) was performed using the *gl.ibd* function in the ‘dartR’ R package to determine whether genetic differentiation between populations within India and within New Zealand can be explained by geographical distance. To do so, we correlated the transformed population pairwise-*F*_*ST*_ (${F}_{{ST}}/(1-{F}_{{ST}})$) and the natural log-transformed geographical distance (km) (Rousset 1997). Mantel tests were performed on the populations from India and the populations from within the NZ: Other cluster with more than five individuals. Given high genetic differentiation between Maharashtra and Maharashtra subpopulation A (from the same sampling location), we excluded Maharashtra subpopulation A from the Mantel test.

### Genetic diversity

Genetic diversity indices were calculated for each population using the HWE-filtered dataset. In the NZ and IND dataset, the popdef1 population delineation was used. For the ALL dataset, both the popdef1 and popdef2 delineations were used in order to investigate the fine-scale and broader-scale diversity patterns across India and New Zealand. Mean observed and expected heterozygosity were calculated using the *gl.report.heterozygosity* function in the ‘dartR’ R package. The proportion of polymorphic loci was calculated after randomly subsampling the dataset to n = 6 per population, 100 times. This subsampling accounts for the uneven sampling per population, as populations with more samples will likely have more polymorphic loci. Mean allelic richness was calculated using a rarefaction method to account for uneven sampling using the *allel.rich* function in the ‘PopGenReport’ R package version 3.0.4 (Adamack and Gruber 2014; Gruber and Adamack 2015). Mean allelic richness, and private allelic richness were also calculated using another rarefaction method using the software HP-rare version 1.1 (Kalinowski 2005). Finally, the shape of the folded site frequency spectrum was used alongside other genetic diversity metrics to visualise the genetic bottlenecks experienced in the different introduced populations. The folded site frequency spectrum (SFS) was plotted based on the minor allele frequency calculated by the *gl.percent.freq* function in the ‘dartR’ R package for the different populations within the ALL dataset, subsampling to n = 6 individuals per population and repeating the subsampling 100 times.

## Results

### Population structure

#### New Zealand dataset

**Fig. 2:**
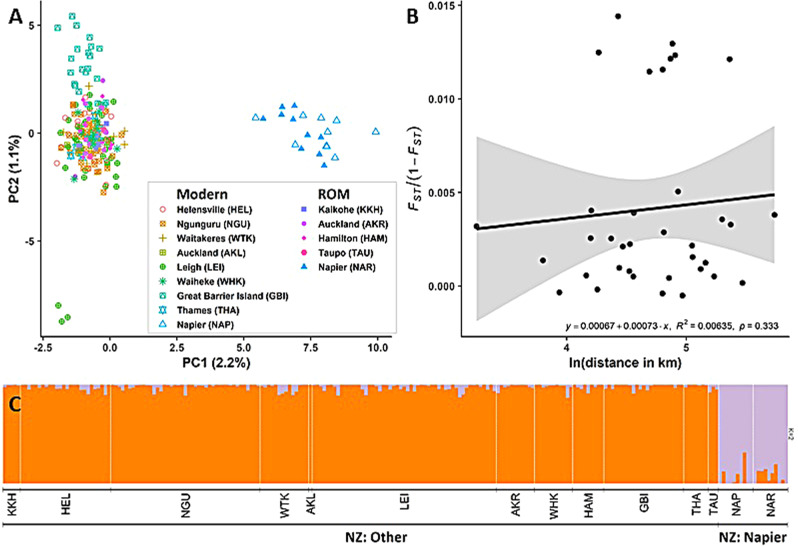
Population structure analyses for the New Zealand (NZ) dataset. **A** PC1 vs PC2 for the NZ dataset, based on 5037 SNPs. Colour and shape of points represent sample location and time of collections. ROM = Royal Ontario Museum samples which were collected between 1975–1989. Other samples were collected between 2017–2020. Axes are labelled with the variance explained. **B** Mantel test for samples from NZ: Other, excluding populations with fewer than 5 individuals, and sample M0367 from Auckland which had different sample locations from other Auckland samples. **C** Population structure estimated by sNMF analysis, using K = 2. Three-letter codes labelled beneath the figure (e.g. KKH, HEL, NGU, etc.) represent location and time of collection as labelled in (**A**).

The PCA of the New Zealand-only (NZ) dataset shows two clusters on PC1 (Fig. 2A): individuals from Napier, and individuals from the rest of New Zealand. Within the ‘rest of New Zealand’ cluster, three individuals from Leigh separate on PC2. Based on pairwise relatedness values, these represent a trio of likely first-degree relatives. Note that close relatives were not removed from the dataset to avoid misrepresenting the overall diversity within populations. The sNMF population structure analysis was best explained with K = 2 (Fig. 2C) and supports the two populations as shown by the PCA. In agreement with the population structure analysis, the Napier populations have moderate levels of genetic differentiation from all other New Zealand populations (pairwise-*F*_*ST*_ = 0.05–0.07), with low but significant differentiation between the ROM (collected in 1984) and modern (collected between 2017–2020) samples (pairwise-*F*_*ST*_ = 0.015; Fig. S8.5). The Great Barrier Island (GBI) population shows low but significant differentiation (pairwise-*F*_*ST*_ = 0.010–0.014) from the other New Zealand populations. Pairwise-*F*_*ST*_ values among the other New Zealand population are generally very low (pairwise-*F*_*ST*_ < 0.005) and/or insignificant (*p* values > 0.05).

The Mantel test on the populations within NZ: Other (excluding populations with *n* ≤ 5) found no significant isolation by distance (Fig. 2B, *R*^2^ = 0.00635, *p* = 0.333). The cluster of points with ${F}_{{ST}}/(1-{F}_{{ST}})$ > 0.01 is a result of the inclusion of GBI; removal of the GBI population did not result in significant isolation by distance (Fig. S8.4).

#### Indian dataset

**Fig. 3:**
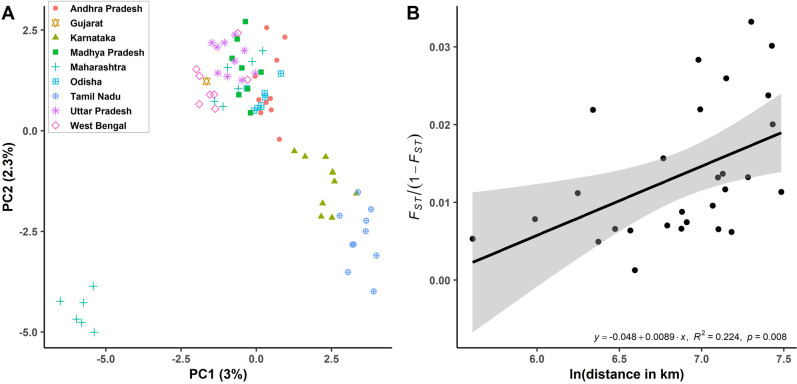
Population structure analysis for the Indian (IND) dataset. **A** PC1 vs PC2 for the IND dataset, based on 5900 SNPs. Axes are labelled with the variance explained. The lower left cluster of samples is a subpopulation, termed ‘subpopulation A’ of Maharashtra samples. **B** Mantel test for samples from India, excluding the samples from Gujarat (*n* = 1) and Maharashtra subpopulation A.

PC1 vs PC2 from the native-range India-only (IND) dataset shows two to four clusters (Fig. 3A). The most distinct cluster consists of samples from Maharashtra subpopulation A. The sNMF population structure analysis of the IND dataset was best explained with K = 1 (Fig. S8.11, see Fig. S8.12 for population structure plots at K = 2–5). With the exclusion of Gujarat (*n* = 1), and Maharashtra subpopulation A, a Mantel test on the populations within India found significant isolation by distance (Fig. 3B, *R*^*2*^ = 0.224, *p* = 0.008).

Population pairwise-*F*_*ST*_ values suggest low (*F*_*ST*_ = 0.001–0.03) but significant differentiation between all Indian populations except for Maharashtra subpopulation A which showed moderate levels of genetic differentiation from other Indian populations (*F*_*ST*_ = 0.042–0.073) (Fig. S8.13).

#### Global dataset

**Fig. 4:**
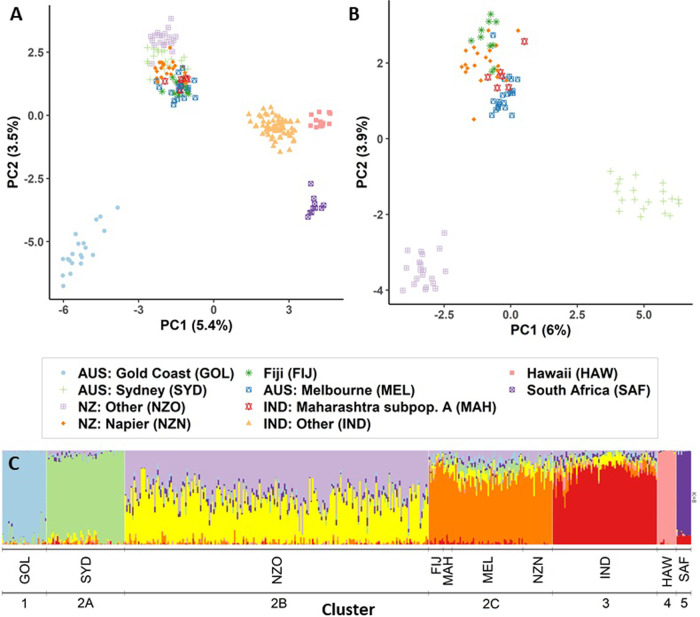
Population structure analyses for the global (ALL) dataset. **A** PC1 vs PC2 for the ALL dataset, number of individuals subsampled to *n* = 20 per introduced populations, based on 5572 SNPs. Colour and shapes of points represent sample locations. **B** PC1 vs PC2 for the ALL dataset with only populations that clustered closely with New Zealand. This includes NZ: Other, NZ: Napier, Fiji, AUS: Melbourne, AUS: Sydney and IND: Maharashtra subpopulation A. In (**A**) and (**B**), axes are labelled with the variance explained. **C** Population structure estimated by sNMF analysis, using *K* = 8. Three-letter codes are abbreviations of population names, as shown in the legend of the PCA plots. The cluster number refers to the genetic clusters described in text as shown by the PCA and the sNMF population structure plot.

The global (ALL) dataset was subsampled due to the large variation in sample sizes from different countries. Across 10 replicates, PCA on this subsampled data showed consistent clustering of the populations (results not shown), with a representative run presented in Fig. 4A. PC1 vs PC2 from this subsampled dataset shows five clusters (Fig. 4A): 1) samples from the Australian Gold Coast, 2) samples from New Zealand (NZ: Napier and NZ: Other), AUS: Melbourne, AUS: Sydney, Fiji and IND: Maharashtra subpopulation A, 3) samples from the rest of India, 4) samples from Hawaii (clustering closely to cluster 3), and 5) samples from South Africa. PC1 vs PC2 on just samples from cluster 2 show three clusters (Fig. 4B): 2A) samples from Sydney, 2B) samples from NZ: Other, and 2C) samples from NZ: Napier, Melbourne, Fiji, and Maharashtra subpopulation A. The numbering of the clusters refers to the labels in Fig. 4C.

The sNMF population structure analysis of the ALL dataset was best explained with K = 8, and supports seven genetic clusters (Fig. 4C). The seven genetic clusters/groups are: 1) Gold Coast, 2A) Sydney, 2B) NZ: Other, 2C) Melbourne, Fiji, Napier, and Maharashtra subpopulation A, 3) IND: Other, 4) Hawaii, and 5) South Africa. These groupings are very similar to the five clusters identified in the first PCA (Fig. 4A), but split PCA grouping 2 into the three sNMF clusters (2A-C) which were also identified from the second PCA (Fig. 4B).

**Fig. 5:**
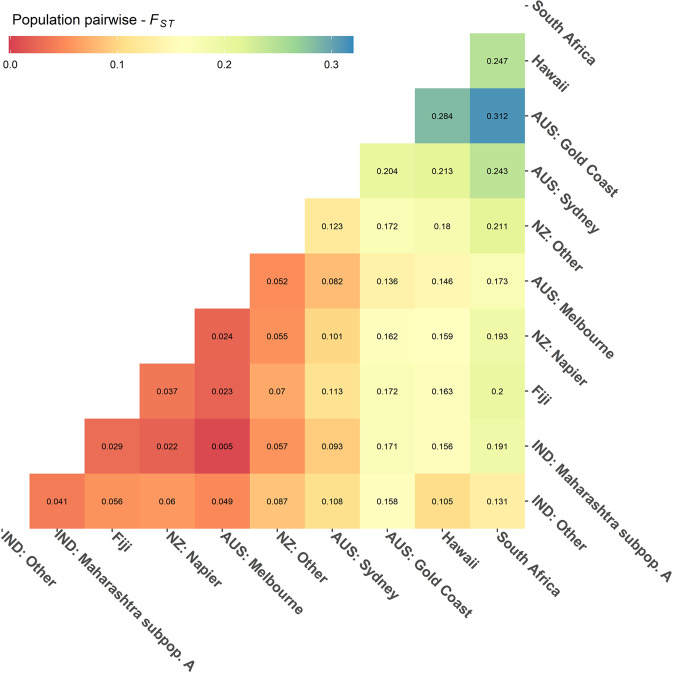
Heat map of mean population pairwise-*F*_*ST*_ from 100 bootstrapped pairwise-F_ST_ comparisons between all populations in the global (ALL) dataset. Populations were delineated in line with PCA and sNMF (Fig. 4, popdef2). Maharashtra subpopulation A was delineated as a separate population from other Indian samples as it clusters closely with AUS: Melbourne, NZ: Napier, and Fiji. All *F*_*ST*_ values have *p* values < 0.01.

Population pairwise-*F*_*ST*_ between all populations showed high genetic differentiation (pairwise-*F*_*ST*_ > 0.247) among distant bottlenecked populations (Gold Coast, Hawaii, and South Africa) and low genetic differentiation (pairwise-*F*_*ST*_ = 0.005–0.037) between Maharashtra subpopulation A and Melbourne, Napier, and Fiji (Fig. 5) – lower than genetic differentiation between Maharashtra subpopulation A and IND: Other (pairwise-*F*_*ST*_ = 0.041).

Maharashtra subpopulation A, Melbourne, Napier, and Fiji (Fig. 4, cluster 2C) show very low genetic differentiation (pairwise-*F*_*ST*_ < 0.05), comparable to populations from IND: Other (e.g. Odisha, Karnataka, etc.), and NZ: Other (e.g. Leigh, Waiheke, Thames, etc.) (see Fig. S8.18 for more details).

Population pairwise-*F*_*ST*_ between Hawaii and South Africa showed high genetic differentiation (pairwise-*F*_*ST*_ = 0.247). However, both populations showed lower population pairwise-*F*_*ST*_ with IND: Other (pairwise-*F*_*ST*_ = 0.105–0.131) than with other populations in this study.

### Genetic diversity

All genetic diversity indices (mean observed heterozygosity (H_o_), mean expected heterozygosity (H_e_), mean allelic richness (AR), proportion of polymorphic loci and mean private allelic richness (PAR)) inferred that myna populations in India were more diverse than the introduced populations (Table 1), except for Maharashtra subpopulation A which is the least diverse among the native populations and shows similar diversity metrics to the Melbourne population. Within the introduced populations, the Melbourne population appeared to be most diverse, followed by Fiji/Napier, NZ: Other, Sydney, Hawaii, and South Africa and Gold Coast (Table 1).

**Table 1:** Genetic diversity indices in the different populations.

Population	H_o_	H_e_	Mean allelic richness		Mean private allelic richness^b^	Median proportion of polymorphic loci
			*R*^a^	HP-rare^b^		
IND: Other	0.103	0.107	1.356	1.360	0.098	0.391
AUS: Melbourne	0.098	0.097	1.291	1.294	0.009	0.313
IND: Maharashtra subpop. A	0.094	0.088	1.262	1.290	0.008	0.312
NZ: Napier	0.093	0.093	1.274	1.281	0.007	0.300
Fiji	0.095	0.089	1.262	1.276	0.008	0.296
NZ: Other	0.089	0.090	1.257	1.257	0.009	0.272
Hawaii	0.085	0.083	1.227	1.234	0.039	0.244
AUS: Sydney	0.083	0.084	1.225	1.227	0.010	0.238
South Africa	0.077	0.074	1.189	1.195	0.033	0.202
AUS: Gold Coast	0.071	0.070	1.183	1.185	0.003	0.193

Populations were delineated in-line with PCA and sNMF (Fig. 4, popdef2). H_o_ = mean observed heterozygosity, H_e_ = mean expected heterozygosity.

^a^Calculated using the ‘PopGenReport’ R package.

^b^Calculated using the rarefaction method in HP-rare software with number of alleles = 10. The table is sorted with the population with the highest proportion of polymorphic loci first.

**Fig. 6:**
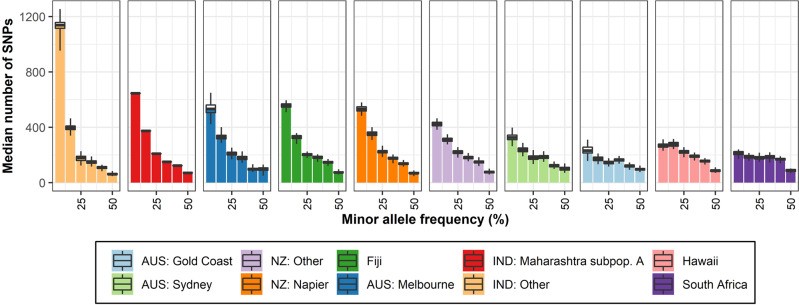
Folded site frequency spectrum (SFS) plot of each of the populations. Populations were delineated in-line with PCA and sNMF (Fig. 4, popdef2). All populations were subsampled to six individuals, error bars indicate standard errors over 100 replicates samples. Population colours correspond to the population colours in Fig. 4A, B.

Similar to the other genetic diversity indices, the shape of the SFS plot (Fig. 6) suggests that the IND: Other population is most diverse, followed by Maharashtra subpopulation A, Melbourne/Fiji, Napier, NZ: Other, Sydney, and Hawaii/South Africa/Gold Coast. Note that IND: Other and NZ: Other are a combination of samples from multiple locations and may alter some statistics (see Tables S9.1–9.3 and Fig. S9.1 for results with populations delineated based on popdef1 population delineations).

## Discussion

The spread of invasive species may leave characteristic genetic signatures across the landscape. Given appropriate sampling, the source population(s) and population structure can be identified and compared to historical records and current observations. The differences in genetic diversity, differentiation, and composition between populations can elucidate the connectivity between populations and population introduction history. Using common myna samples from the native and the invasive range, we were able to utilise genomic analyses to identify the population structure, and evaluate and infer the myna introduction history from India to Australia and then to New Zealand.

### Population structure, genetic diversity, and introduction history

Our extensive sampling from the native and invasive range has allowed, for the first time, inference of the origin of mynas introduced to New Zealand. Historical records suggests that mynas in New Zealand were founded by individuals from an established population in Australia which in turn was founded by individuals from the native range in India (Thomson 1922). The clustering of New Zealand mynas with Melbourne samples strongly supports importation of birds from Melbourne, and is in agreement with analysis of primary literature from the time of introduction (Beesley et al. 2023). The clustering of mynas from Melbourne and from Fiji with Maharashtra subpopulation A also suggests that the birds in Melbourne and Fiji were likely founded by individuals from Maharashtra.

Two genetic clusters in New Zealand were identified that appear to be consistent with the historical record of the species introduction and the distribution in New Zealand (Cunningham 1948; Robertson 2007; Heather and Robertson 2015). The two populations appear to be divided by North Island’s axial mountain ranges (Tararua, Ruahine, Kaweka, Kaimanawa and Raukūmara ranges), with the population to the east of the mountain range from Masterton to East Cape represented by the more genetically diverse samples from NZ: Napier, while the population to the west from Palmerston North to Northland is represented by samples from the rest of New Zealand (NZ: Other) (Figs. 1C and 2, and Tables S1.2, and S1.3).

Historical records suggest that mynas were introduced to Napier in the 1870s/1880s and therefore represent an early population establishment (Acclimatisation 1877; ‘Hawke’s bay acclimatisation society.’ 1877; Cunningham 1948), while the populations in the rest of the North Island likely established later by northward colonisation from the Wellington, Whanganui or New Plymouth region (2, 3, and 4 in Fig. 1C) where they were first introduced in the 1870s/1880s (Thomson 1922; Long 1981; Beesley et al. 2023). Mynas only started to appear in the Waikato and Auckland region in the 1920s and only established themselves in these regions in the 1940s (Cunningham 1948). Cunningham (1948) did not report mynas north of Auckland, or from Waiheke and Great Barrier Island where some of our samples were collected. While Cunningham (1948) described four main areas of the myna distribution within the North Island, the 1947 distribution map in the study showed the two New Zealand populations west and east of the axial mountain range being clearly separated, and more so than in more recent distribution maps (Robertson 2007; Heather and Robertson 2015; Beesley et al. 2023).

Among the New Zealand populations, Great Barrier Island population (GBI) shows low but significant levels of genetic differentiation from other New Zealand populations (Fig. S8.5, pairwise-*F*_*ST*_ = 0.010–0.014). Mynas were only first recorded on GBI in 1960 (Bell and Brathwaite 1964), and were widespread by 1975 (Bell 1976; Ogle 1981). GBI clearly nests within NZ: Other in our population structure analyses but SFS and genetic diversity metrics show that GBI has the lowest diversity of any of our New Zealand sampling sites and is likely to have suffered some genetic bottlenecking and drifted from NZ: Other that may have founded it (Fig. S9.1, and Table S9.1). Continual gene flow with the mainland NZ: Other population may remove this exhibited bottleneck effect in the future. We investigated gene flow among the New Zealand populations (n ≥ 20) within NZ: Other, and between the Napier and Leigh population (representative of the NZ: Other population) using BA3-SNPs version 3.04 (Wilson and Rannala 2003; Mussmann et al. 2019), but were unable to detect any gene flow. However, it is likely the populations in our analysis are not differentiated enough for BA3-SNPs to deduce gene flow (see Appendix S7.2 for more details).

While NZ: Other is identified as a separate genetic cluster in the sNMF analysis (cluster 2B), it is most genetically similar to sNMF cluster 2C (pairwise-*F*_*ST*_ = 0.052, 0.055, 0.057 and 0.07 with Melbourne, Napier, Maharashtra subpopulation A, and Fiji, respectively). This clustering, and given that Napier has higher genetic diversity than NZ: Other, raises the hypothesis that Napier and NZ: Other were separate introductions from Melbourne, versus NZ: Other being established from Napier birds. Attempts were made to resolve the population history with phylogenetic trees and coalescent simulations using fastsimcoal2 version 2.7.09 (Excoffier et al. 2021), but key nodes were not well supported and the different scenarios in the coalescent simulations did not differ significantly (see Appendix S7.1 for more details). This may be due to the shared population history and very similar and recent coalescent times for each of the scenarios. Nonetheless, historical records, New Zealand’s proximity to Australia, Sydney population’s lower genetic diversity compared to NZ: Other, and the lower genetic differentiation between NZ: Other and AUS: Melbourne populations would suggest that mynas in NZ: Other may have also been founded by individuals from Melbourne (rather than Sydney) but have suffered a more severe genetic bottleneck compared to the Napier population.

Sydney is identified as a separate genetic cluster in the sNMF analysis (cluster 2A) and is most genetically similar to Melbourne, (pairwise-*F*_*ST*_ = 0.082 in Fig. 5, and pairwise-*F*_*ST*_ = 0.081–0.089 in Fig. S8.18). Interestingly, this suggests that, like mynas in New Zealand, mynas in Sydney may have also been founded by individuals from Melbourne. This result provides further context to the findings of Ewart et al. (2019), who found that Sydney and Melbourne were genetically distinct and likely represented two introduction points but, in the absence of data from the native range nor clear historic records, could not differentiate between Sydney being established from a separate introduction from India, or a translocation from Melbourne. Although potentially resolving the ultimate origin of Sydney and Melbourne populations, the present study does not resolve the origin of the Gold Coast population that perplexed Ewart et al. (2019). The high level of genetic distinctiveness in the Gold Coast population observed in this study implies either introduction from an as yet unidentified source, or extreme bottlenecking in a Melbourne-derived population.

Hawaii and South Africa showed high levels of genetic differentiation between each other, but both populations show lower genetic differentiation with IND: Other than with other populations in this study. This suggests that mynas in Hawaii and South Africa may have been independently founded by individuals from IND: Other.

### Implications and future analyses

Mynas in New Zealand appear to have a clear population structure and are divided into two populations, east and west of North Island’s mountain range. While the two populations may share a common origin, the population in the east (NZ: Napier) is more diverse and has a smaller distribution. Further analyses can be built upon this study to provide more information for management of the species in New Zealand. Our gene flow analysis was unsuccessful, but further analyses on higher-density sequencing data (e.g. to enable inference of ancestral haplotypes) may help resolve the very recent establishment of the New Zealand populations. Additional samples from more locations will also help elucidate finer scale population structure, and reduce the chance that key populations are not sampled. For example, samples from the highways crossing the North Island axial mountain range can potentially help deduce if there is admixture between the NZ: Other and NZ: Napier populations, and if this requires any management interventions. Similarly, tracking individuals in key populations may provide an alternative source of information on the species’ dispersal ability and population connectivity, which may further help inform myna management programmes on the feasibility of myna eradication in some locations (e.g. island populations).

The identification of Maharashtra subpopulation A as the potential source of the mynas in Australia and Fiji also raised other questions regarding this potential source population. Maharashtra subpopulation A is genetically less diverse than the other Indian populations and has potentially experienced a population bottleneck. These samples were collected in 1975 from Maharashtra, although the exact location is unknown. Other samples from Maharashtra, and the rest of India were also collected from a similar time period (a West Bengal sample and two other Maharashtra samples were collected in 1975; other Indian samples, in 1983; see Table S1.2 for more details). Why is there a more bottlenecked population within the more-or-less panmictic Indian population? Where are these samples exactly from? To answer these questions, additional samples from the potential source populations of Maharashtra should be analysed.

We note that this dataset includes samples collected from the same locality from different time periods (Sydney, Melbourne, and Napier). In all cases, we found low but significant pairwise-*F*_*ST*_ between the samples collected in 1970s–1980s (ROM) and the modern samples collected in 2014–2020, suggesting that the genetic composition of mynas in these populations may have changed since the collection of the ROM samples. Although there may be some bias from sample size differences, in addition to variability introduced from stochastic sampling effects and differences between sampling locations, it is likely that much of this signal is due to drift and population size changes. Interestingly, unlike Melbourne and Napier where samples from different time periods show similar levels of genetic diversity, modern Sydney samples show higher genetic diversity compared to Sydney ROM samples, and modern Sydney samples are more genetically similar to modern Melbourne (and Melbourne ROM) than Sydney ROM (Fig. S8.18). These results may point to recent gene flow between Melbourne and Sydney, but equally could represent differences between sampling locations between the two timepoints. However, some changes between populations over time may have occurred in response to changing selection pressures (e.g. increased urbanisation, adaptation to the newly invaded environment, (Baker and Moeed 1979)). Analyses in the future may be able to identify candidate genes for adaptive traits that are under selection in the invasive populations, and help provide a biological explanation for their invasive success.

This study presents the most extensive population genomic studies of the common myna to date, which utilises samples from multiple locations across the world. We were able to identify the source population of mynas in a few invasive locations: populations in Melbourne and Fiji were likely founded by individuals from Maharashtra, and populations in New Zealand were likely founded by individuals from Melbourne. We were able to identify two distinct populations in New Zealand, east and west of North Island’s axial mountain ranges. This observation confirms previous observations that mountain ranges and thick forests may form barriers for myna dispersal and provide useful information for the species management. These findings support the use of thick forests and mountains as barriers of management units and a more localised management strategy of mynas in New Zealand (East and West of the North Island’s axial mountain range). This also highlights the potential additional benefits of reforestation programmes in New Zealand (e.g. One Billion Trees Programme (Te Uru Rākau 2020)). However, it must be noted that not all forest types may form barriers for myna dispersal, and further research would be needed to determine the forest features that act as barriers (e.g. old versus new growth forests, forest patch size). The population structure in New Zealand also allows management programmes to possibly identify the source(s) of newly established populations (e.g. populations south of 40°S), and vagrants, and limit their invasion pathways. With decreasing cost of sequencing, improving sequencing capabilities, and increasing availability of reference genomes, the identification of the source population(s), the introduction history, and the population structure from this study provides a strong basis for more detailed studies of the species in the future (e.g. adaptation genomics, genome-wide association scans, and gene-environment association analyses). This study has highlighted a common challenge faced in genetic studies of most invasive species – uneven sampling involving inbred populations and population delineations - and demonstrated how this issue may be handled. This study also demonstrates the value and utility of museum collections to address present-day challenges such as invasive species.

## Supplementary information

Supplementary material

Table S1.2

## Data Availability

All scripts used in data processing and analysis are available on GitHub (https://github.com/akamolphat/myna_popgen). Some code is also available in the Supplementary materials. Individual metadata can be found in Table S1.2 in the Supplementary Materials. Raw reads, barcodes for demultiplexing, and processed variant data (VCF format) can be found on Dryad Digital repository (10.5061/dryad.xsj3tx9m7).
