# Supplementary material for: Tracing the introduction of the invasive common myna using population genomics
Source: Heredity (Edinb). 2023 May 17;131(1):56–67. doi: 10.1038/s41437-023-00621-w (PMC10313710; doi:10.1038/s41437-023-00621-w)
Supplement: Supplementary file 1 — Supplementary material [file 41437_2023_621_MOESM1_ESM.docx]

Supplementary materials for:

**Tracing the introduction of the invasive common myna using population genomics.**

Kamolphat Atsawawaranunt^1^, Kyle M. Ewart^2,3^, Richard E. Major^2^, Rebecca N. Johnson^2,4^, Anna W. Santure^1^, Annabel Whibley^1^

^1^ School of Biological Sciences, University of Auckland, Auckland, New Zealand

^2^ Australian Museum Research Institute, Australian Museum, Sydney, NSW, Australia

^3^ School of Life and Environmental Sciences, University of Sydney, Sydney, NSW, Australia

^4^ National Museum of Natural History, Smithsonian Institution, Washington D.C., District of Columbia, USA

# Appendix S1 Sample details

Table S1.1 summarises the number of individuals from each country and their sources. Table S1.2 outlines the location information, year of sampling (if available), and the data sources of each sample.

Table S1.1 Summary of sample origin and sources. ^1^Royal Ontario Museum, ^2^Australian Museum (Ewart et al., 2019), ^3^Data contributors in New Zealand

| **Country** | **Number of individuals sampled (source 1, source 2)** | **Sources** |
| --- | --- | --- |
| Australia | 477 (26, 451) | 1, 2 |
| Fiji | 10 | 1 |
| Hawaii | 13 | 1 |
| India | 78 | 1 |
| New Zealand | 226 (33, 193) | 1, 3 |
| South Africa | 10 | 1 |
|  | | |

Table S1.2 Sample information, location, time of collection, and population delineations

Table S1.2 can be found in a separate file in the supplementary information (TableS1.2.xlsx).

Table S1.3 Summary of population delineations and how popdef1 and popdef2 relates to each other. All samples from India, South Africa, Hawaii, and Fiji are from the Royal Ontario Museum (ROM). ROM is labelled in popdef1 in countries where there are also modern samples (2014-2020). Number of samples (n) is the total number in the ALL dataset after removal of replicate samples, samples from admixed Australian populations and outlier samples.

| **Country** | **Location** | **popdef2** | **popdef1** | **n** |
| --- | --- | --- | --- | --- |
| India | Andhra Pradesh | IND: Other | Andhra Pradesh | 10 |
|  | Gujarat |  | Gujarat | 1 |
|  | Karnataka |  | Karnataka | 10 |
|  | Madhya Pradesh |  | Madhya Pradesh | 8 |
|  | Odisha |  | Odisha | 7 |
|  | Tamil Nadu |  | Tamil Nadu | 10 |
|  | Uttar Pradesh |  | Uttar Pradesh | 10 |
|  | West Bengal |  | West Bengal | 8 |
|  | Maharashtra |  | Maharashtra | 7 |
|  |  | IND: Maharashtra subpop. A | Maharashtra subpop. A | 6 |
| Australia | Gold Coast | AUS: Gold Coast | Gold Coast | 30 |
|  | Melbourne | AUS: Melbourne | Melbourne | 40 |
|  |  |  | Melbourne (ROM) | 8 |
|  | Sydney | AUS: Sydney | Sydney | 35 |
|  |  |  | Sydney (ROM) | 18 |
| New Zealand | Auckland | NZ: Other | Auckland (ROM) | 11 |
|  | Great Barrier Island |  | Great Barrier Island | 23 |
|  | Hamilton |  | Hamilton (ROM) | 9 |
|  | Helensville |  | Helensville | 26 |
|  | Kaikohe |  | Kaikohe (ROM) | 5 |
|  | Leigh |  | Leigh | 53 |
|  | Ngunguru |  | Ngunguru | 43 |
|  | Auckland |  | Auckland | 1 |
|  | Taupo |  | Taupo (ROM) | 3 |
|  | Thames |  | Thames | 7 |
|  | Waiheke |  | Waiheke | 11 |
|  | Waitakeres |  | Waitakeres | 14 |
|  | Napier | NZ: Napier | Napier | 10 |
|  |  |  | Napier (ROM) | 10 |
| Fiji | Central Division | Fiji | Fiji | 10 |
| South Africa | Gauteng | South Africa | South Africa | 10 |
| USA | Hawaii | Hawaii | Hawaii | 13 |
| Total |  |  |  | 467 |


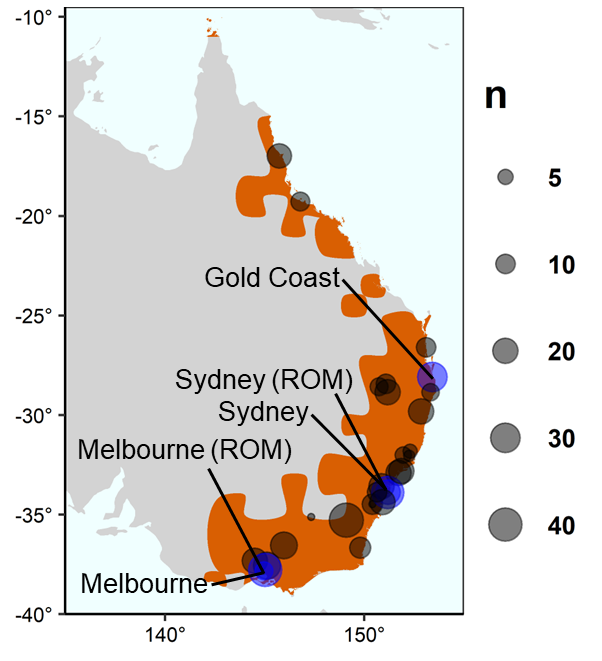


Figure S1.1 Map of locations of individuals sampled in Australia overlaid on top of the range distribution in orange (BirdLife International and Handbook of the Birds of the World, 2016). Locations of samples which were retained after removal of admixed Australian samples (section 2.3.2 of the main text) were labelled and marked in blue (Melbourne, Melbourne (ROM), Sydney, Sydney (ROM), and Gold Coast). For a map with labels of locations of other samples in Australia, see Figure 1 in Ewart et al. (2019) for more details.

# Appendix S2 Supplementary methods

In this analysis, datasets were generated from three SNP calling pipelines and are described as follows:

- DArT datasets – SNPs identified *de novo* using the DART P/L proprietary DArTsoft14 algorithm; reads were mapped to the draft myna genome to aid SNP calling.
- BCFtools datasets – SNPs resulting from the BCFtools pipeline; this represents the dataset used for analyses presented in the main text.
- STACKS datasets – SNPs resulting from the STACKS pipeline.

For each of the pipelines, there are three datasets: the dataset with only New Zealand samples (NZ dataset), the dataset with only India samples (IND dataset), and the dataset with all the samples (ALL dataset).

The main analyses in this study were only performed on the BCFtools dataset, and described in the main text. The overall framework is summarised in Figure S2.2. However, preliminary analyses were also performed on the DArT and STACKS datasets. The outline of the different pipelines is described in Figure S2.1. The STACKS and DArT pipelines are described in section S2.1 and S2.2, respectively.

Table S2.1 Summary of software used in this study.

| **Software and version** | | **Uses in this study** |
| --- | --- | --- |
| R packages | tidyverse 1.3.2 | Dataframe manipulation and plots |
|  | ggplot2 3.3.6 | Generating plots |
|  | dartR 1.9.9.1 | - Read DArT csv file and VCF file - Performed PCA and Mantel test - Calculated population pairwise-FST and observed and expected heterozygosity |
|  | vcfR 1.12.0 | Read parts of VCF files that were not read by dartR |
|  | cowplot 1.1.1 | Made panel plots |
|  | rgdal 1.5.32 | Read shapefiles for plotting maps |
|  | LEA 3.2.0 | Performed sNMF analysis |
|  | pophelper 2.3.1 | Plotted population structure plots (sNMF analysis outputs) |
|  | HardyWeinberg 1.7.2 | Performed Fisher's Exact test for departures from HWE |
|  | PopGenReport 3.0.4 | Calculated rarefied allelic richness |
|  | dismo 1.3.5 | Projected WGS1984 coordinates to Mercator projection |
| BCFtools 1.13 | | - Variant calling - Data filtering - Conversions between different VCF file types (i.e. BCF, VCF and vcf.gz) |
| VCFtools 0.1.15 | | - Data filtering - SNP thinning |
| SAMTOOLS 1.12 | | Sorted and indexed reads of samples |
| STACKS 2.58 | | - Removed barcodes and adapters - Variant calling |
| fastp 0.20.0 | | Removed barcodes and adapters |
| fastqc 0.11.9 | | Quality control checks |
| Multiqc 1.9 | | Quality control checks |
| BWA 0.7.17 | | Aligned reads to reference |
| HP-rare 1.1 | | Calculated rarefied allelic and private allelic richness |
| BA3-SNPs 3.0.4 | | Performed contemporary geneflow analyses |
| fastsimcoal2 2.7.09 | | Performed demographic inferences |

**Quality**

**filters**

**Datasets**

**SNP calling**

**pipelines**

DArTseq short-read sequences

DArTsoft14

DArT dataset filters

**4b.** BCFtools

PCA

**4a.** STACKS

DArT dataset

STACKS dataset

BCFtools dataset

**2.** Trim adapters

**1.** Remove barcodes

**3.** Align to reference

**5a.** STACKS dataset filters

**5b.** BCFtools dataset filters

**Linkage disequilibrium filters:** Keep 1 SNP per 100,000 bp

**Remove singletons and doubletons**

sNMF

**Main analyses on BCFtools datasets only (see Figure S2.2)**

fastsimcoal2

BA3-SNPs

**BCFtools dataset only**

**Identify population structure**

**Infer population history**

Figure S2.1 Flowchart of the overview of the methods used in this study, including the processing of the DArT, STACKS and BCFtools dataset.


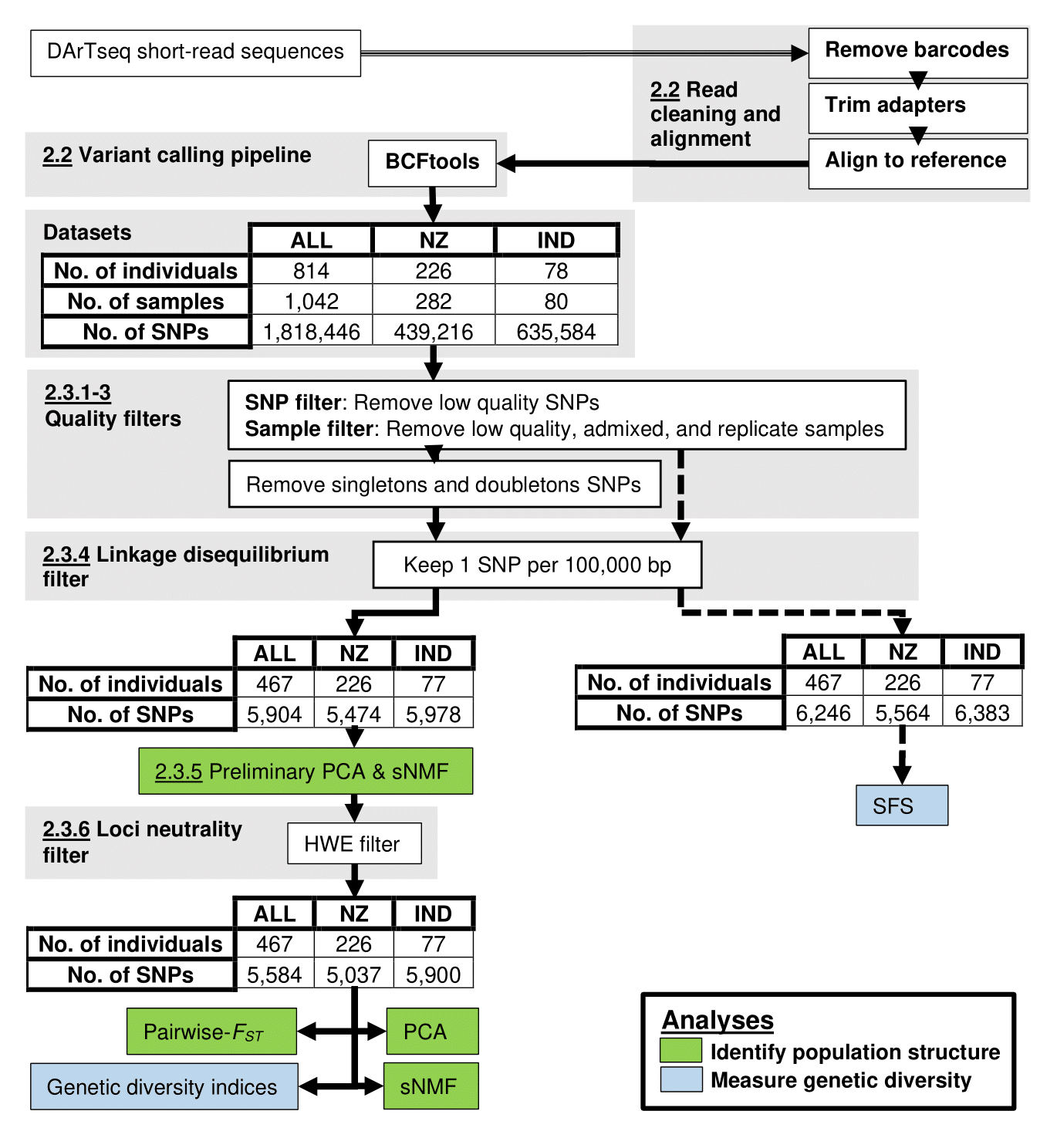


Figure S2.2 Flow chart of the overview of the methods used for the BCFtools SNP dataset. The number of individuals and SNPs after major filters can also be found on the flow chart. Parts of the flow chart corresponding with sections in the main text are annotated with the corresponding section number (e.g. 2.2).

**1. Remove barcodes**

The removal of barcodes and adapters, and the alignment of reads to the reference genome was performed for both the STACKS and the BCFtools pipeline. Barcodes were removed from raw reads of the samples we sent to dart using the *process_radtags* program in STACKS version 2.58 (Catchen *et al.*, 2013), using the following command:

process_radtags -p /path/to/folder/with/raw/reads -b /path/to/barcodesID.txt -o /path/to/output/folder1 -e pstI -c -q -r

# /path/to/folder/with/raw/reads is the path to the folder containing the raw reads

# /path/to/barcodesID.txt is the path to tab delimited txt file containing the barcode sequence in the first column (e.g. CCGTCTTATGCACT), and the sample ID in the second column (e.g. M0001). Example of the first 5 rows of this file:

# CCGTCTTATGCACT M0001a

# ACATGACCGACCT M0002a

# TGTTGCCGCGT M0003a

# TGCCGACAAGAA M0004a

# CACCGTCCATG M0005a

# /path/to/output/folder1 is the path to the folder where the reads with the barcodes removed will be stored

Barcodes were also removed from raw reads of the samples from the Australian Museum samples (Ewart *et al.*, 2019) using the *process_radtags* program in STACKS version 2.58 (Catchen *et al.*, 2013). However, this had to be done on each individual file at a time to avoid mixing issues as some barcodes were subset of other barcodes (e.g. ACATGACCGACCT for sample A, and ACATGACCG for sample B). This means that the process has to be looped over all the raw read file. This was executed using the following command:

process_radtags -f /path/to/raw/read/file.FASTQ.gz -b /path/to/sample/barcodesID.txt -o /path/to/output/folder1 -e pstI -c -q -r

# /path/to/raw/read/file.FASTQ.gz is the path to the sample raw read file.

# /path/to/sample/barcodesID.txt is the path to tab delimited txt file containing the barcode sequence in the first column (e.g. CCGTCTTATGCACT), and the sample ID in the second column (e.g. M0001). However, this is only for the particular sample. Example of the one of the file:

# CTGCT 12709_rep_b

# /path/to/output/folder1 is the path to the folder where the reads with the barcodes removed will be stored

**2. Trim adapters**

Adapters are trimmed using fastp version 0.20.0 (Chen *et al.*, 2018) with the following command. The command has to be looped over all files (barcodes removed from previous step):

fastp -l 40 --adapter_sequence=AGATCGGAAGAG -i /path/to/output/folder1/barcodes_removed.fq.gz -o /path/to/output/folder2/adapters_trimmed.fq.gz

# /path/to/output/folder1/barcodes_removed.fq.gz is the path to the file with the barcodes removed from the previous step.

# /path/to/output/folder2/adapters_trimmed.fq.gz is the path to the output file (barcodes removed and adapters trimmed).

**Quality control**

Quality control checks were performed using fastqc version 0.11.9 (Andrews, 2010) and multiqc version 1.9 (Ewels *et al.*, 2016). fastqc was performed on each file as output from the previous step while multiqc was performed on the outputs from fastqc using the following commands:

fastqc /path/to/output/folder2/adapters_trimmed.fq.gz -o /path/to/fastqc/folder

multiqc /path/to/fastqc/folder -o /path/to/multiqc/folder

# /path/to/fastqc/folder is the path to the folder containing the fastqc outputs

# /path/to/multiqc/folder is the path to the folder for the multiqc outputs

**3. Assembly of a reference and alignment of reads to reference**

Genomic DNA was extracted from a male myna collected in Newcastle, Australia using the Qiagen DNeasy blood and tissue kit. A Chromium Genome library v2 was prepared by Macrogen and sequenced in PE150 mode on a NovaSeq. A total of 110.7 Gb data was generated. A genome assembly was generated from a subsample of 260 million read pairs, targeting ~60x coverage using Supernova version 2.1.1. The resulting assembly was 1 044,988,094 bp in length, comprising 17,560 contigs with a contig N50 of 3.86 Mb.

Reads were then aligned to the draft common myna reference genome using BWA version 0.7.17 (Li and Durbin, 2009). This was done using the following command which has to be looped over all files.

bwa mem -R $(echo "@RG\tID:${i%.fq.gz}\tSM:${i%.fq.gz}\tLB:${i%.fq.gz}\tPL:ILLUMINA") $ref $i > /path/to/output/folder3/${i%.fq.gz}.sam

# This executed in the folder containing the files containing the reads with the barcodes removed and adapters trimmed.

# $i is the name of the individual file containing the barcodes removed and adapters trimmed reads.

# /path/to/output/folder3/ is the path to the output folder where the file with reads aligned to the reference would be stored.

The reads were then sorted and indexed using SAMTOOLS version 1.12 (Li *et al.*, 2009). This was done using the following command which has to be looped over all files.

samtools sort ${i%.fq.gz}.sam > ${i%.fq.gz}.sorted.bam

samtools index ${i%.fq.gz}.sorted.bam

samtools flagstat ${i%.fq.gz}.sorted.bam > ${i%.fq.gz}.flagstat.txt

# As above, $i is the name of the individual file containing the barcodes removed and adapters trimmed reads. These commands are to be executed at /path/to/output/folder3/

Variants were called based on three subsets of individuals – the entire dataset (ALL dataset: 814 individuals, 1042 samples), a New Zealand-only dataset (NZ dataset: 226 individuals, 282 samples) and an Indian-only dataset (IND dataset: 78 individuals, 80 samples). Note that the number of samples and individuals are different due to the inclusion/removal of replicates, including DArT-specific proprietary replicates which includes approximately 30% of the samples that were sequenced.

## BCFtools pipeline

Variants were called through the *mpileup* and *call* commands in BCFtools version 1.13 (Li, 2011). This refers to step 4b on the Figure S2.1, and section 2.2 in the main text and on Figure 2.

**bcftools mpileup**

bcftools mpileup -b list_of_file_name.txt -a "DP,AD,SP" --ignore-RG -Ou -f path/to/ref.fasta -o /path/to/mpileup_output.bcf

# list_of_file_name.txt is the path to file containing all the files with the aligned reads from step 3. Ideally this is executed in the directory contain the file and list_of_file_name.txt will only contain the file name

# path/to/ref.fasta is the path to the file with reference myna genome

# /path/to/mpileup_output.bcf is the path to the output .bcf file from bcftools mpileup

**bcftools call**

bcftools call can be executed on its own but the commands below rename the samples to remove file suffix before performing bcftools call.

# Make list of samples for renaming (remove .filtered.sorted.bam file suffix)

bcftools query -l /path/to/mpileup_output.bcf | awk -F'.filtered.sorted.bam' '{print $1}' > path/to/renamed_sample.txt

# Rename samples and pipe this into bcftools call

bcftools reheader -s path/to/renamed_sample.txt /path/to/mpileup_output.bcf | bcftools call -mv -Ob -o /path/to/variant_call_output.bcf -f GQ

# path/to/renamed_sample.txt is the path to the file with the sample name with the file suffix removed

# /path/to/variant_call_output.bcf is the path to the .bcf file containing all the variants as identified by bcftools call

## STACKS pipeline

Variants were called through the *gstacks* and *populations* program in STACKS version 2.58 (Catchen *et al.*, 2013). This refers to step 4a on the Figure S2.1. All SNPs identified in *gstacks* and *populations* were first output to a VCF file before further filtering with BCFtools version 1.13 (Li, 2011), and VCFtools version 0.1.15 (Danecek *et al.*, 2011).

**gstacks**

*gstacks* were executed using the reference-based mode as follows:

gstacks -I /path/to/output/folder3/ -S .filtered.sorted.bam -O /path/to/gstacks/folder/ -M /path/to/popmap_file.txt

# /path/to/output/folder3/ is the path to the folder containing .filtered.sorted.bam files which were output from step 3 (Align to reference)

# /path/to/gstacks/folder/ is the path to the folder where the outputs from gstacks will be stored

# /path/to/popmap_file.txt is the path to the population map giving the list of samples in the first column, and the population number code in the second column. The population number code can just be one number just to allow the program to work. Example of the first 5 rows of this file:

# 10201_rep_b 1

# 10201 1

# 10202_rep_b 1

# 10202 1

# 10203 1

**Populations**

The *populations* program reads in the outputs from *gstacks* and export the results in the specified format. The command below was used to export the variants identified to a VCF format:

populations -P /path/to/gstacks/folder/ -O /path/to/populations/folder/ --vcf

# /path/to/gstacks/folder/ is the path to the folder where the outputs from gstacks are stored

# /path/to/populations/folder/ is the path to the folder where the outputs from populations will be stored

The commands below were used to sort and index the VCF file for further use. Sorting of the VCF was done using the vcf-sort function in VCFtools version 0.1.15 (Danecek *et al.*, 2011):

cd path/to/populations/folder/

vcf-sort populations.snps.vcf | bgzip -c > populations.snps.sorted.vcf.gz

bcftools index populations.snps.sorted.vcf.gz

# populations.snps.vcf is the output from the populations program above. This is the standard output name from the populations program.

# populations.snps.sorted.vcf is the output is the indexed, sorted vcf file.

The STACKS datasets were filtered in the same way as the BCFtools datasets, except for the filter based on SNP quality scores (QUAL > 30). This is because the STACKS pipeline does not output the QUAL field. Read depth (DP) and genotype quality (GQ) filters were applied to the STACKS datasets in the same way as the BCFtools datasets, and were applied to the VCF files using the vcftools function in VCFtools with the minimum QUAL (--minQ, in the case of BCFtools datasets), minimum and maximum GQ (--minGQ, --maxGQ), and minimum and maximum DP (--minDP, --maxDP) flags (see section S3.1 and S3.2 for more details).

## DArT pipeline

DArT SNP data does not have read depth information per genotype call (DP), SNP/variant quality scores (QUAL) or genotype quality scores (GQ) and therefore was filtered differently from STACKS and BCFtools dataset. The DArT SNP dataset was filtered in R version 4.1.2 (R Core Team, 2021) using the ‘dartR’ R package version 1.9.9.1 (Gruber *et al.*, 2018). The filters were performed in the following order as follows:

- Remove replicate samples
- For the ALL dataset, only retain Australian samples from Melbourne, Gold Coast, and Sydney
- Remove samples with outlier heterozygosity levels (sample 12718, and 12719 from Sydney, M0271 from India, and M0208 from Fiji). Sample M0208 was not filtered out in the STACKS and BCFtools datasets as it did not show outlier observed heterozygosity or inbreeding coefficients.
- Retain SNPs that are only called once on the myna reference genome
- Retain SNPs with call rates > 0.8, and reproducibility > 0.95.
- Remove singletons and doubletons.
- Thinned dataset so that there is only one SNP per 100kb if they are on the same contig

# Appendix S3 Filtering methods and justification

## Biallelic SNPs

The variants identified by the BCFtools and STACKS pipelines were filtered to only retain biallelic SNPs using the *view* and *filter* command in BCFtools. After filtering, the resulting file is indexed using the *index* command in BCFtools. Indexing is performed following all filtering steps.

**bcftools view and filter**

bcftools view -m2 -M2 -v snps /path/to/variant_file | bcftools view -c1 | bcftools filter -e 'AC==0 || AC=AN' | bgzip -c > /path/to/vcf_filtered/variant_call_biallelic_snps.vcf.gz

bcftools index /path/to/vcf_filtered/variant_call_biallelic_snps.vcf.gz

# /path/to/variant_file is the path to the .bcf or .vcf.gz file containing all the variants as identified by the BCFtools and STACKS pipelines (section S2.2 and S2.3 above respectively)

# /path/to/vcf_filtered/variant_call_biallelic_snps.vcf.gz is the path to the output vcf.gz file containing only biallelic SNPs

# bcftools view -m2 -M2 -v snps filters the .bcf file for only biallelic SNPs

# bcftools view -c1 removes monomorphic 0/0 loci. This part is actually redundant as this is covered in the next section of the code

# bcftools filter -e 'AC==0 || AC=AN' filters out all monomorphic loci for reference (AC==0) and alternative genotype (AC=AN).

# bgzip -c compresses the output VCF from bcftools filter to a vcf.gz file. Note that this was used over the -Oz -o flag in bcftools because -Oz -o flag requires the input file to be indexed first.

# bcftools index indexed the resulting vcf.gz file which is required for further downstream processes.

## Read depths and genotype quality

Generally, minimum read depth filtering will remove false positive genotype calls, while maximum read depth cutoffs will remove genotype calls that may have potentially been mapped onto paralogs or repetitive parts of the genome.

The threshold for the read depths for the STACKS and BCFtools dataset were based on the distribution of depths of well-defined SNPs. Well-defined SNPs are based on the datasets that were filtered to only retain SNPs with GQ ≥ 30 (and QUAL ≥ 30 for BCFtools) with no missing data (genotyped in all individuals) and the distribution of the read depths were determined in each dataset. Whole number thresholds were guided by the 95-percentile centered around the median: minimum depth = 15, and maximum depth = 100 for the NZ and IND datasets and 125 for the ALL dataset. Figure S3.1-3 show the DP distribution for the ALL, NZ, and IND BCFtools datasets respectively. A similar distribution is observed for the STACKS dataset (results not shown). Note that DArT SNP dataset does not have genotype read depth information.

VCFtools was used to recode genotype calls to NA where genotype quality scores (GQ) were < 30 or where the coverage fell outside the chosen read depth cut off point of minimum and maximum read depth thresholds. The BCFtools dataset was also filtered to only retain SNPs with SNP quality scores (QUAL) ≥ 30. After some genotype calls were recoded to NA by VCFtools, the *filter* command in BCFtools was also used to remove any monomorphic SNPs.

vcftools --gzvcf ${inputa} --remove-indels --minQ 30 --minGQ 30 --minDP 15 --maxDP ${maxDP} --recode --recode-INFO-all --stdout | bcftools filter -e 'AC==0 || AC=AN' -Oz -o ${outputa}

# ${inputa} is the input vcf.gz file with only biallelic SNPs from the biallelic SNP filter

# ${maxDP} is the maximum depth cutoff point. This is 100 for the IND and NZ dataset, and 125 for the entire/ALL dataset

# ${outputa} is the output vcf.gz file from this filter.

# NOTE: --minQ 30 is only used when working with BCFtools dataset as the STACKS datasets do not have SNP quality scores


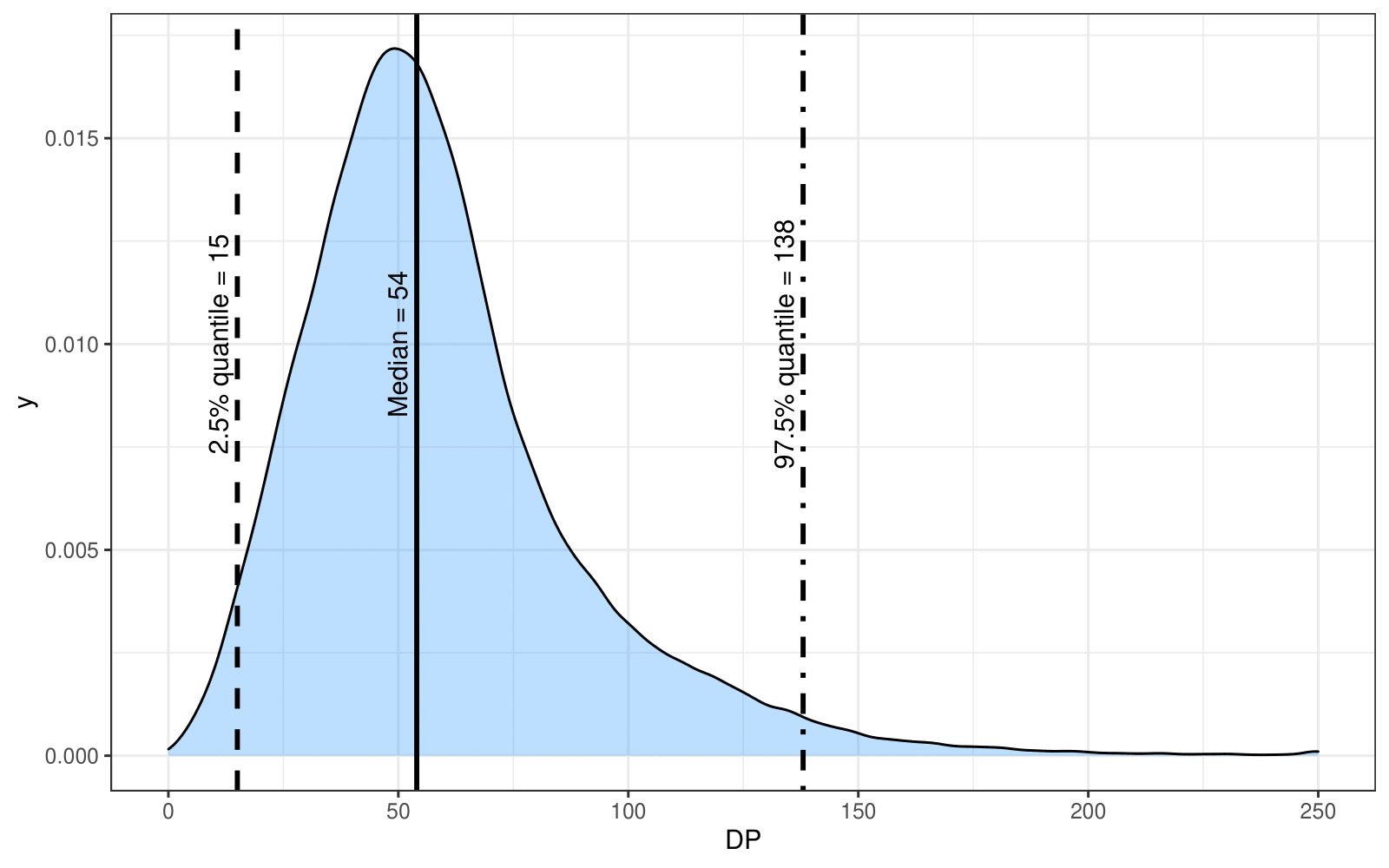


Figure S3.1 Read depth (DP) distribution in BCFtools ALL dataset after high quality filters (QUAL ≥ 30, GQ ≥ 30, and genotype present in all individuals. The median is 54 with a 95^th^ percentile range of 15-138.


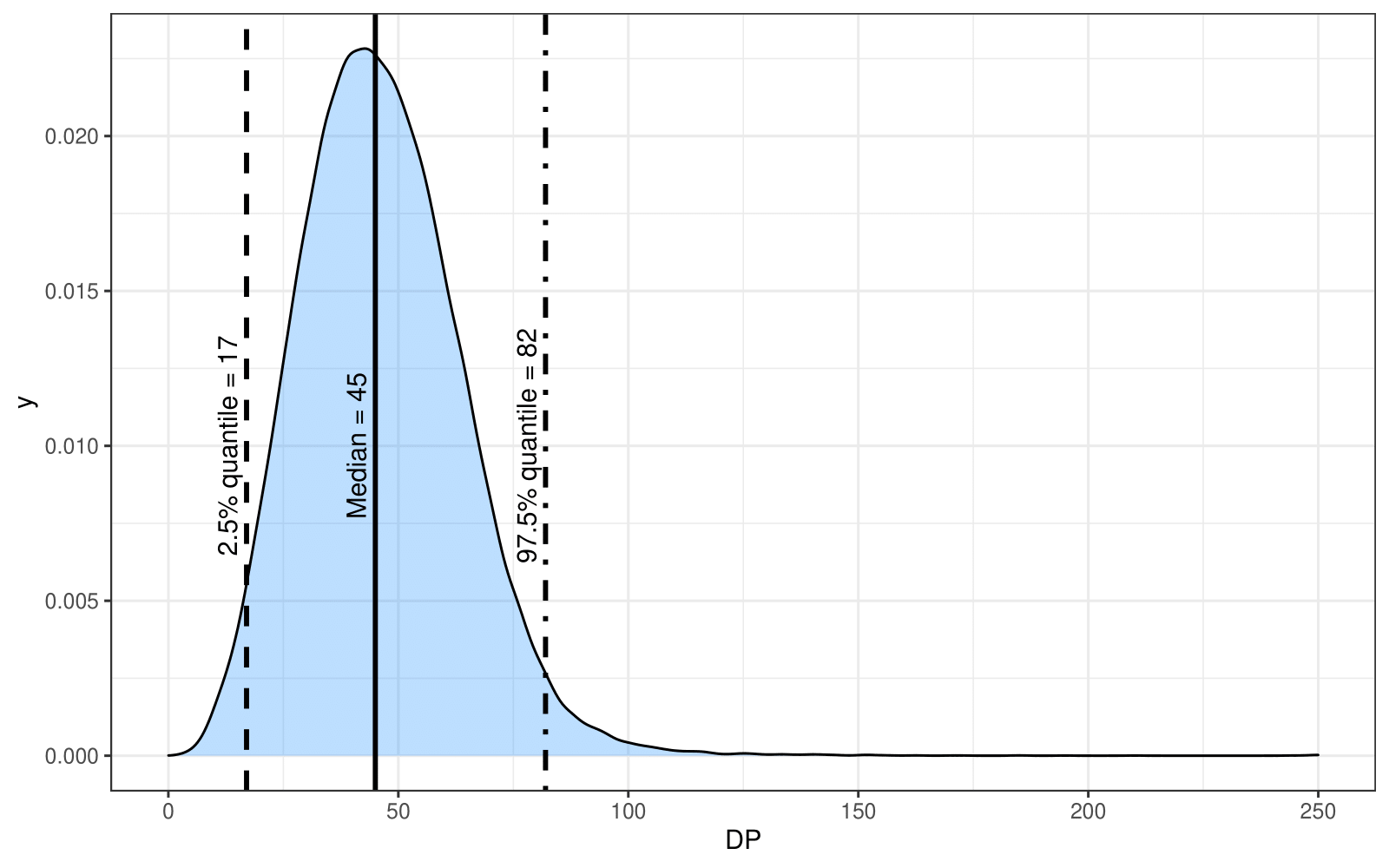


Figure S3.2 Read depth (DP) distribution in BCFtools NZ dataset after high quality filters (QUAL ≥ 30, GQ ≥ 30, and genotype present in all individuals. The median is 45 with a 95^th^ percentile range of 17-82.


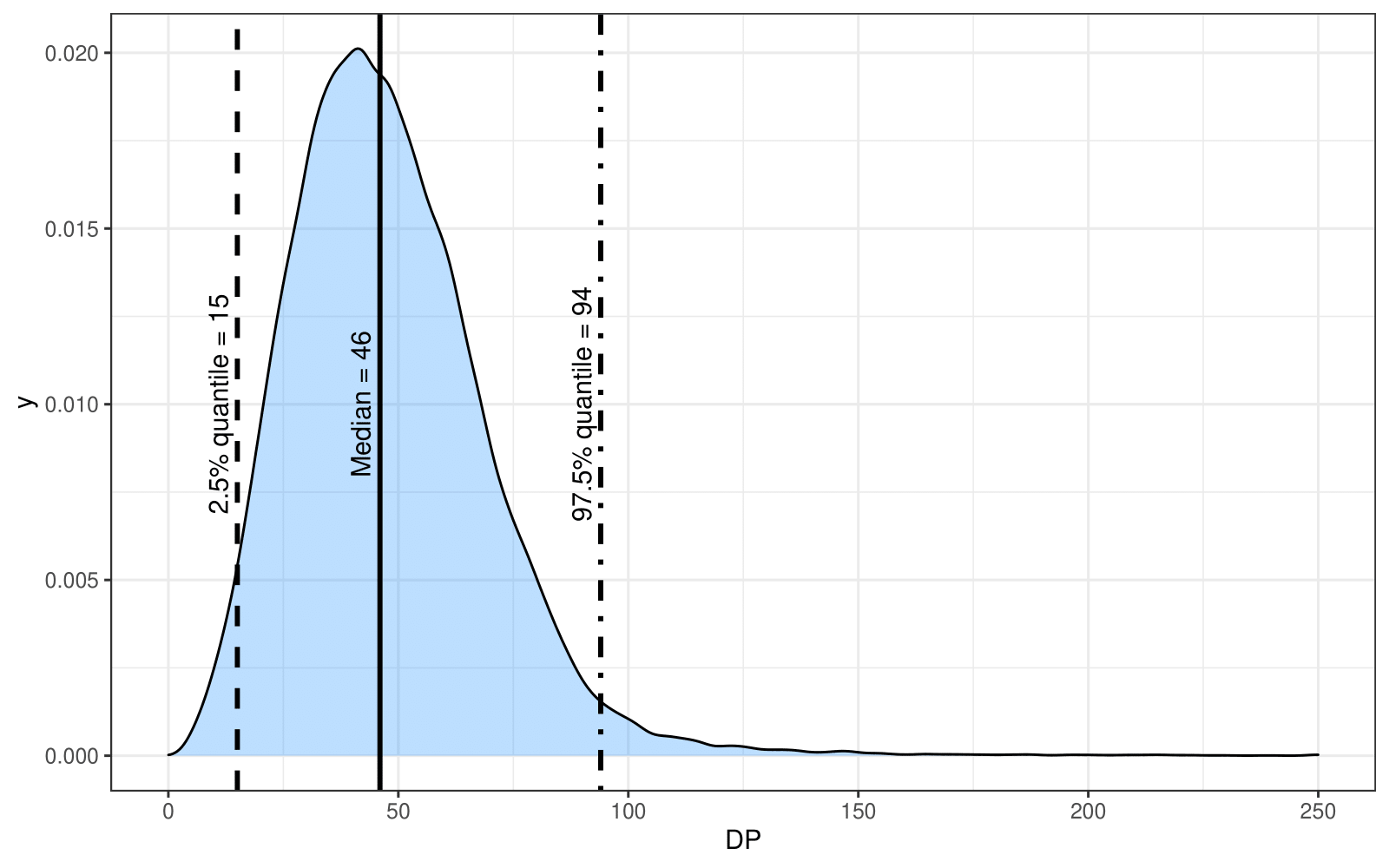


Figure S3.3 Read depth (DP) distribution in BCFtools IND dataset after high quality filters (QUAL ≥ 30, GQ ≥ 30, and genotype present in all individuals. The median is 46 with a 95^th^ percentile range of 15-94.

## Admixed Australian samples and replicates

Admixed Australian samples were removed from the dataset as admixed individuals are known to affect some downstream analysis. We also remove replicates at random prior to further SNP filtering. Both these steps were executed using the same command, except for the input text file containing the list of samples to be removed (${removesampletxt} in the command below):

bcftools view -S ^${removesampletxt} --force-samples ${inputa} | bcftools filter -e 'AC==0 || AC=AN' -Oz -o ${outputa}

# ${removesampletxt} is the path to the .txt file containing the sample ID(s) of the sample(s) to be removed from the dataset. This could be the list of admixed Australian samples or the list of replicates.

# ${inputa} is the resulting vcf.gz file from section S3.2 above.

# ${outputa} is the output vcf.gz file after the removal of the samples in ${removesampletxt}.

## Inbreeding coefficient (*F_IS_*)

*F_IS_* is the proportion of the variance in the subpopulation contained in an individual. A high positive value indicates some levels of inbreeding, and a high negative value indicates heterozygote excess (outbreeding, or potential sample mixing). Figure S3.4 shows the *F_IS_* for the BCFtools ALL dataset as calculated by the vcftools function with the --het option in VCFtools, showing the three anomalous samples (sample 12718, and 12719 from Sydney, and M0271 from India) with *F_IS_* < -0.25. *F_IS_* outliers were also observed in the other datasets in a similar manner (IND dataset, and STACKS dataset, results not shown). In the DART dataset, the *F_IS_* outliers were also observed with observed heterozygosity (H_o_), but also including sample M0208 from Fiji (results not shown).

The anomalous samples were removed using the *view* command in BCFtools. This was only executed in the ALL and the IND dataset and was executed alongside the SNP missingness filter (see command in section S3.5).


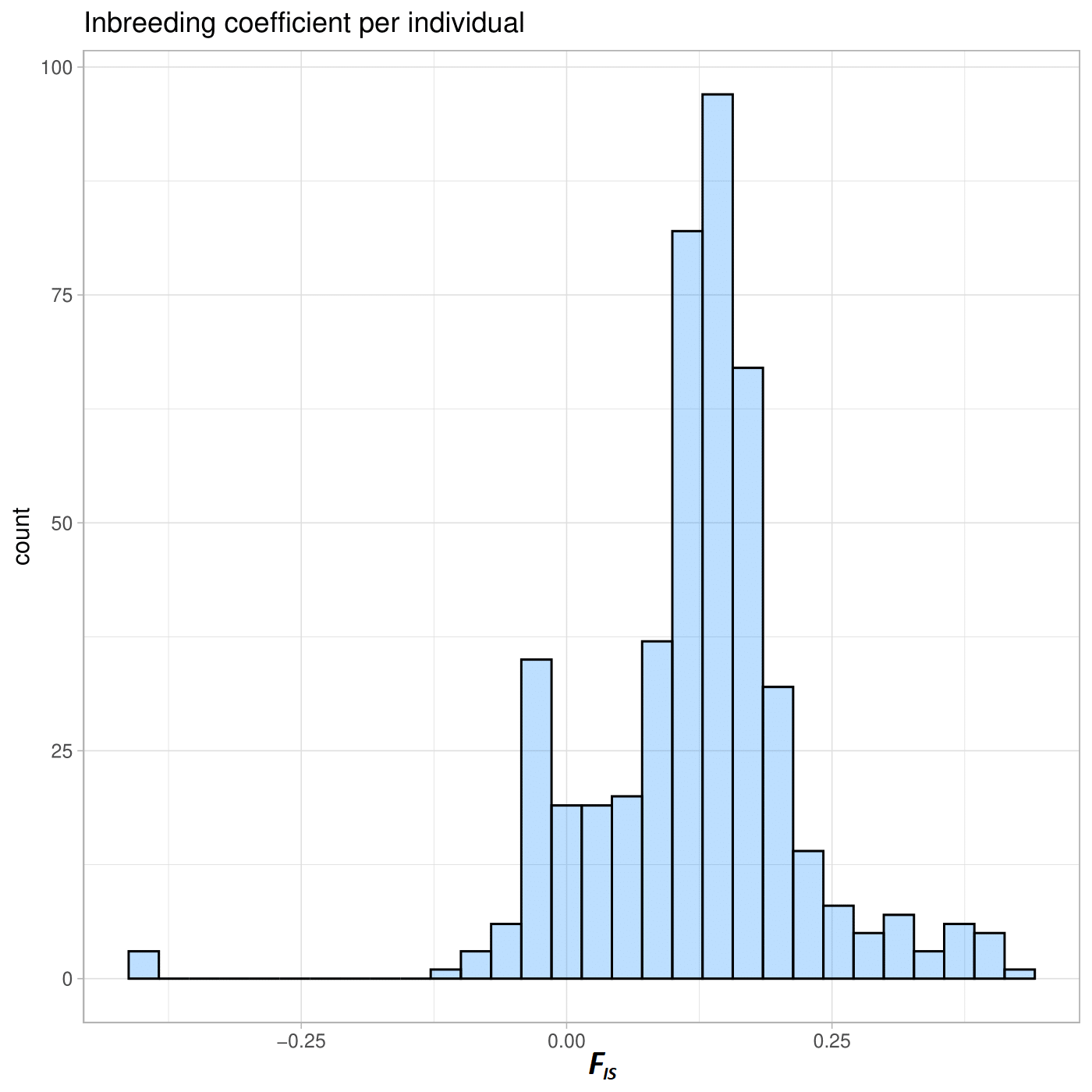


Figure S3.4 Individual F_IS_ as calculated in the BCFtools ALL dataset. 3 samples had anomalous F_IS_ values (F_IS_ < -0.25, heterozygote excess).

## SNP missingness

The locus data missingness thresholds were determined based on a balance between the number of SNPs retained and the data missingness. A histogram of the number of SNPs and the data missingness was produced and an arbitrary threshold was used. Figure S3.6 shows this for the ALL dataset as calculated by the vcftools function with the --missing-site option in VCFtools. Similar distributions are observed with the NZ and IND dataset, and also datasets from the STACKS and DART pipelines but not shown here.


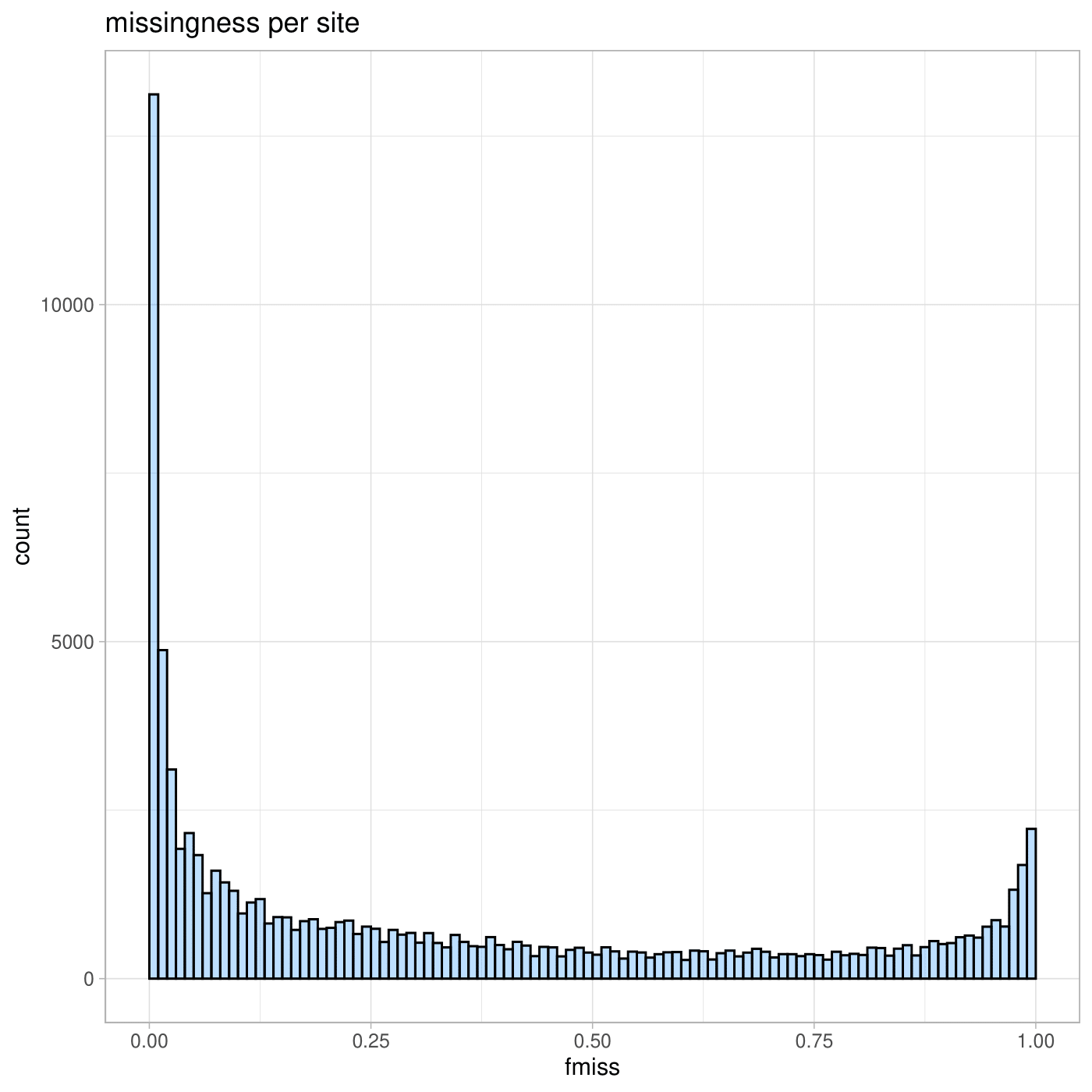


Figure S3.5 Histogram of number of SNPs and the data missingness in the ALL BCF dataset.

A threshold of 20% SNP data missingness was chosen and the *view* command in BCFtools was used to remove SNPs with more than 20% missingness. This was executed alongside the removal of the samples with anomalous inbreeding coefficients.

bcftools view -s ^12718,12719,M0271 ${inputa} | bcftools view -i 'F_MISSING<=0.2' | bcftools filter -e 'AC==0 || AC=AN' -Oz -o ${outputa}

# or

bcftools view -i 'F_MISSING<=0.2' ${inputa} | bcftools filter -e 'AC==0 || AC=AN' -Oz -o ${outputa}

# The first command excludes sample 12718, 12719 and M0271. The list of samples to exclude may change depending on the dataset being filtered.

# The second command does not exclude any samples and is used for the NZ dataset which has no samples with anomalous inbreeding coefficient.

# ${inputa} is the resulting vcf.gz file from section 3.3 above

# ${outputa} is the output vcf.gz file after the removal of samples with anomalous inbreeding coefficients, the removal of SNPs with more than 20% missingness, and the removal of monomorphic SNPs.

## Singletons and doubletons

For all downstream analyses, excluding the construction of the site-frequency-spectrum (SFS), demographic inference, and gene flow analysis (see Figure 2 in the main text and Figure S2.1), singletons and doubletons (SNPs only occurring in one sample) are removed from the dataset using the following VCFtools commands.

vcftools --gzvcf ${vcfa} --singletons --out ${singleton}

vcftools --gzvcf ${vcfa} --exclude-positions ${singleton}.singletons --recode --recode-INFO-all --stdout | bcftools annotate --set-id "%CHROM\_%POS\_%REF\_%ALT" -O z -o ${vcfb}

# The first command makes a list of singletons and doubletons

# The second command exclude singletons and doubletons from the VCF file using the list created from the first command and pipes this through bcftools annotate and setting the ID to the CHROM_POS_REF_ALT nomenclature before outputting to a vcf.gz file.

# ${vcfa} is the path to the resulting vcf.gz file from section 3.5 (after removing samples with anomalous inbreeding coefficients and after removing SNPs with more than 20 percent data missingness)

# ${singleton} is path to the output text file from the first command, containing a list of singletons and doubletons.

# ${vcfb} is the path to the output vcf.gz file after the removal of singletons and doubletons.

# Appendix S4 – Linkage disequilibrium analysis

Pairwise LD between all loci within 500kb on the same contig was calculated and plotted against distance in kb using the PopLDdecay program (Zhang *et al.*, 2019). This was performed prior to thinning the SNPs in the VCF files using the following command:

./PopLDdecay -InVCF ${vcfa} -OutStat ${outpath}

perl Plot_OnePop.pl -inFile ${outpath}.stat.gz -output ${outputPDF}

# The first command runs PopLDdecay and output a file containing all pairwise LD between all loci within 500kb on the same contig

# The second command makes the LD decay plots (e.g. Figure S4.1-3) based on the output from the first command using a perl script that is provided with PopLDdecay program

# ${vcfa} is the path to the vcf.gz file to calculate LD decay. The command was run with vcf.gz files before and after the removal of singletons and doubletons.

# ${outpath} is the path to the output file containing the pairwise LD between all loci within 500kb on the same contig. The resulting file often has the .stat.gz suffix appended to the path.

# ${outputPDF} is the path to the output PDF file containing the LD plot. The output PDF file often has the .pdf suffix appended to the path.

In summary, LD decays to near background level after 50kb. Background level differs depending on the dataset. LD decays slower in the NZ dataset (introduced populations) when compared to the IND dataset. A 100kb threshold was conservatively chosen to remove strong linkage disequilibrium in all datasets, retaining a good number of SNPs (about 5000-6000 per dataset). This was done using the following VCFtools command:

vcftools --gzvcf ${vcfa} --thin 100000 --recode --recode-INFO-all --stdout | bcftools filter -e 'AC==0 || AC=AN' -Oz -o ${vcfthin}

# ${vcfa} is the path to the vcf.gz file to thin. The command was run with vcf.gz files before and after the removal of singletons and doubletons.

# ${vcfthin} is the path to the resulting vcf.gz file after thinning. Note that BCFtools was used to ensure removal of monomorphic SNPs and compress the resulting VCF file.


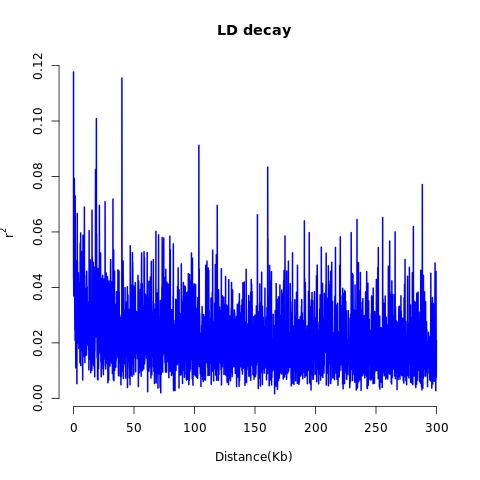


Figure S4.1 LD decay in ALL dataset. LD appears to level off at ~0.02-0.03.


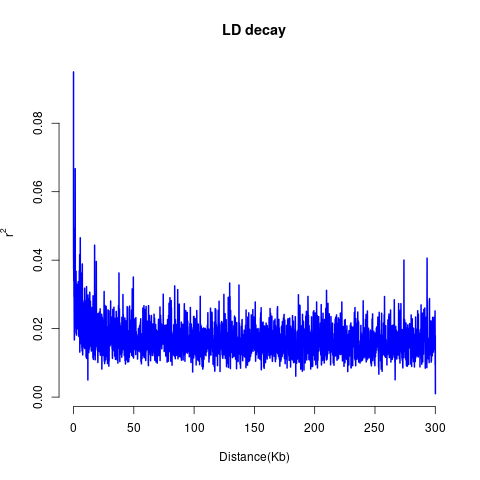


Figure S4.2 LD decay in IND dataset. LD appears to level off below 0.02.


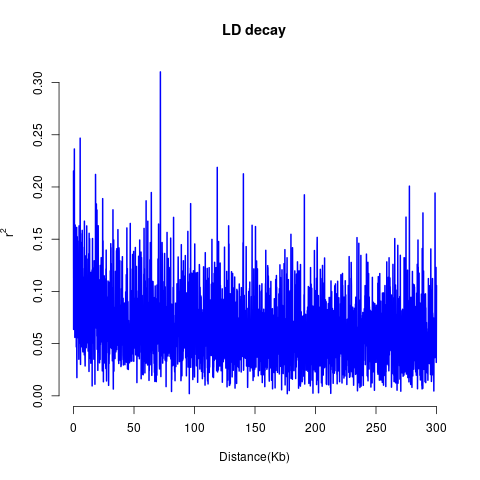


Figure S4.3 LD decay in NZ dataset. LD appears to level off between 0.05 and 0.1.

# Appendix S5 – Subsampling


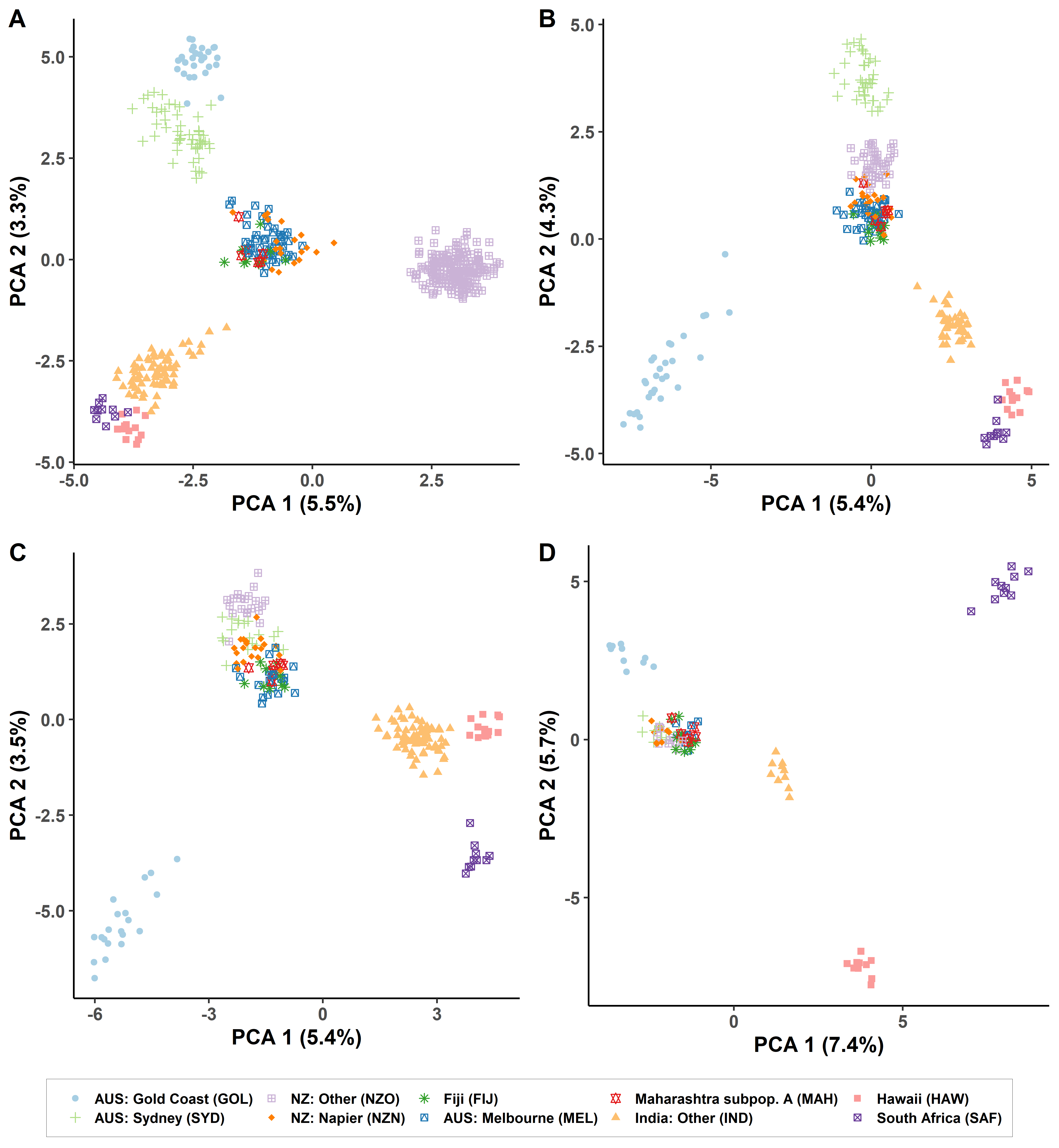


Figure S5.1 PCA 1 vs 2 on different subsamples of the dataset: A) no subsampling, B) populations subsampled to n ≤ 40, C) n ≤ 20 (same as Figure 5A in main text), and D) n ≤ 10. Samples are labelled based on popdef2, same as in Figure 5A in the main text.


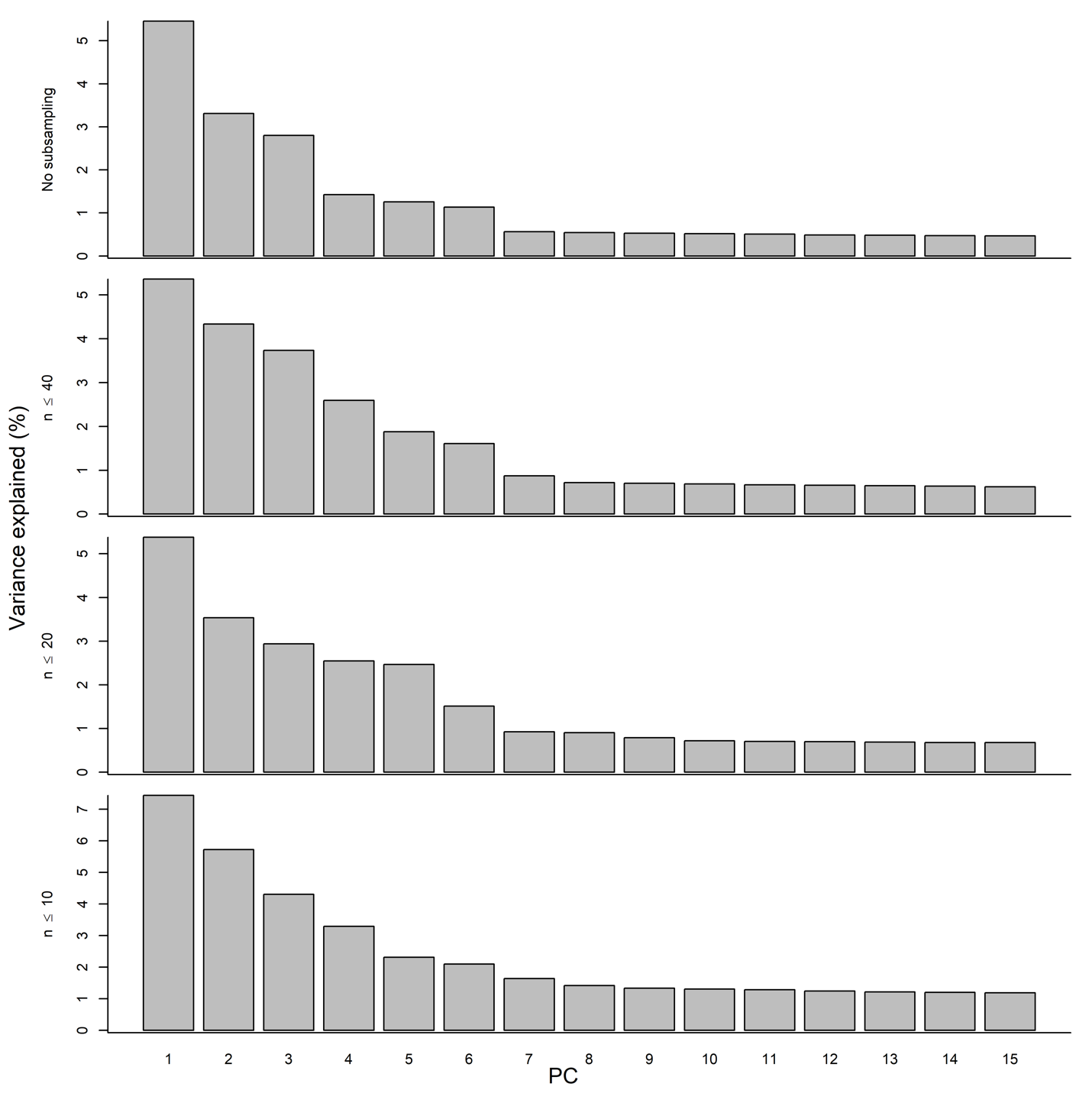


Figure S5.2 Scree plot of variance explained of the first 15 principal components from the PCA on different subsets of the ALL dataset

# Appendix S6 – Hardy Weinberg Equilibrium

Hardy Weinberg Equilibrium (HWE) tests were performed using the Fisher’s exact test using the ‘HardyWeinberg’ R package version 1.7.2 (Graffelman and Camarena, 2008; Graffelman, 2015). To avoid the Wahlund effect problem which can minimise the differences between populations (see ‘Out Across’ filtering approach in Pearman, Urban and Alexander (2022)), the HWE tests were performed on each population as defined by the popdef1 definition.

Figure S6.1 displays the p-value histogram of the HWE tests for the ALL dataset. The shape of the p-value distribution falls far from the expected shape (a uniform shape with a peak near 0), making it difficult for multiple testing correction. Similar shapes are also observed with HWE tests for the NZ and IND dataset (Figures not shown here).


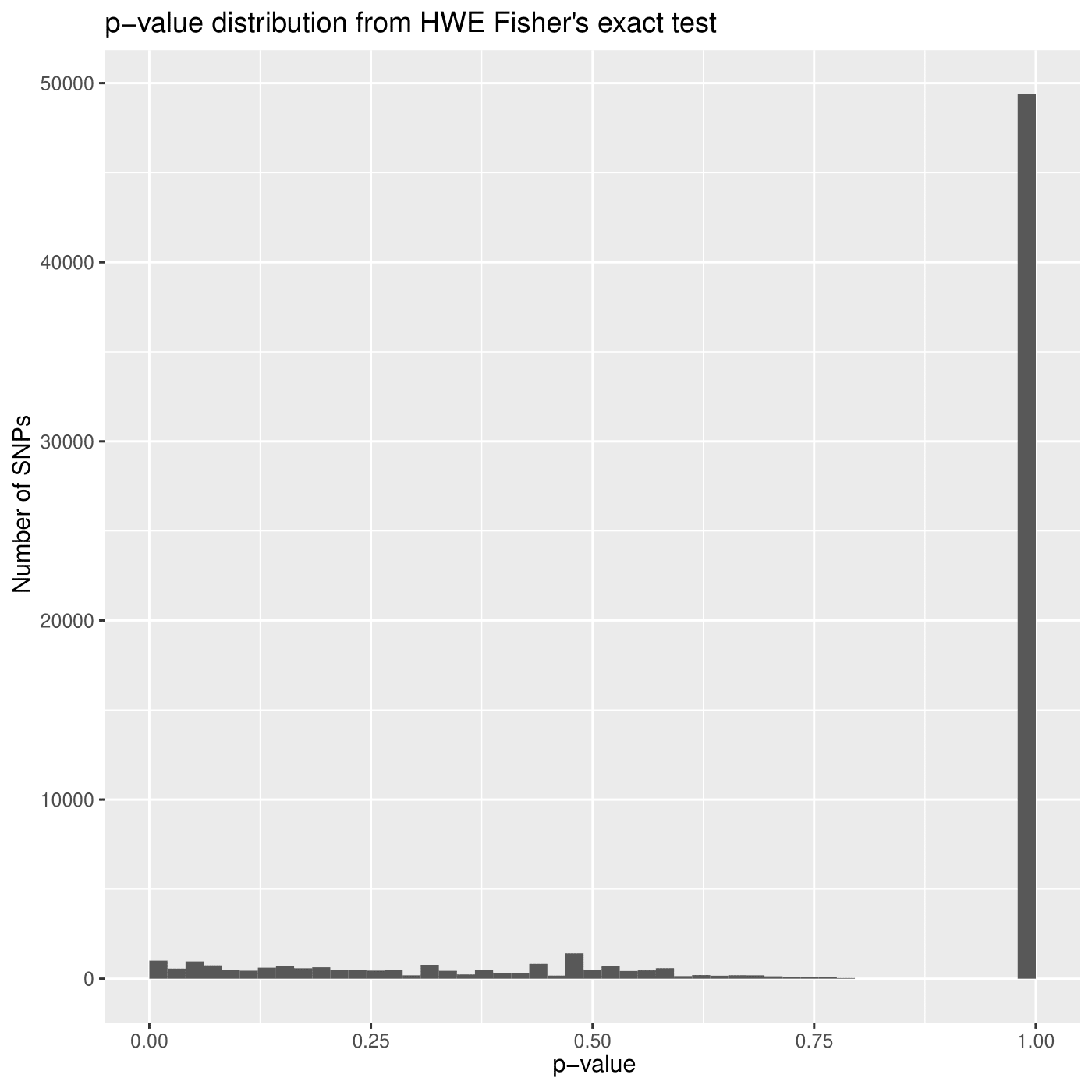


Figure S6.1 p-value distribution of the HWE test on the ALL dataset (populations defined based on popdef1 definition) using the Fisher's exact test

# Appendix S7 – Demographic inference and gene flow analysis

## fastsimcoal2

fastsimcoal2 was used to infer the demographic history for the New Zealand common mynas from the observed site frequency spectrum (SFS). Based on results from our population structure analyses (PCA, sNMF, population pairwise-*F_ST_*), we identified those populations most relevant to the establishment of mynas in New Zealand, and hence included Maharashtra subpopulation A, Melbourne (ROM) (clear clustering with NZ samples) along with Napier (ROM), NZ: Other (ROM). The ROM samples were used rather than contemporary samples from New Zealand and Australia as they were collected at a similar time (1970s-1980s) and to shorten the coalescent times (Indian samples are also from ROM).

As fastsimcoal2 is based on the SFS, sensitive to missing data, and uses unlinked SNPs, the data used were filtered slightly differently from other datasets (see Figure 2 and Figure S2.1). We did not filter out singletons and doubletons prior to thinning the SNPs because filters based on minor allele frequency (MAF) or singletons/doubletons introduced SNP ascertainment biases and affect the SFS of different populations differently depending on the samples available. We also did not filter for HWE. Due to uneven sampling and missing data, we down-projected the VCFs to SFS using easySFS.py (<https://github.com/isaacovercast/easySFS>), with n = 6,8,10,20 for Maharashtra subpopulation A, Melbourne (ROM), Napier (ROM), and NZ: Other, respectively. The numbers were chosen to make sampling more even and to maximize the number of segregating sites.

We tested three scenarios, involving the origin of NZ: Other (Figure S7.1). In scenario 1, 2, and 3, the NZ: Other population was founded by Napier, Melbourne, and Maharashtra subpopulation A, respectively. The fastsimcoal2 simulations assume discrete generations. To reduce the number of parameters estimates and avoid over-parametrization, the divergence time (in generations since data collection) at the divergence between the Melbourne and Maharashtra subpopulation A populations, and between Napier and Melbourne were fixed at 31 and 27 generations since 1984. These were approximated from a generation time of four years, and the introduction time to Melbourne in 1862 (Long, 1981) ((1984 – 1862)/4 = 30.5), and the introduction time to Napier in 1876 (date from Beesley (unpublished), (1984 – 1876)/4 = 27)). The average age of adult mynas in Hawke’s Bay, New Zealand were found to be approximately four years old (Wilson, 1973). Maharashtra subpopulation A samples were collected in 1975, and assumed to be collected at generation 2 ((1984 – 1975)/4 = 2.25). Other ROM samples were collected in 1983/4 and were assumed to be collected at generation 0. A mutation rate estimated from another passerine species, collared flycatcher (Ficedula albicollis), was used (4.6e-9 mutations per site per generation) (Smeds, Qvarnström and Ellegren, 2016).

For each scenario, we performed 100 independent model runs (40 expectation/conditional maximization [ECM] cycles, 200,000 simulations per run) and chose the run with the highest likelihood as the best estimate of the demographic parameters for each scenario. The likelihood distributions for each scenario were estimated by running fastsimcoal2 using the best estimate of the demographic parameters for each scenario 100 times (1,000,000 simulations per run).

The likelihood distributions for each of the scenarios overlap and suggest that the models do not differ significantly (Figure S7.2).


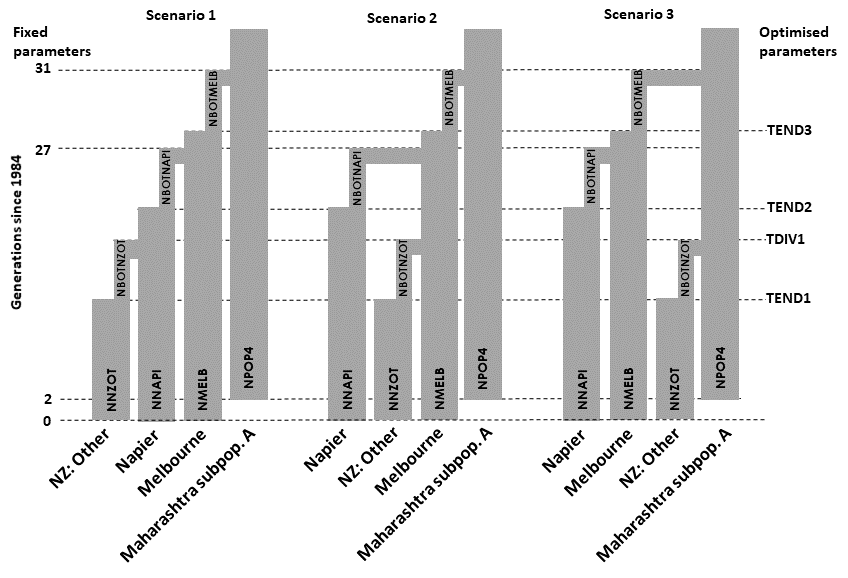


Figure S7.1 Three scenarios involving the origin of the NZ: Other population. All populations were ROM samples. tested using fastsimcoal2. The priors for the diverging time of NZ: Other from the source population (TDIV1) vary depending on the scenario in order to avoid TDIV1 being older than the population in which it diverged from (see Table S7.1 for more details). Note that that population size was assumed constant and only resized during TEND1, TEND2 and TEND3. This simplifies the model and avoid parametisation of additional complex parameters such as population growth rate.

Table S7.1 Table summarizing the priors used for the parameters optimised in fastsimcoal2. All priors follow a uniform distribution and is the same in all scenarios, except for TDIV1 which are different in different scenarios to avoid TDIV1 being older than the population in which it diverged from. The minimum value for TDIV1 in scenario 2 and 3 is 27, which assumes that the mynas that found the NZ: Other population diverged from the founding population at the same or earlier time than the mynas that found the Napier population. The choice of this number is influenced by the historical records of myna introduction in New Zealand – most introductions of mynas to New Zealand date to 1870-1880. Each parameter is also depicted in Figure S7.1 which visualises the three demographic scenarios. Number of individuals are in haploid individuals and have to be divided by 2 to represent diploid individuals. TMRCA = time to most common ancestor. Time units are in generations before 1984.

| **Parameters** | **Description** | **Minimum** | | | **Maximum** | | |
| --- | --- | --- | --- | --- | --- | --- | --- |
|  |  | **Scenario 1** | **Scenario 2** | **Scenario 3** | **Scenario 1** | **Scenario 2** | **Scenario 3** |
| NPOP4 | Maharashtra subpopulation A haploid population size | 2000 | | | 200000 | | |
| NMELB | Melbourne haploid population size | 20000 | | | 200000 | | |
| NNAPI | Napier haploid population size | 2000 | | | 200000 | | |
| NNZOT | NZ: Other haploid population size | 2000 | | | 200000 | | |
| NBOTMELB | Melbourne introduction bottleneck haploid population size | 2 | | | 2000 | | |
| NBOTNAPI | Napier introduction bottleneck haploid population size | 2 | | | 2000 | | |
| NBOTNZOT | NZ: Other introduction bottleneck haploid population size | 2 | | | 2000 | | |
| TDIV1 | TMRCA between NZ: Other and founding population | 16 | 27 | 27 | 26 | 30 | 33 |
| TEND1 | Time of population expansion after bottleneck in NZ: Other | 1 | | | TDIV1 | | |
| TEND2 | Time of population expansion after bottleneck in Napier population | 1 | | | 27 | | |
| TEND3 | Time of population expansion after bottleneck in Melbourne population | 1 | | | 31 | | |

Table S7.2 Best parameter estimate based on three different scenarios involving the origin of NZ: Other. See Figure S7.1 and Table S7.1 for explanation of the scenarios and the parameters.

| **Parameters** | **Scenario 1** | **Scenario 2** | **Scenario 3** |
| --- | --- | --- | --- |
| NPOP4 | 172008 | 97321 | 152464 |
| NMELB | 122777 | 153414 | 112383 |
| NNAPI | 78270 | 150841 | 76588 |
| NNZOT | 146134 | 173296 | 52223 |
| NBOTMELB | 1840 | 1779 | 1179 |
| NBOTNAPI | 642 | 775 | 755 |
| NBOTNZOT | 23 | 243 | 301 |
| TDIV1 | 26 | 30 | 30 |
| TEND2 | 6 | 3 | 5 |
| TEND3 | 18 | 20 | 25 |
| TEND1 | 24 | 8 | 3 |
| MaxEstLhood | -14263.7 | -14261.7 | -14260.4 |
| MaxObsLhood | -14076.8 | -14076.8 | -14076.8 |

Parameters estimated from fastsimcoal2 in all three scenarios showed the general trend that the NZ: Other population was the most bottlenecked, followed by NZ: Napier and Melbourne However, the exact numbers will have to be treated with caution as there are a lot of uncertainties involved with the model parametrization. For example, the assumption of discrete generation time is most likely voided and the constant population size during, before and after the population/genetic bottlenecks are not realistic.

The likelihood distributions for each scenario were estimated by running fastsimcoal2 using the best estimate of the demographic parameters (Table S7.2) for each scenario 100 times (1,000,000 simulations per run) and the overlap in distribution indicates that there are no significant differences between the fit of the different models (Meier *et al.*, 2017).


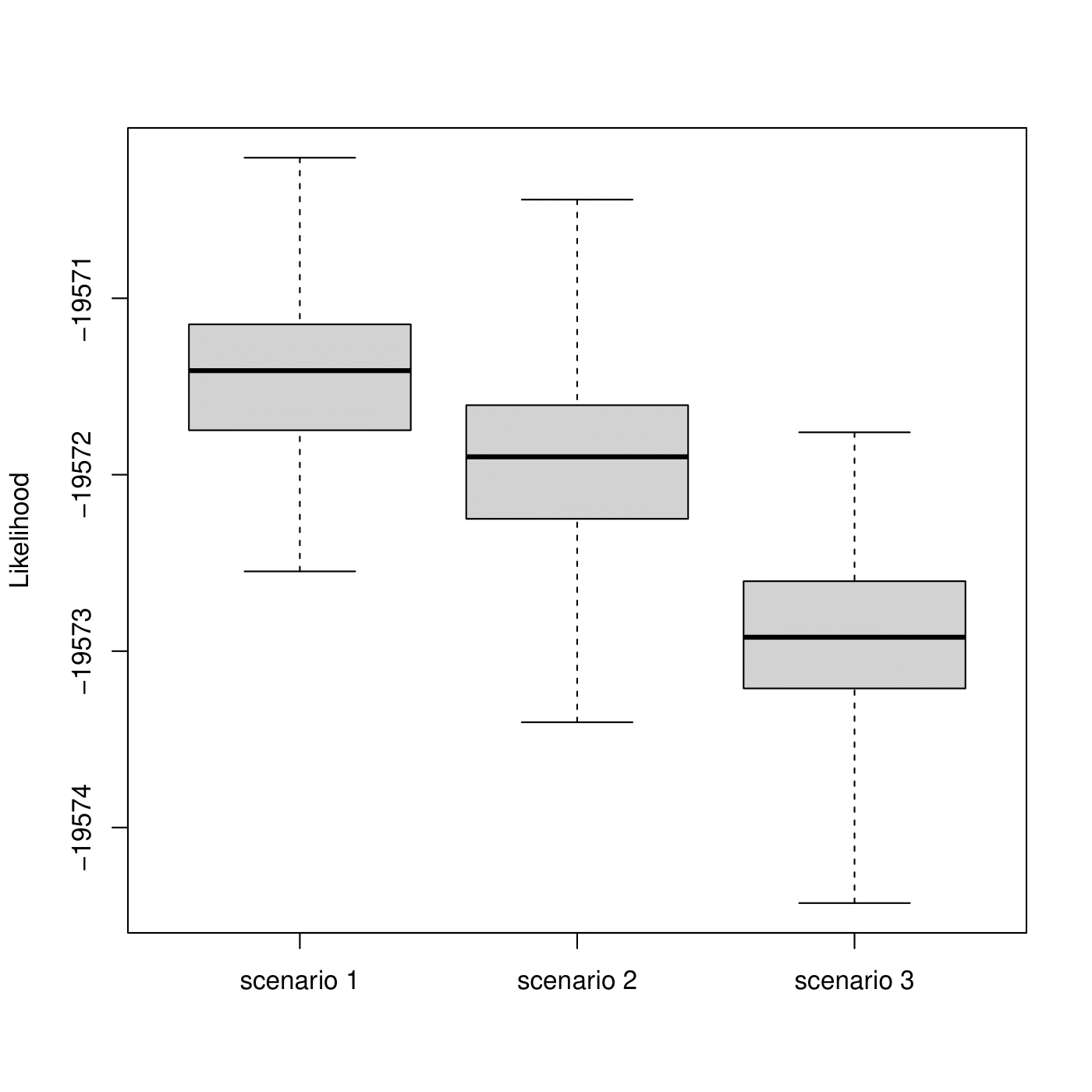


Figure S7.2 Loglikelihood distribution of each scenario involving the origin of NZ: Other. The distribution was determined from 100 runs (1,000,000 simulations per run) using the best parameter estimates. scenario 1 is NZ: Other originating from Napier, scenario2 is NZ: Other originating from Melbourne, and scenario3 is NZ: Other originating from the Maharashtra subpopulation A.

## BA3-SNPs

Contemporary migration patterns were estimated using BA3-SNPs version 3.0.4 (Wilson and Rannala, 2003; Mussmann *et al.*, 2019) among the New Zealand populations (n ≥ 20) within NZ: Other, and between the Napier and Leigh population (representative of the NZ: Other population). For similar reasons for fastsimcoal2 simulations, the dataset for BA3-SNPs were filtered in the same way (see Figure 2 and Figure S2.1), and populations were subsampled to n = 20 to avoid sample-size biases. BA3-SNPs also do not assume HWE. BA3-SNPs-autotune (Mussmann *et al.*, 2019) was used to tune mixing parameters for BA3-SNPs with default settings (1,000 burn-in and 10,000 generations). BA3-SNPs was run for 10,000,000, 1,000,000 burn-in, and a sampling frequency of 1,000.

BA3-SNPs was unable to detect any gene flow between any of the populations. This is likely due to our dataset, and population structure and history having voided optimal conditions for the analysis. BayesAss (the original software underlying BA3-SNPs) have been shown to prefer the following settings (see Faubet, Waples and Gaggiotti (2007) and Meirmans (2014) for evaluation on empirical and simulation data):

- Sufficient samples (n ≥ 20)
- Sufficient loci (≥ 15 markers)
- Equilibrium migration rate (can be asymmetric)
- Low migration rate (< 0.3)
- Some level of population structure (population pairwise-*F_ST_* ≥ 0.05; Faubet, Waples and Gaggiotti (2007))

This, however, depends on the dataset. It is likely that the populations in our analysis are not differentiated enough for BA3-SNPs to be able to deduce the gene flow. The populations in our analysis have also been a result of a very recent population expansion and shares a recent population history, meaning that identifying population specific alleles will be very difficult.

# Appendix S8 – Population structure figures

## NZ dataset


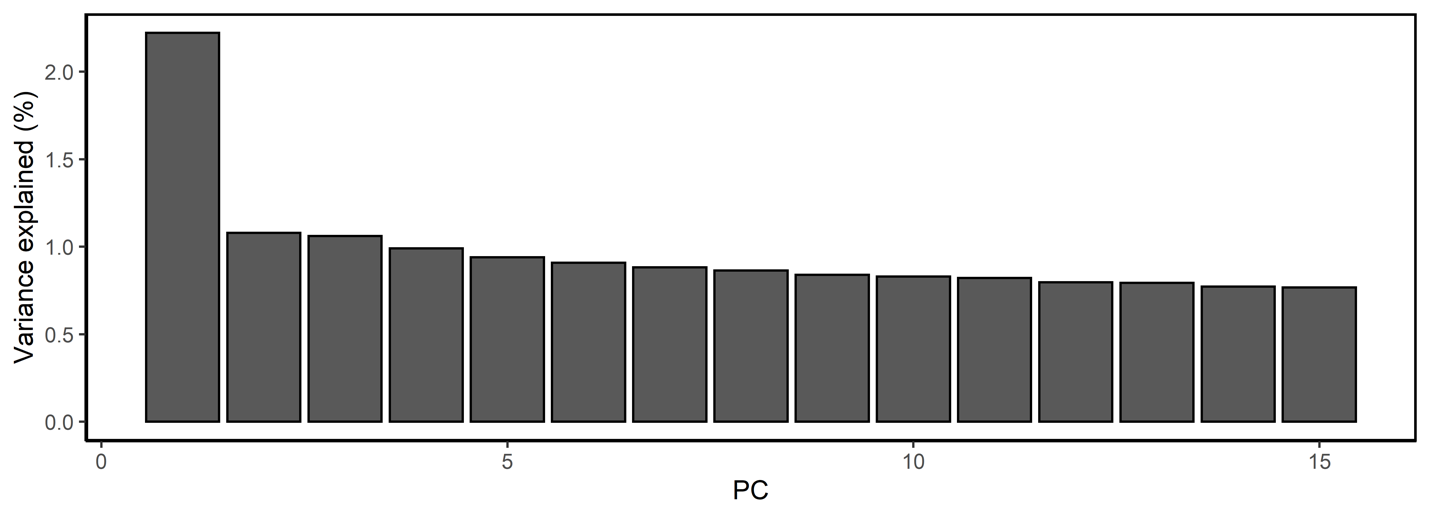


Figure S8.1 Scree plot of variance explained of the first 15 principal components from the PCA on the NZ dataset


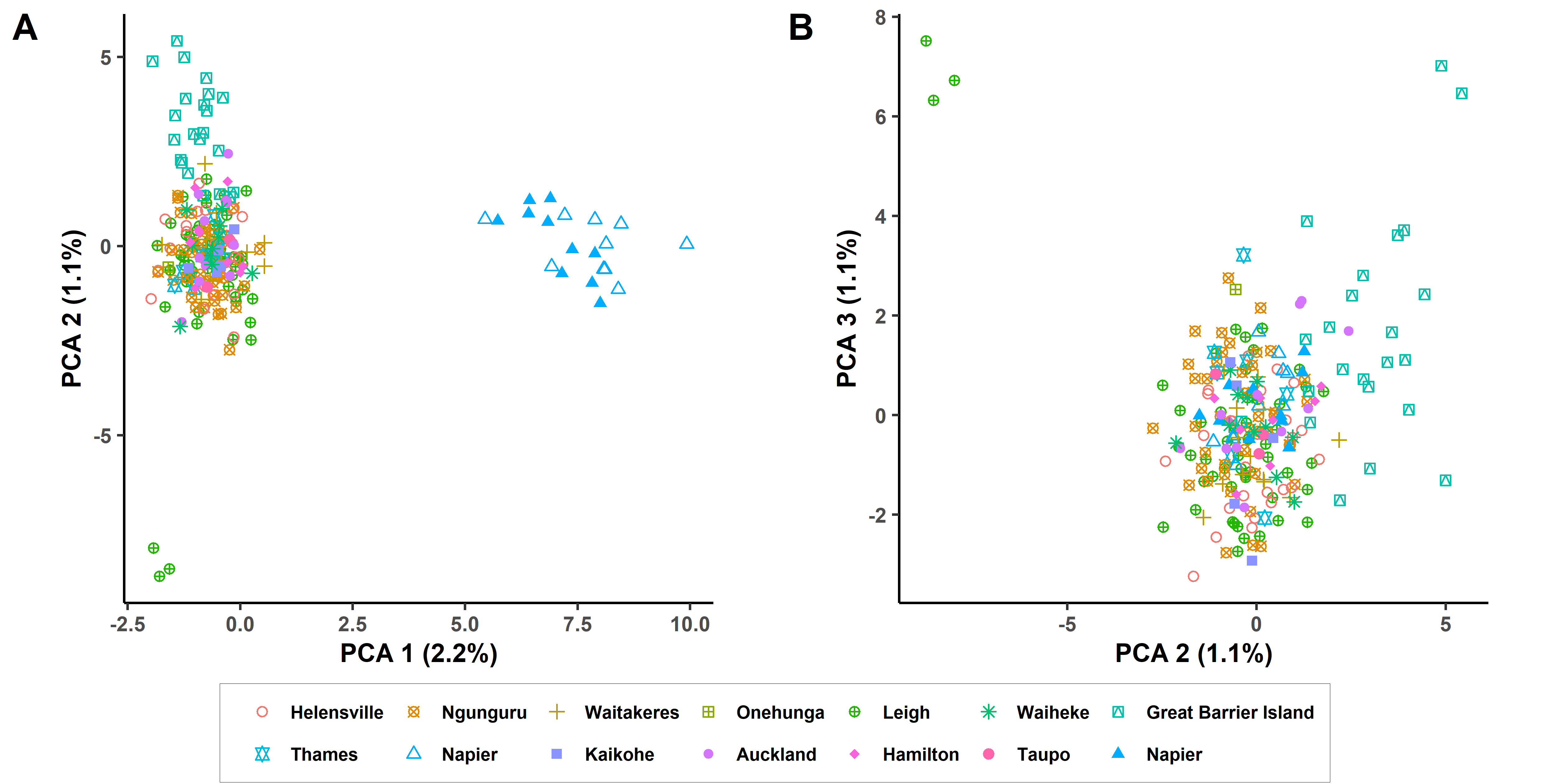


Figure S8.2 PCA plots of the NZ dataset. A) PCA1 vs PCA2, and B) PCA2 vs PCA3 of the NZ dataset. Filled shapes represent data from ROM.


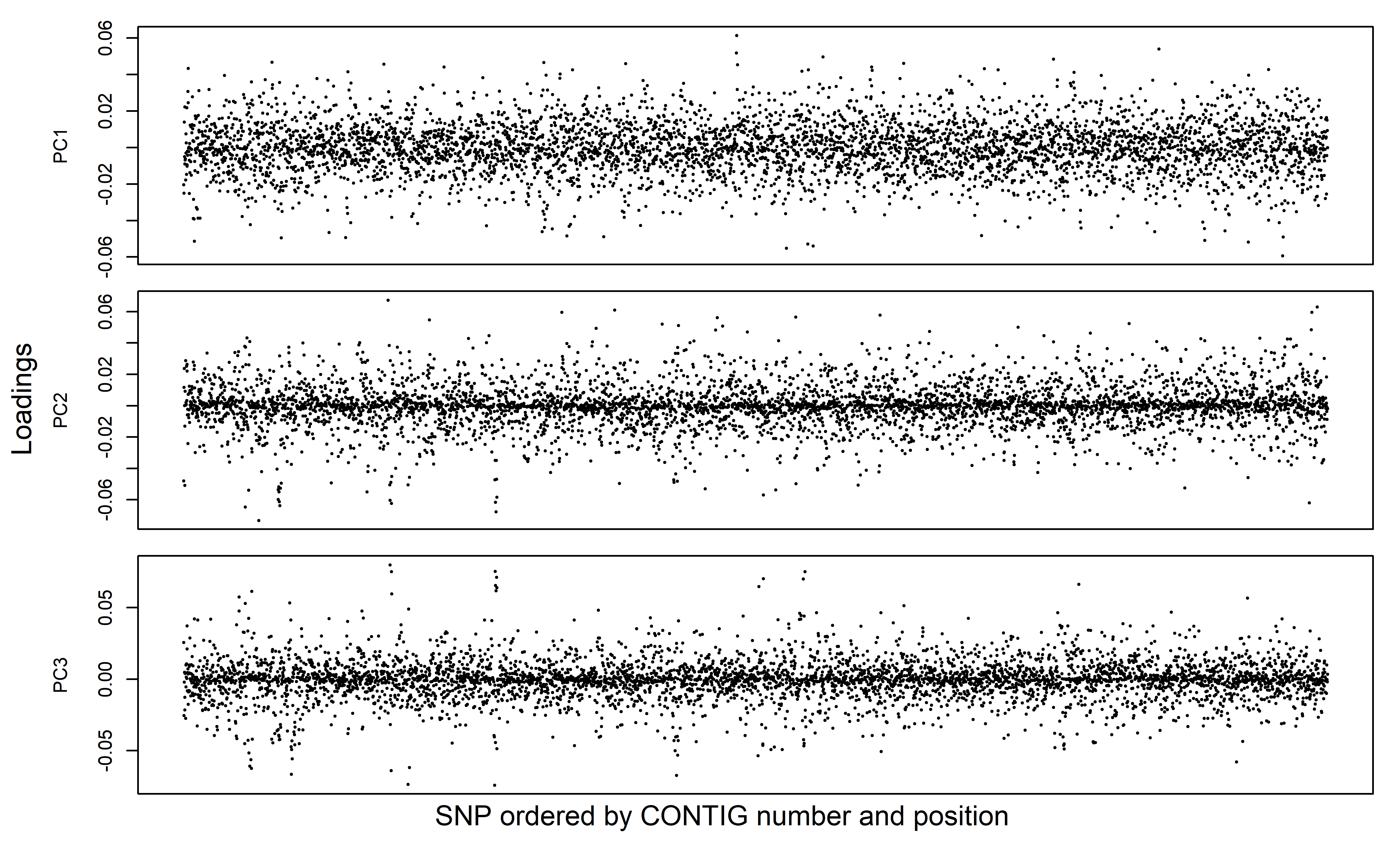


Figure S8.3 Loadings of PC1-3 for PCA on the NZ dataset.


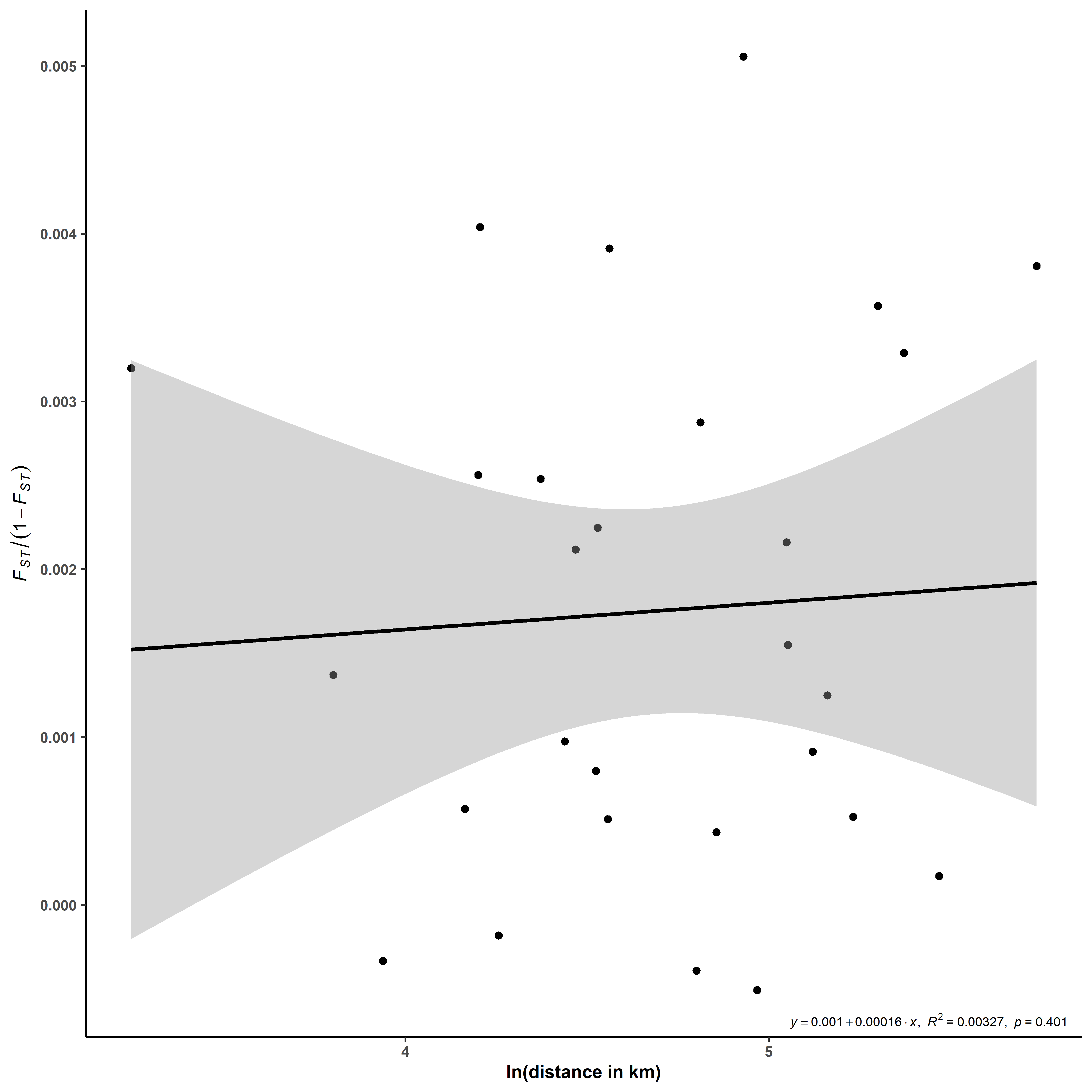


Figure S8.4 Mantel test on natural log transformed geographical distance (km) vs transformed population pairwise-F_ST_ (F_ST_/(1−F_ST_)) in the populations as defined by popdef1 from NZ: Other, excluding Great Barrier Island.


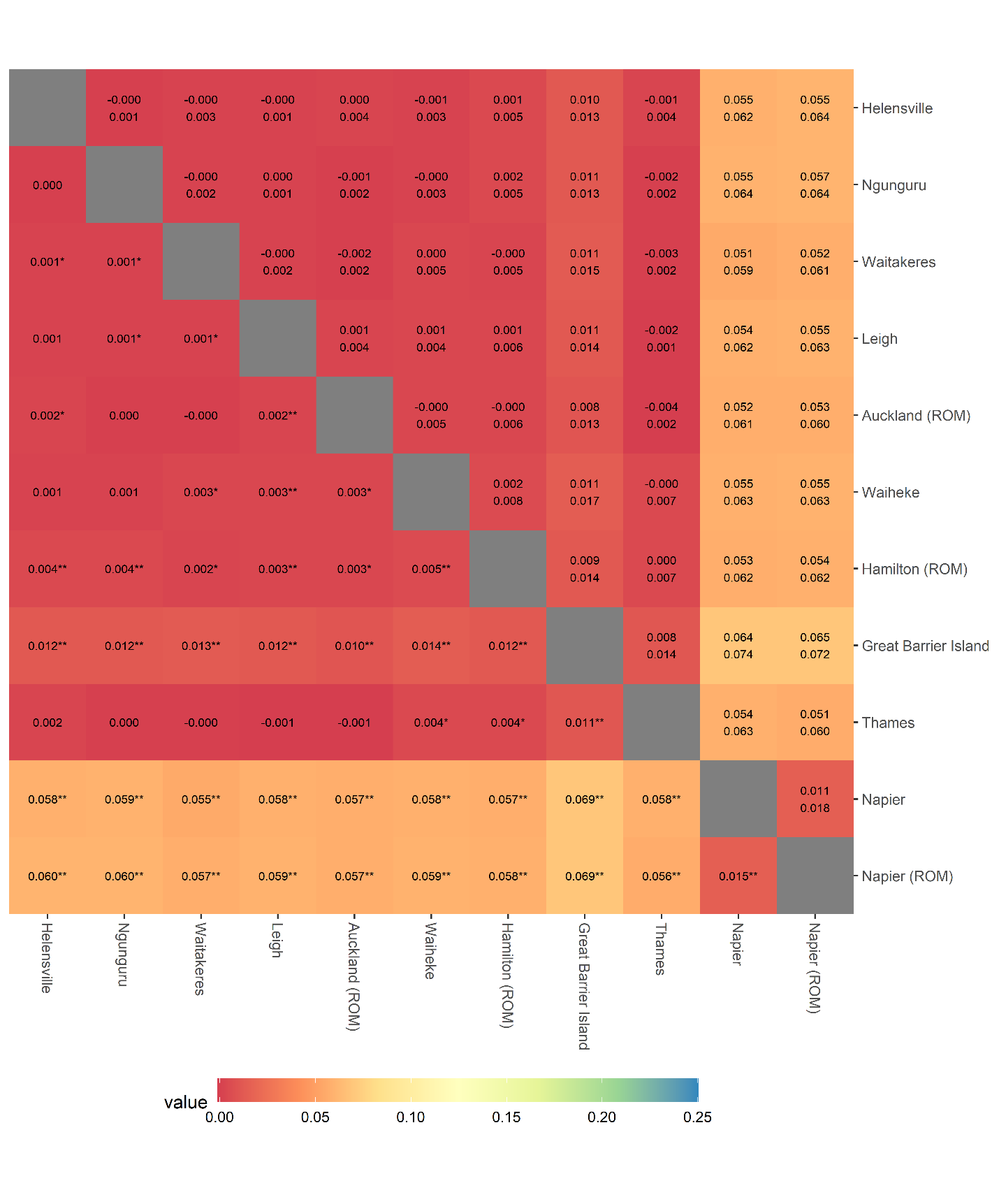


Figure S8.5 Heat map of population pairwise-F_ST_ comparisons (Weir and Cockerham, 1984) between all populations in the NZ dataset based on 100 bootstrap replicates. Mean pairwise-F_ST_ are shown on the lower triangle, and the 95% lower and upper confidence interval is shown on the upper triangle. * = FST values with p-values < 0.05, ** = FST values with p-values < 0.01. Note that the colour scale differs somewhat from Figure 6 in the main text.


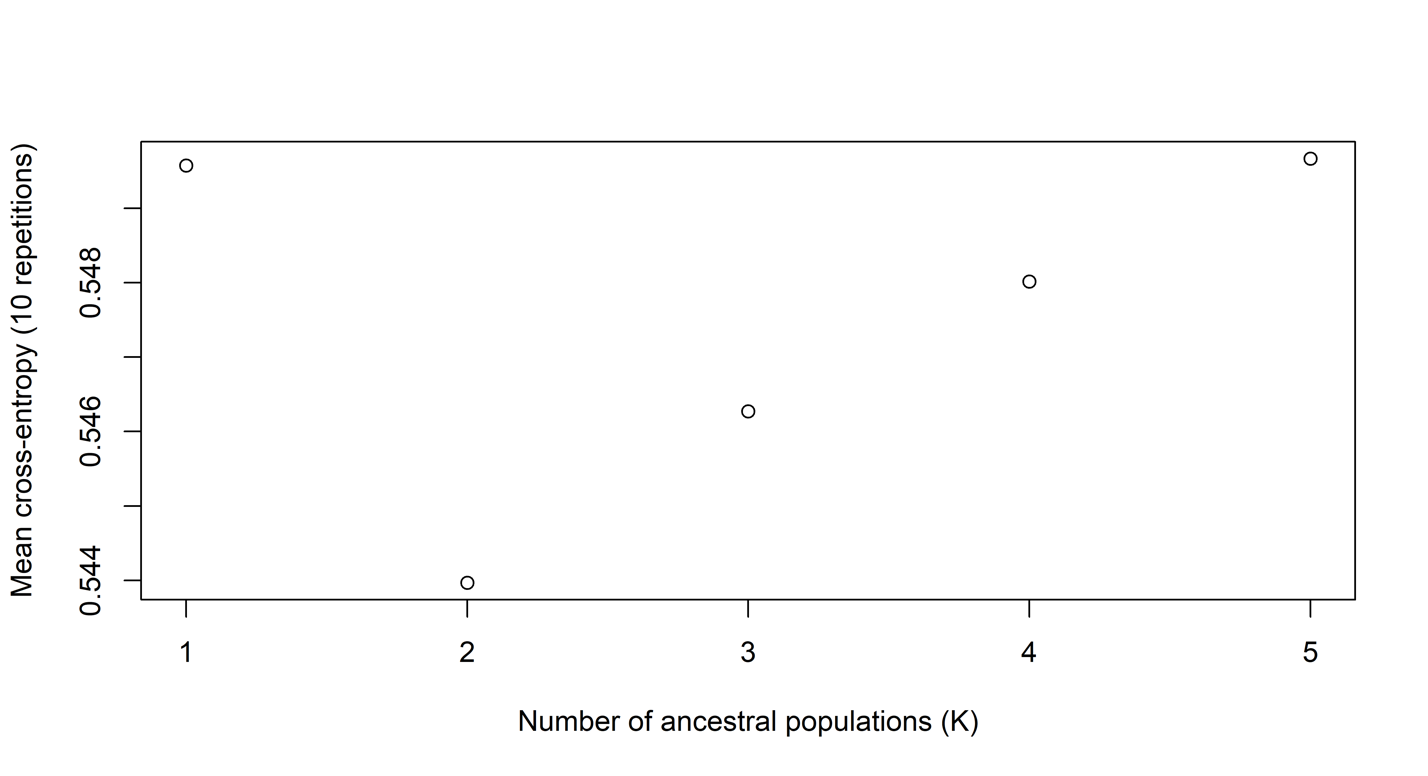


Figure S8.6 Mean cross-entropy plot of the sNMF analysis for the NZ dataset, averaged across 10 repetitions for K = 1-5. Lowest cross-entropy value indicates best support of particular K value.


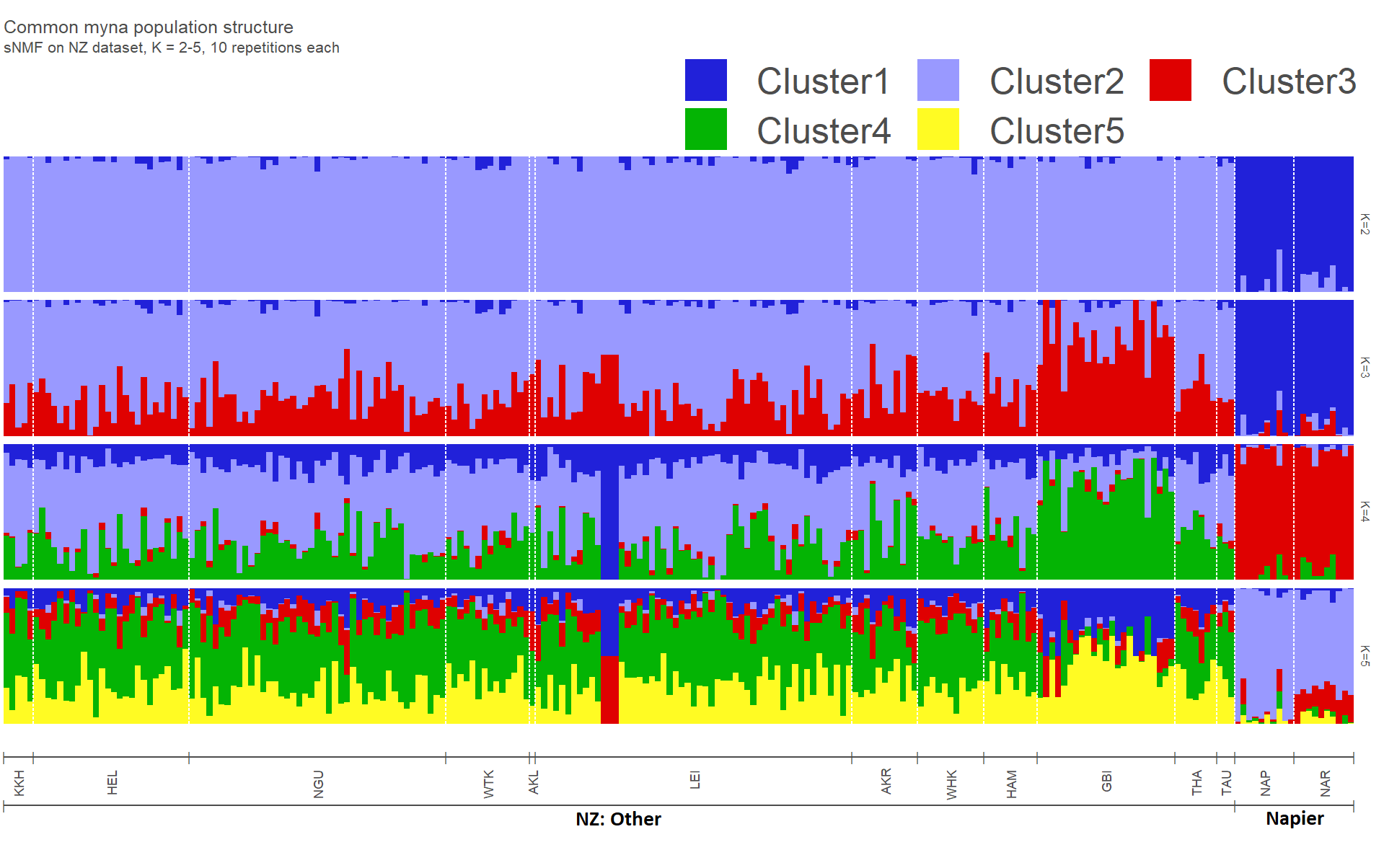


Figure S8.7 sNMF population structure plot for NZ dataset, K = 2-5, average across 10 replicates. Three-letter codes labelled beneath the figure refers to the labels in Figure 2 in the main text. KKH = Kaikohe (ROM), HEL = Helensville, NGU = Ngunguru, WTK = Waitakeres, AKL = Auckland, LEI = Leigh, AKR = Auckland (ROM), WHK = Waiheke, HAM = Hamilton (ROM), GBI = Great Barrier Island, THA = Thames, TAU = Taupo (ROM), NAP = Napier, NAR = Napier (ROM)

## IND dataset


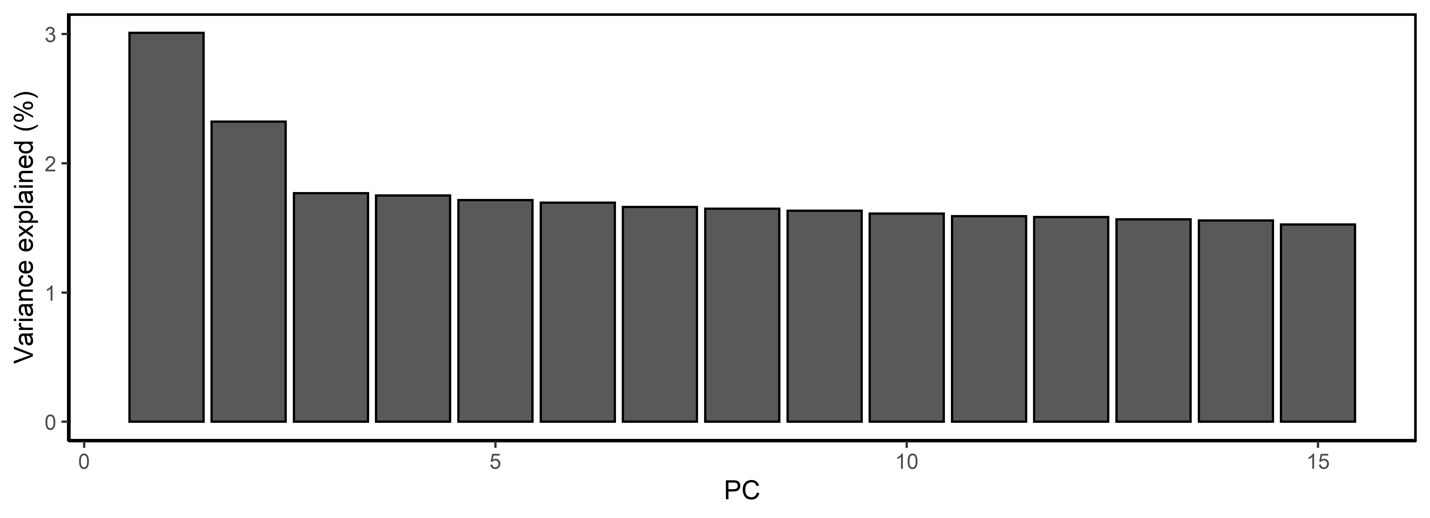


Figure S8.8 Scree plot of variance explained of the first 15 principal components from the PCA on the IND dataset


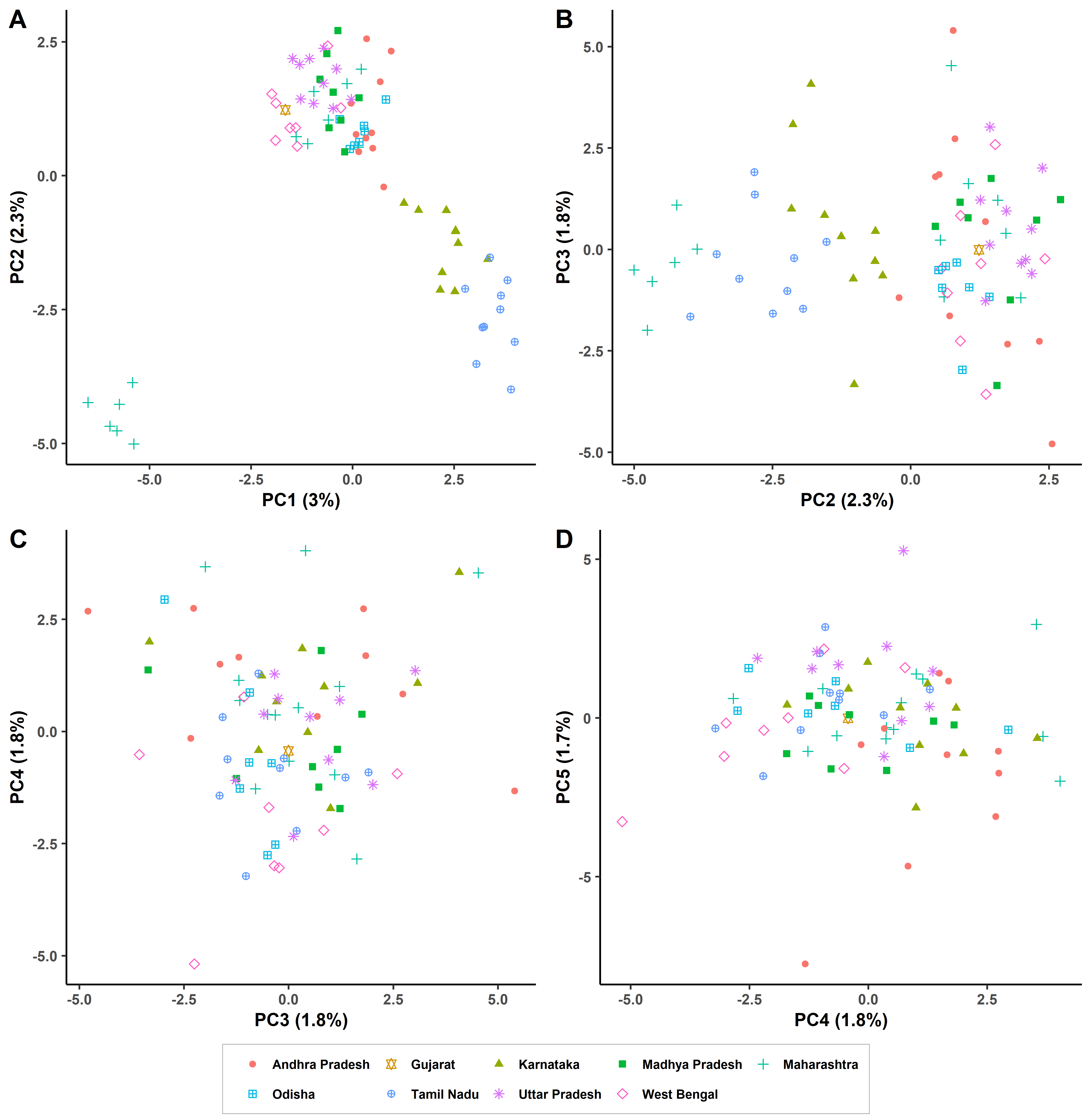


Figure S8.9 PCA plots of the IND dataset. A) PCA1 vs 2, B) PCA2 vs 3, C) PCA3 vs 4, and D) PCA4 vs 5. Samples are labelled based on location sampled. Note that six samples from Maharashtra cluster separately in PCA1 vs 2, corresponding to Maharashtra subpopulation A which cluster with Melbourne, Fiji, and Napier.


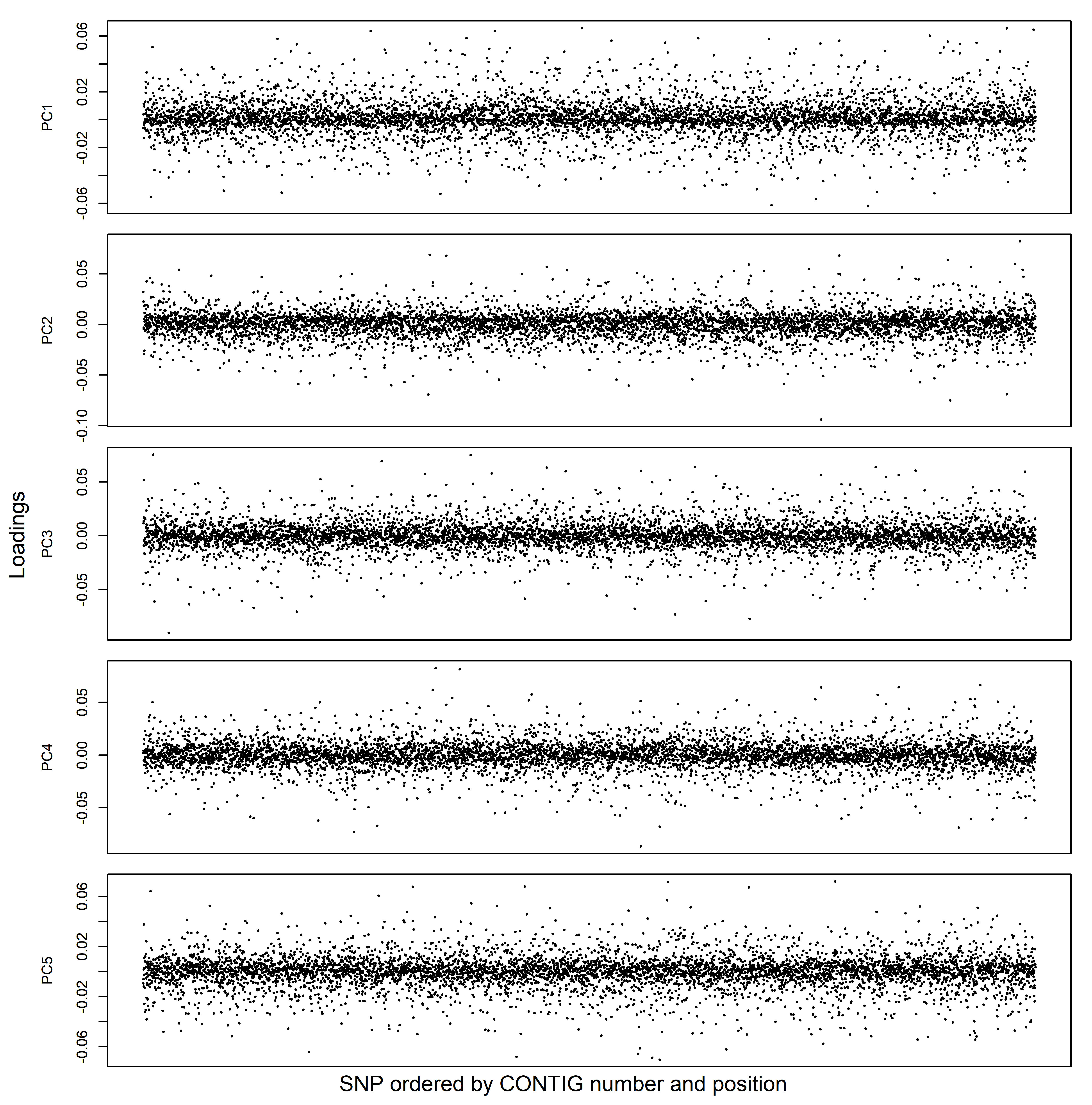


Figure S8.10 Loadings of PC1-5 from PCA on the IND dataset.


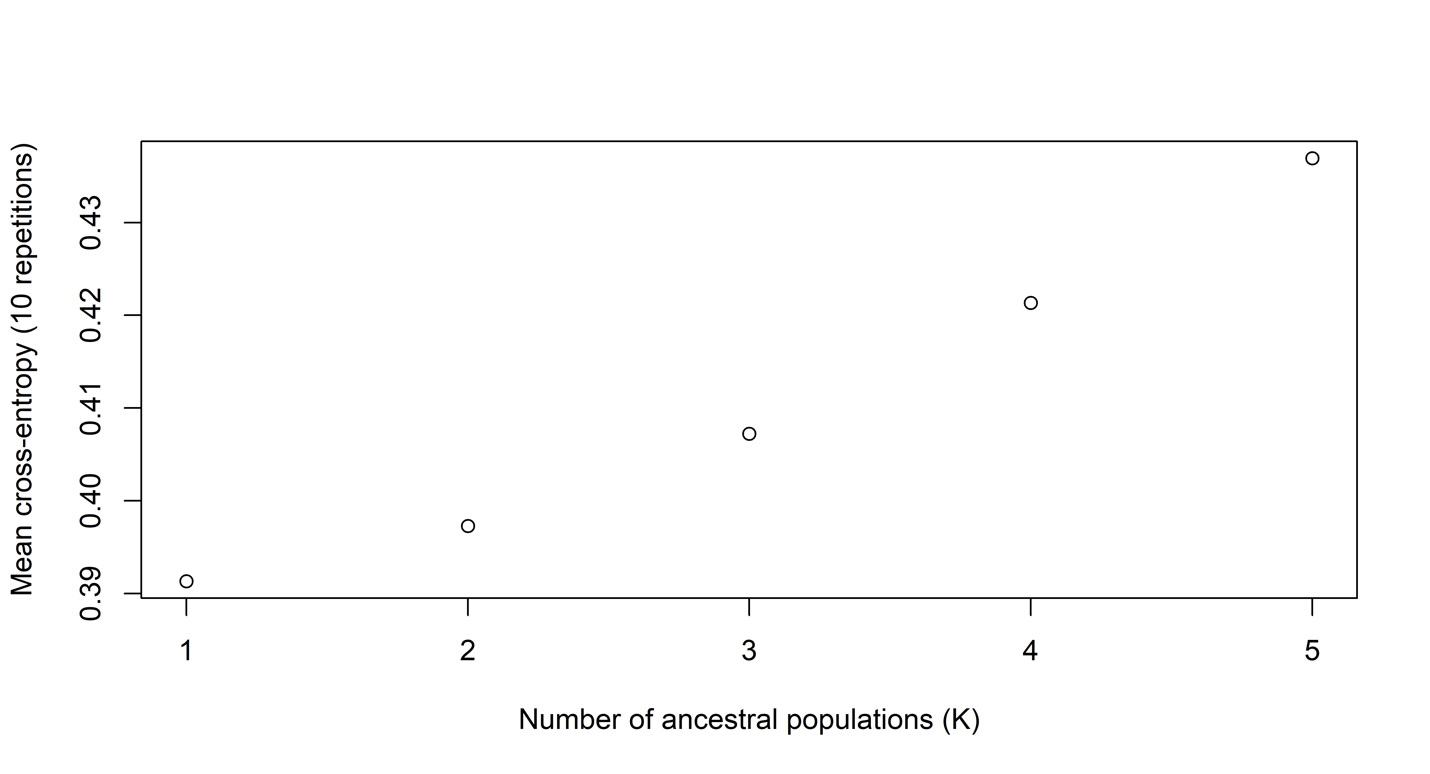


Figure S8.11 Mean cross-entropy plots of the sNMF analysis on the IND dataset, averaged across 10 repetitions at K = 1-5.


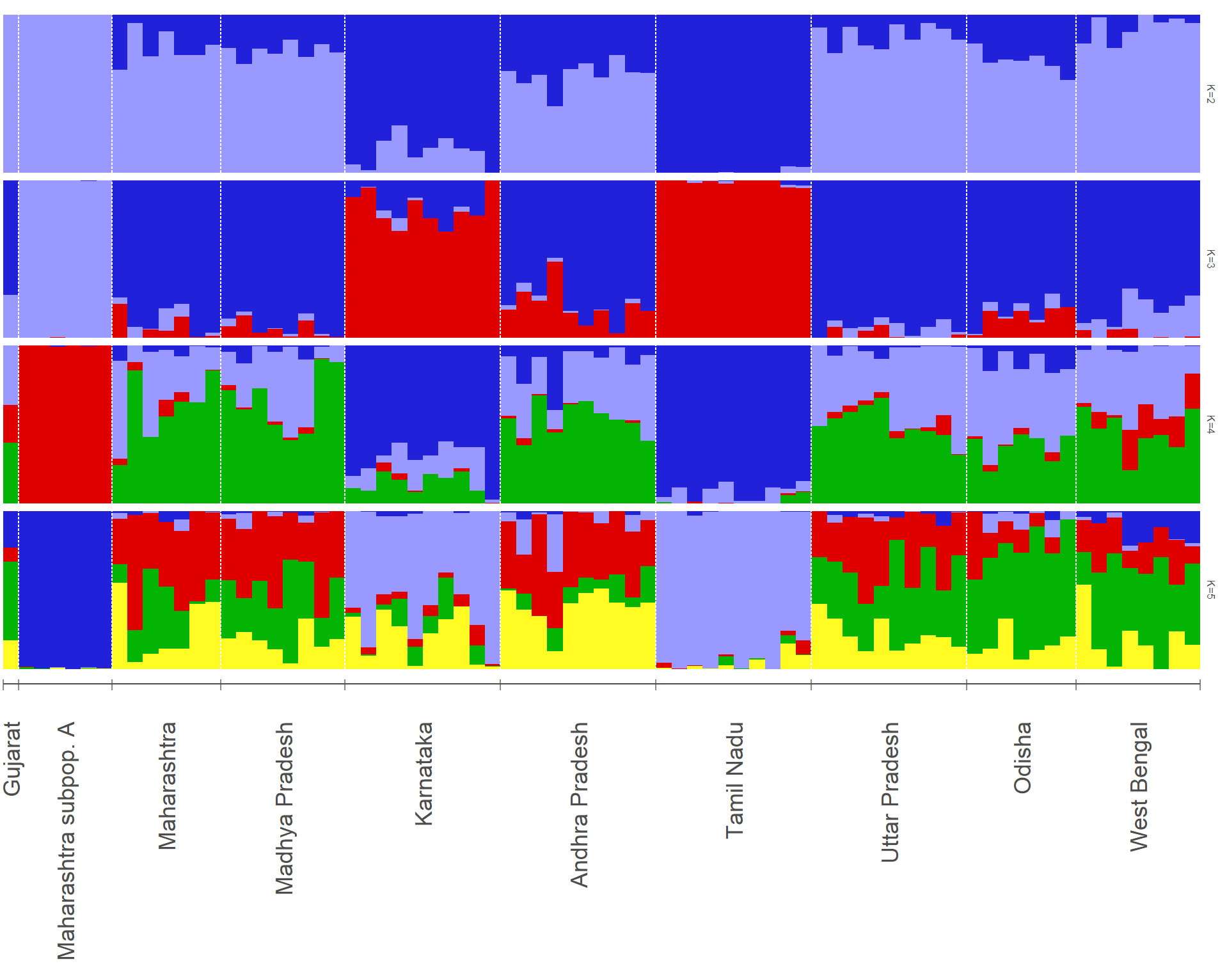


Figure S8.12 sNMF population structure plot for IND dataset, K = 2-5, average of 10 repetitions. Populations are defined based on popdef1 population definition.


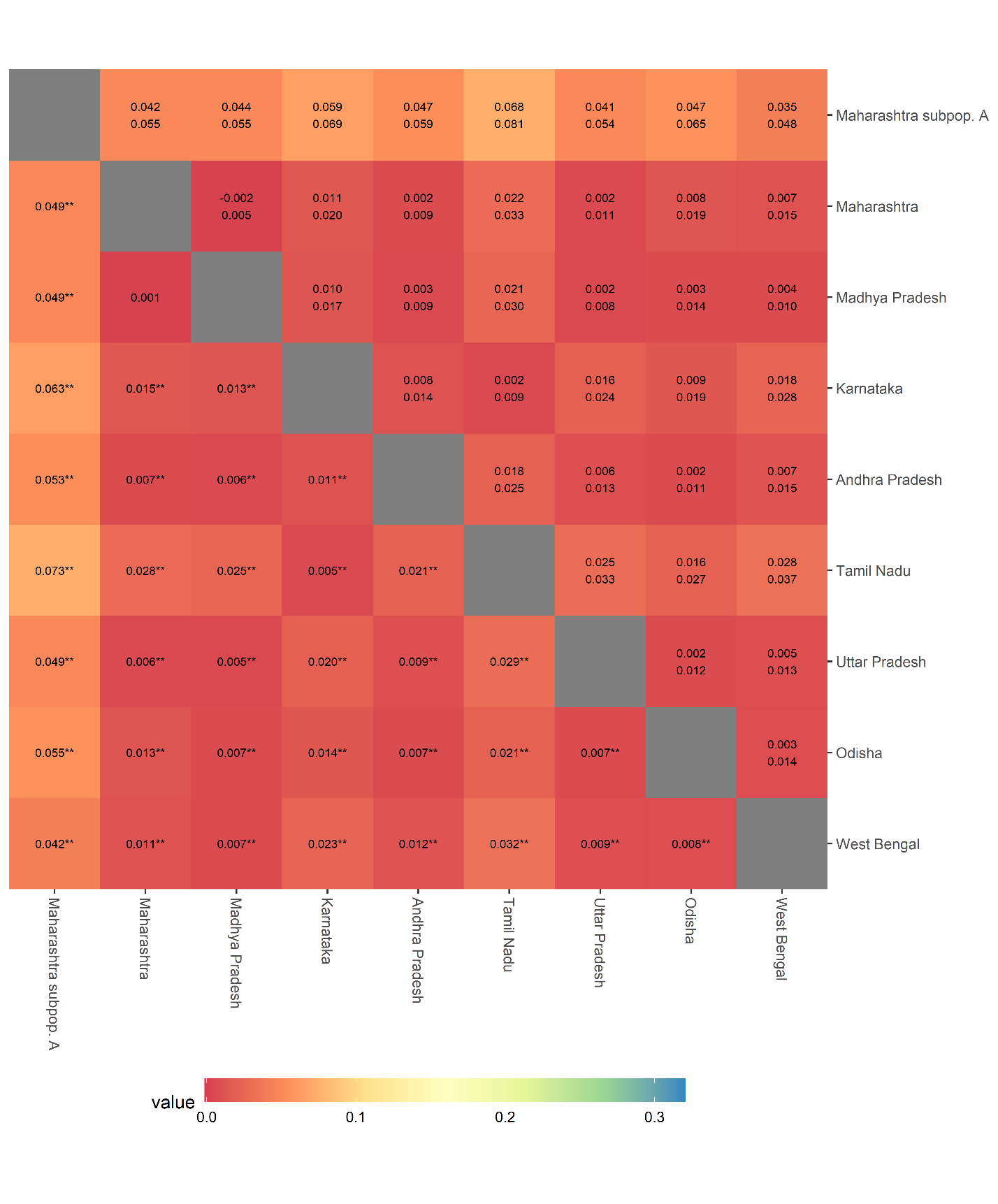


Figure S8.13

Heat map of population pairwise-F_ST_ comparisons (Weir and Cockerham, 1984) between all populations in the IND dataset based on 100 bootstrap replicates. Mean pairwise-F_ST_ are shown on the lower triangle, and the 95% lower and upper confidence interval is shown on the upper triangle. * = FST values with p-values < 0.05, ** = FST values with p-values < 0.01.

## ALL dataset (main text)


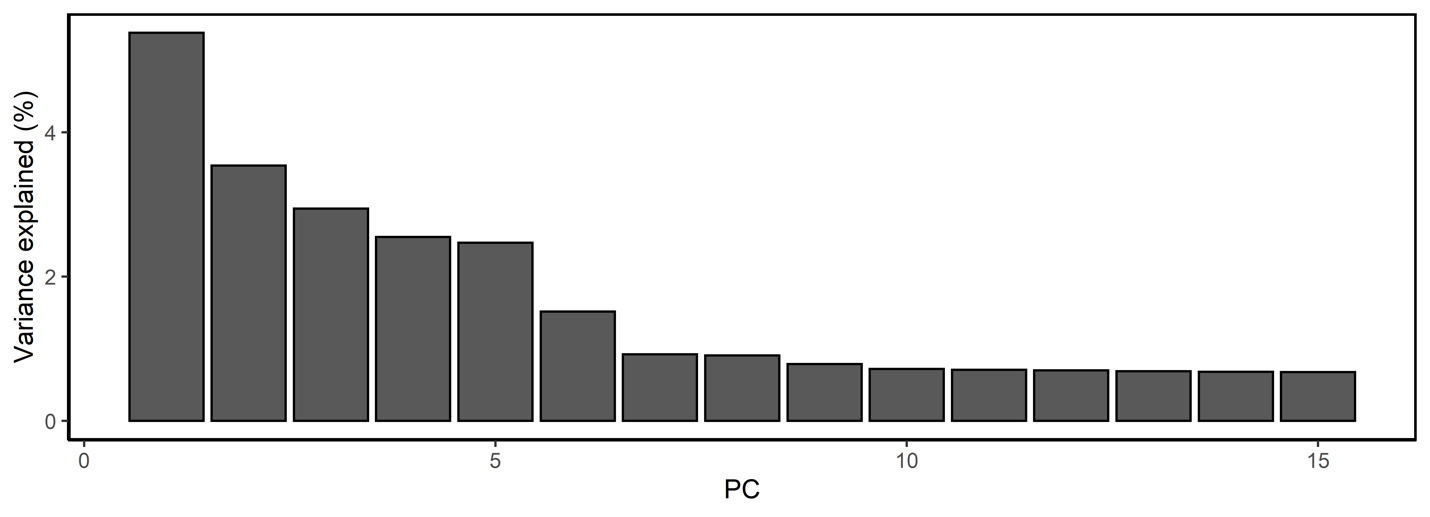


Figure S8.14

Scree plot of variance explained of the first 15 principal components from the PCA on the ALL dataset (subset to n ≤ 20 for introduced populations, as defined by popdef2). This screeplot corresponds to the PCA performed to produce Figure 5A in the main text.


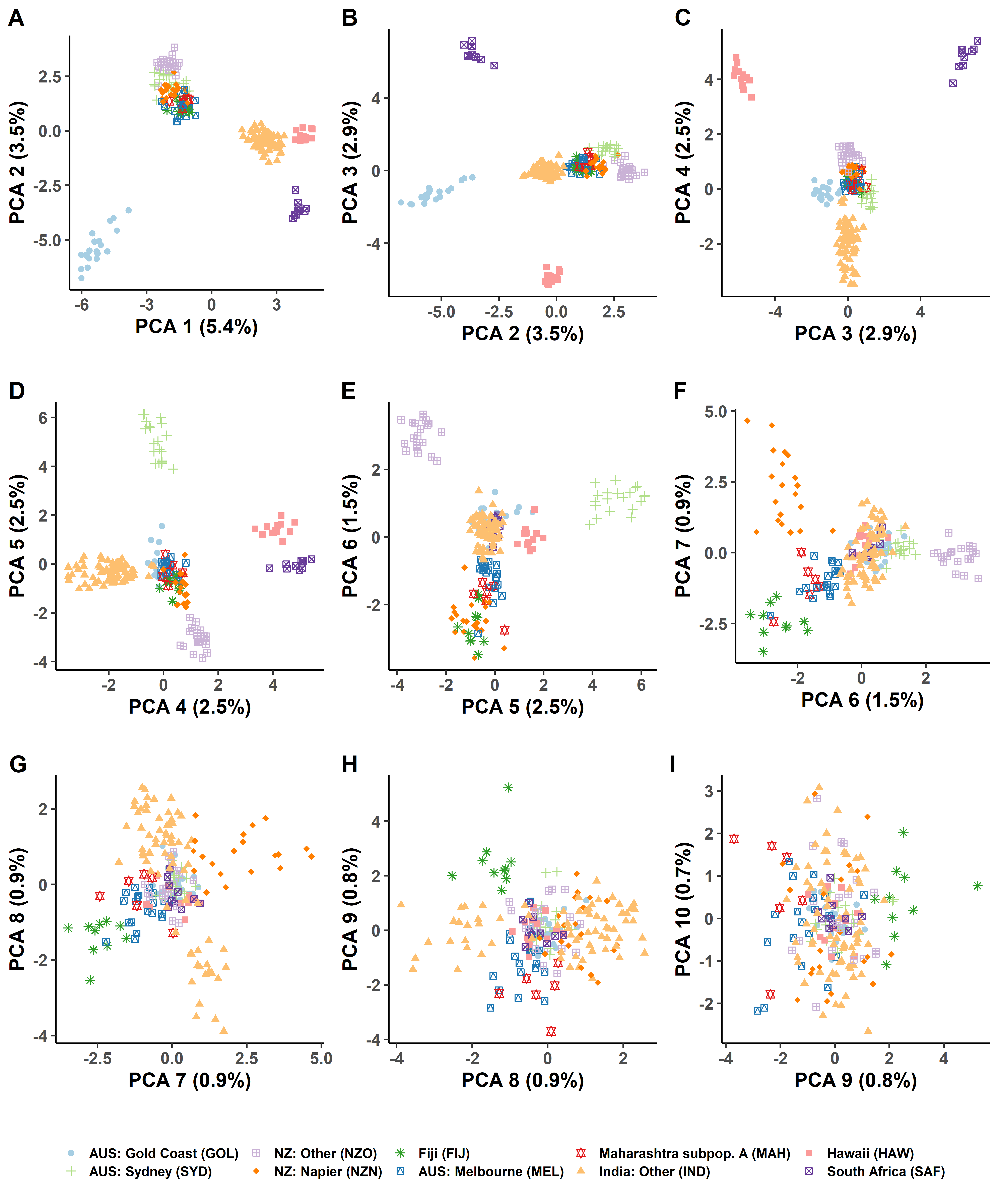


Figure S8.15 PCA plots of the ALL dataset (subset to n ≤ 20 for introduced populations, as defined by popdef2). PCA A) 1 vs 2, B) 2 vs 3, C) 3 vs 4, D) 4 vs 5, E) 5 vs 6, F) 6 vs 7, G) 7 vs 8, H) 8 vs 9, and I) 9 vs 10. Samples are labelled based on popdef2, same as in Figure 5A in the main text.


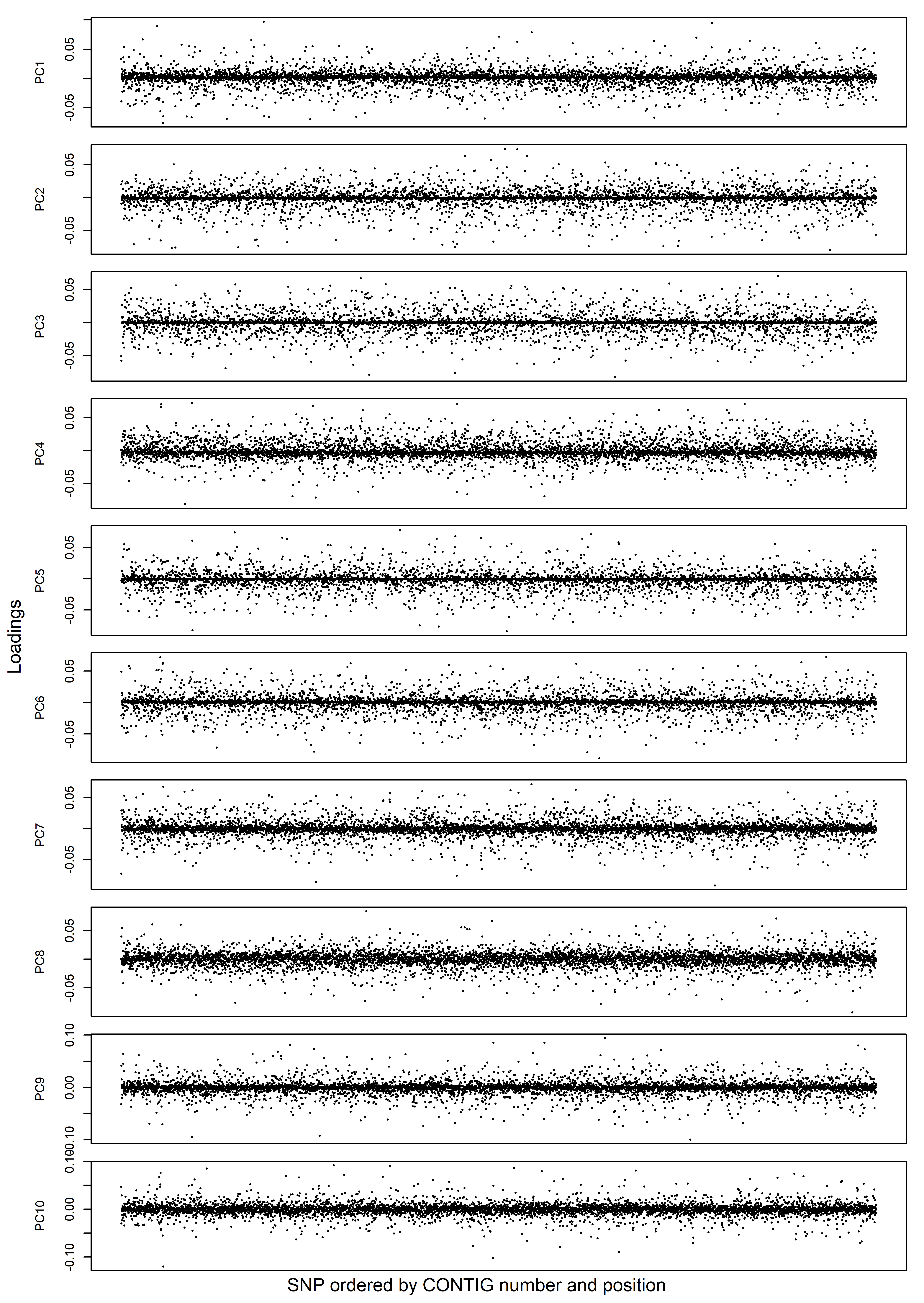


Figure S8.16 Loadings of PC1-10 from the PCA on the ALL dataset (subset to n ≤ 20 for introduced populations, as defined by popdef2).


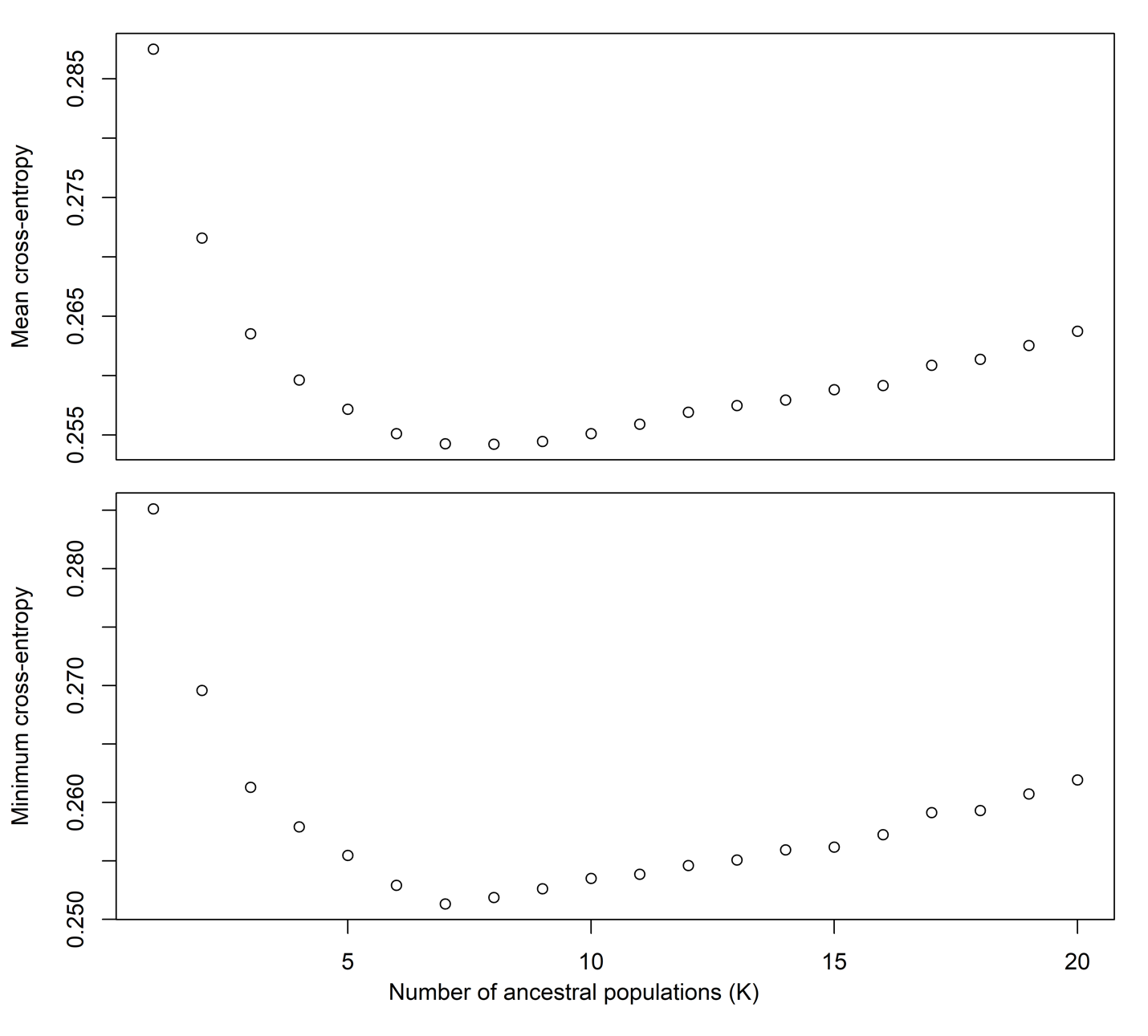


Figure S8.17 Minimum and mean cross-entropy plot from 10 repetitions for each K value. Lowest cross-entropy value indicates best support of particular K value. Note that the minimum cross-entropy suggests K=7 while the mean cross-entropy suggests K =8. However, the genetic groups defined appear to be the same.


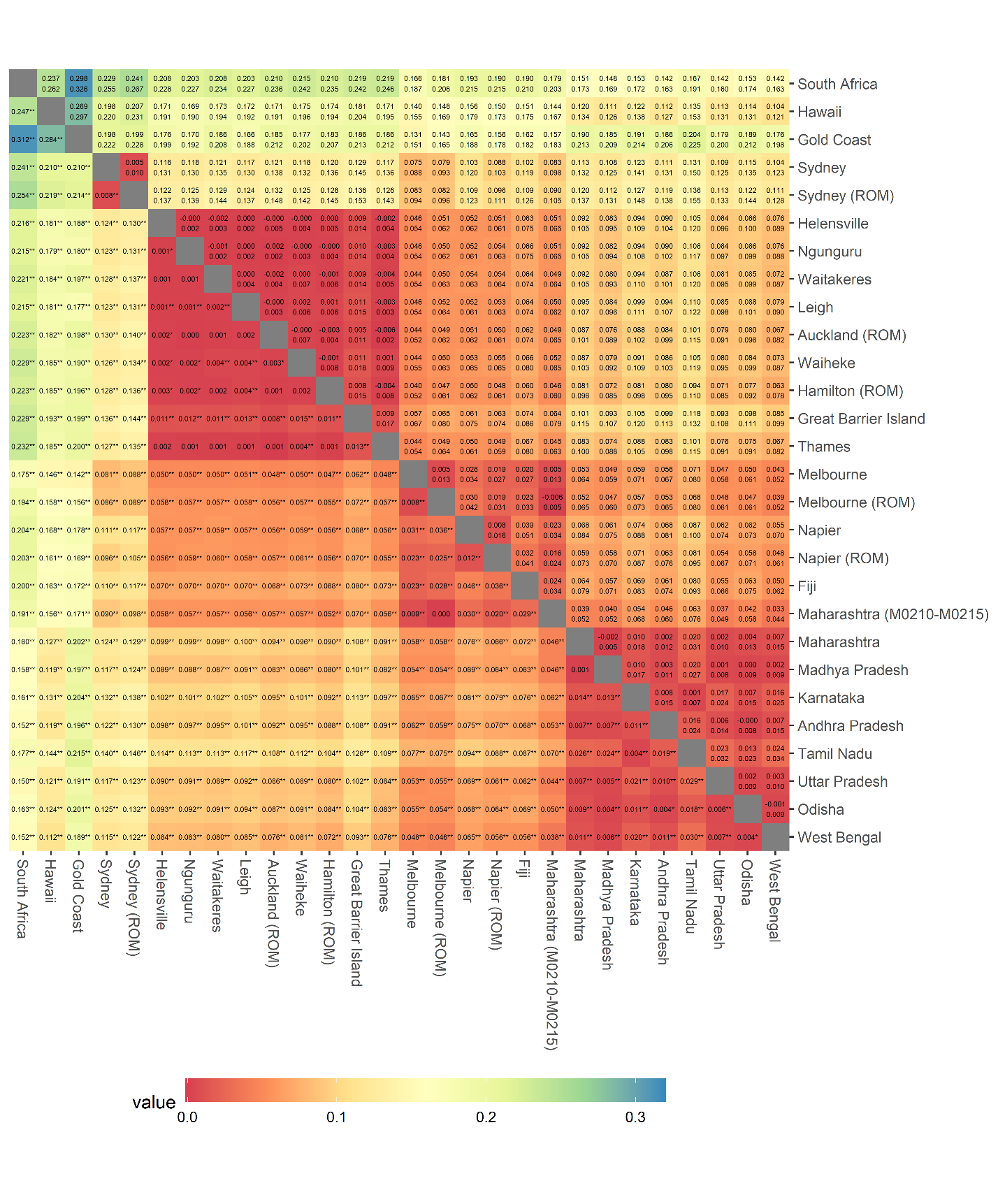


Figure S8.18 Heat map of population pairwise-F_ST_ between all populations in ALL dataset based on 100 bootstrapped replicates. Populations were defined using popdef1. Mean pairwise-F_ST_ are shown in the lower triangle, and the 95% lower and upper confidence interval is shown in the upper triangle. * = FST values with p-values < 0.05, ** = FST values with p-values < 0.01.

## ALL dataset with only relevant populations: nmax = 20


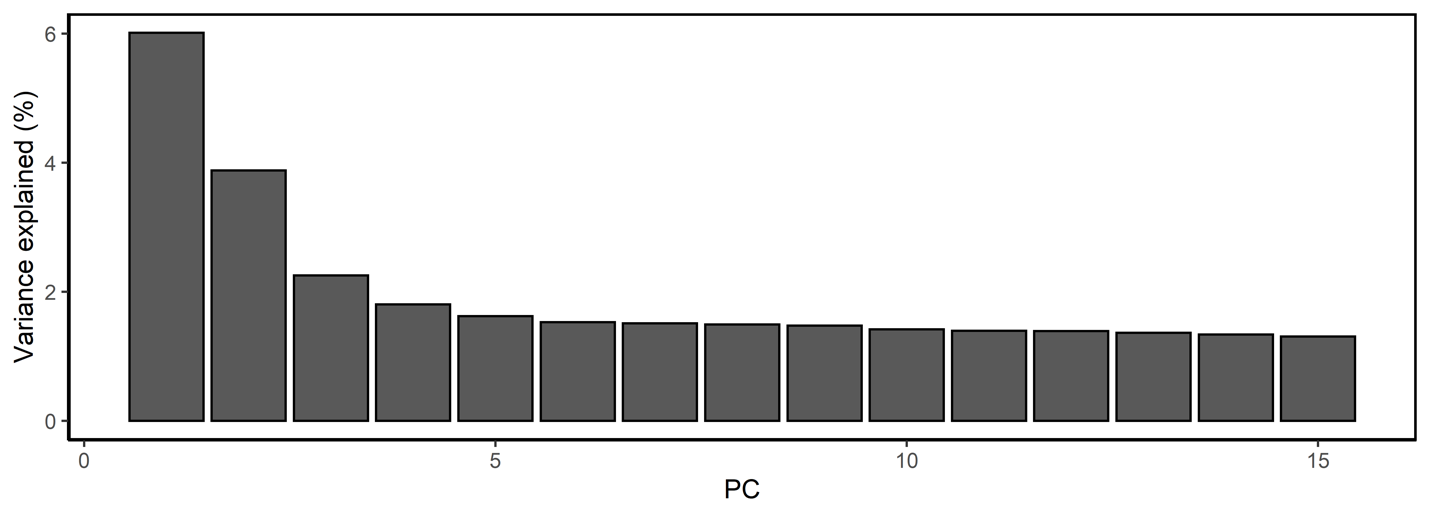


Figure S8.19 Scree plot of variance explained of the first 15 principal components from the PCA on the subset of the ALL dataset to include only relevant populations to New Zealand (subset to n ≤ 20 for introduced populations, as defined by popdef2, and only retaining NZ: Other, NZ: Napier, AUS: Melbourne, AUS: Sydney, Fiji, and Maharashtra subpopulation A). This screeplot corresponds to the PCA performed to produce Figure 5B in the main text.


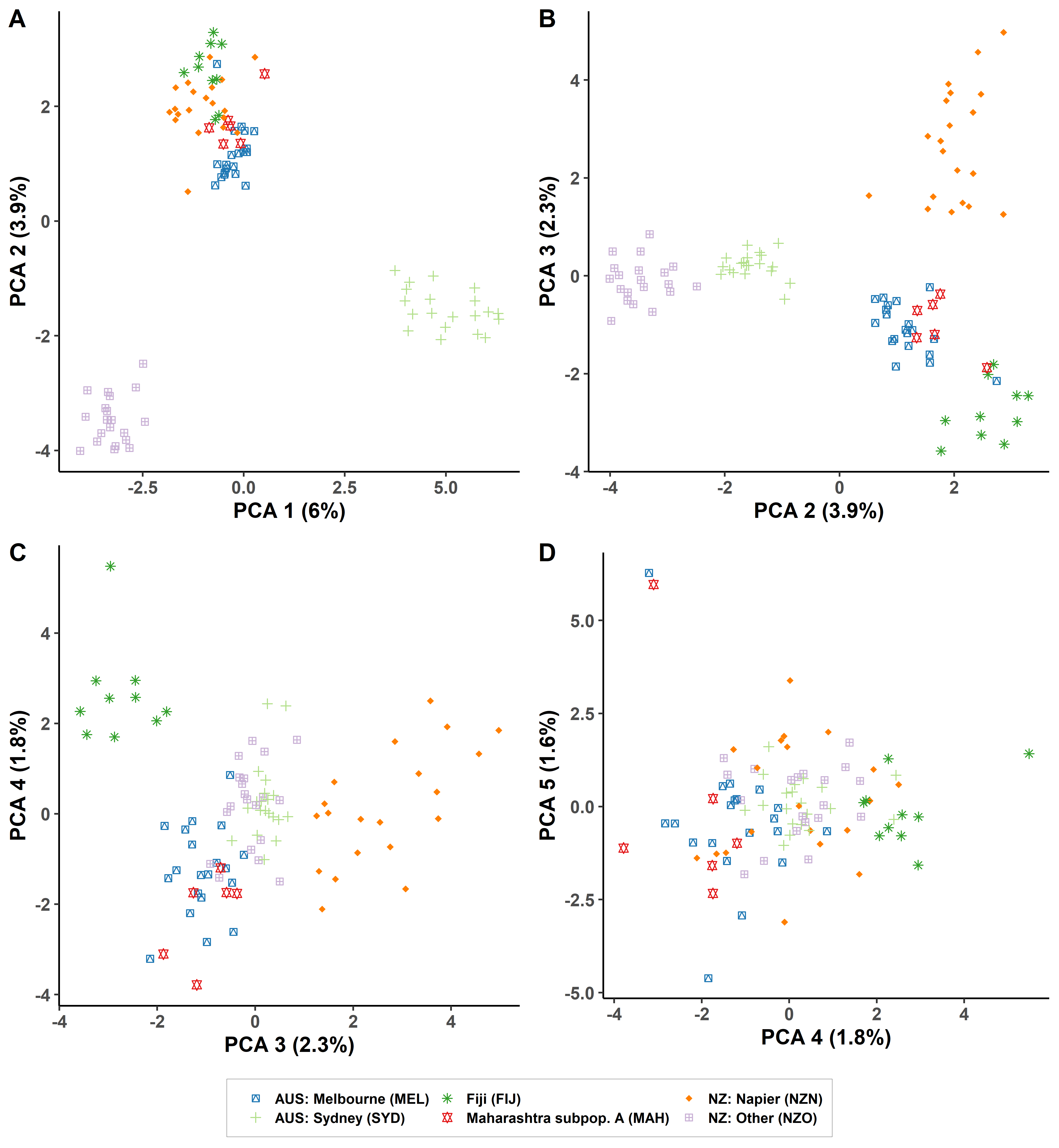


Figure S8.20 PCA plots of the subset of the ALL dataset to include only relevant populations to New Zealand (subset to n ≤ 20 for introduced populations, as defined by popdef2, and only retaining NZ: Other, NZ: Napier, AUS: Melbourne, AUS: Sydney, Fiji, and Maharashtra subpopulation A). These plots corresponds to the PCA performed to produce Figure 5B in the main text. PCA A) 1 vs 2, B) 2 vs 3, C) 3 vs 4, and D) 4 vs 5, E).


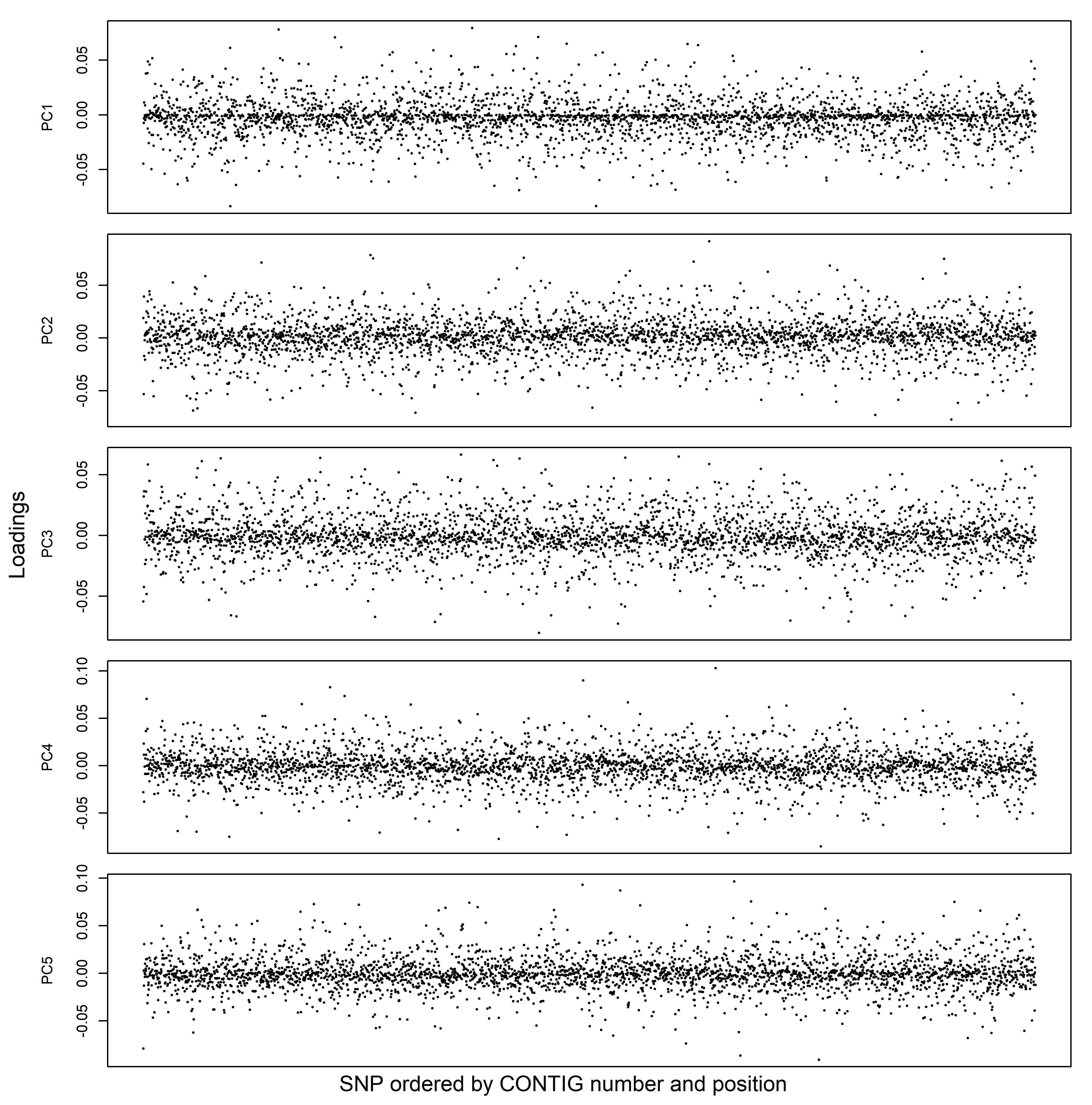


Figure S8.21 Loadings of PC1-5 from the PCA on the subset of the ALL dataset to include only relevant populations to New Zealand (subset to n ≤ 20 for introduced populations, as defined by popdef2, and only retaining NZ: Other, NZ: Napier, AUS: Melbourne, AUS: Sydney, Fiji, and Maharashtra subpop.


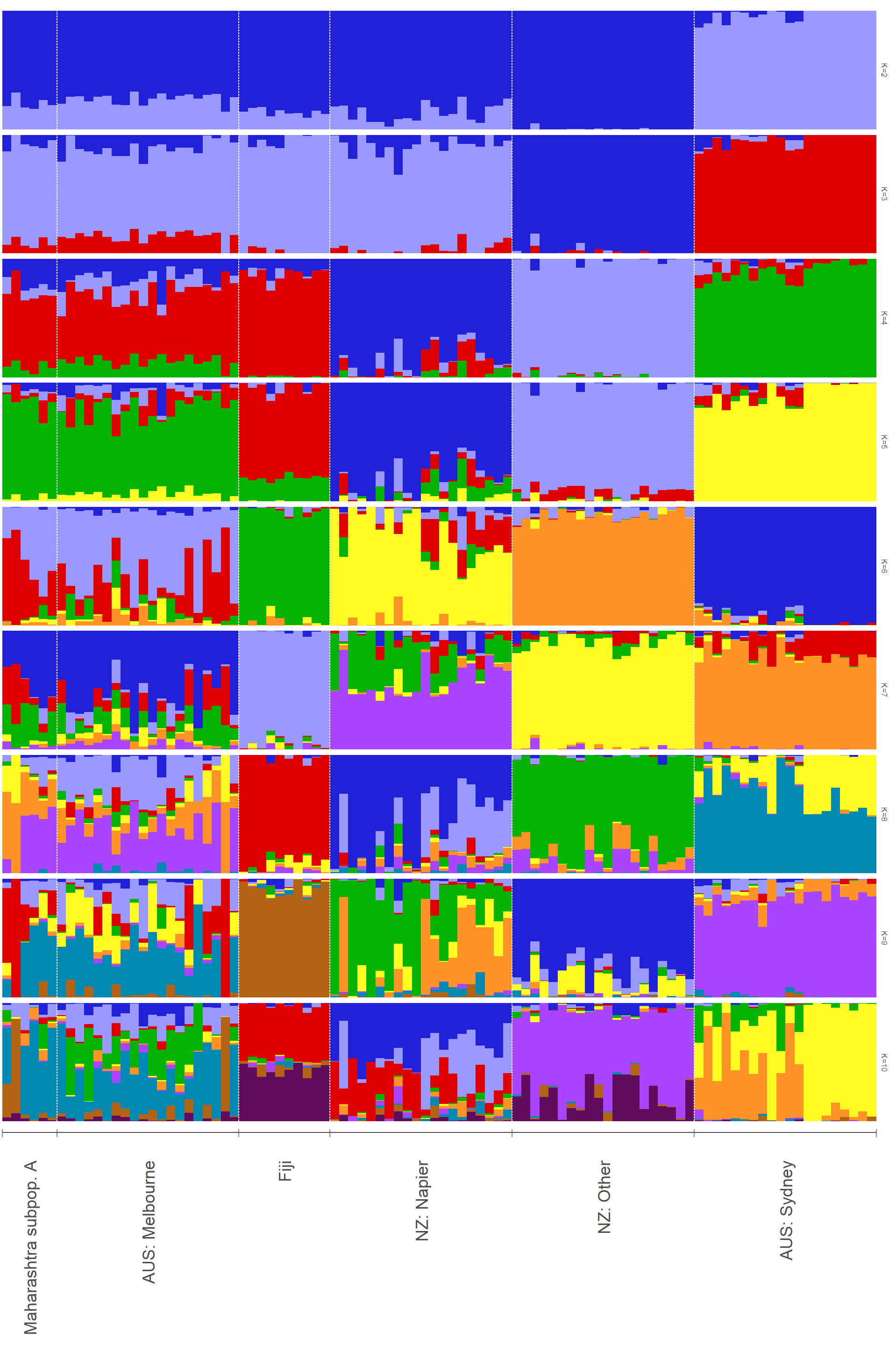


Figure S8.22 sNMF population structure plot for the subset of the ALL dataset to include only relevant populations to New Zealand (subset to n ≤ 20 for introduced populations, as defined by popdef2, and only retaining NZ: Other, NZ: Napier, AUS: Melbourne, AUS: Sydney, Fiji, and Maharashtra subpopulation A).

## ALL dataset: No SNP thinning


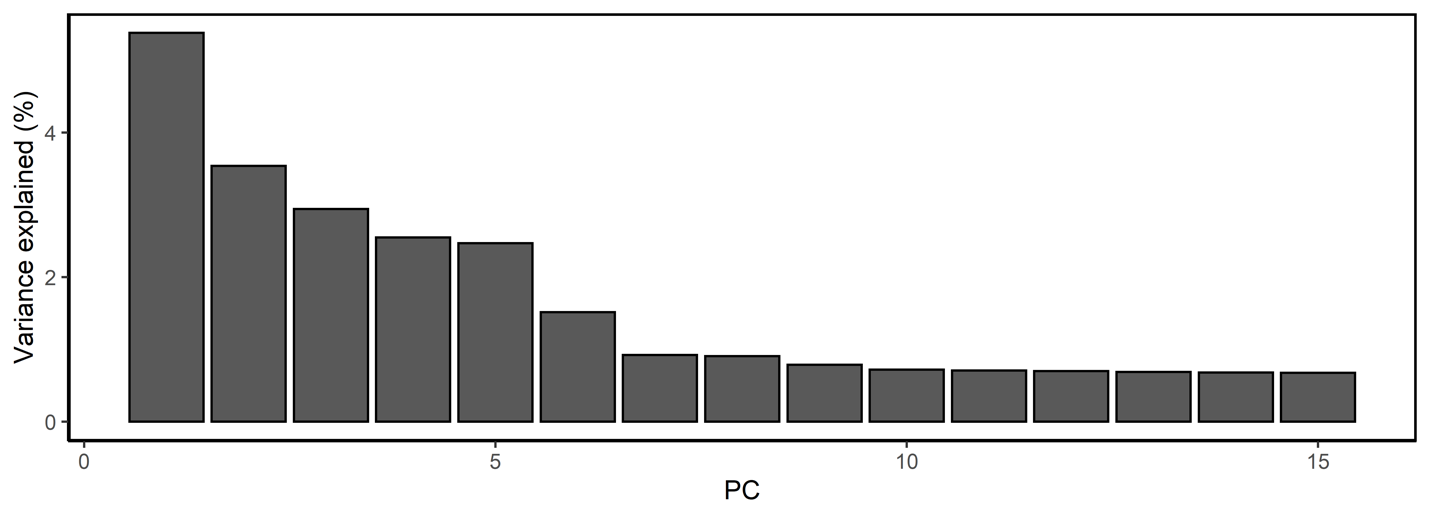


Figure S8.23 Scree plot of variance explained of the first 15 principal components from the PCA on the ALL dataset without SNP thinning (32,470 SNPs).


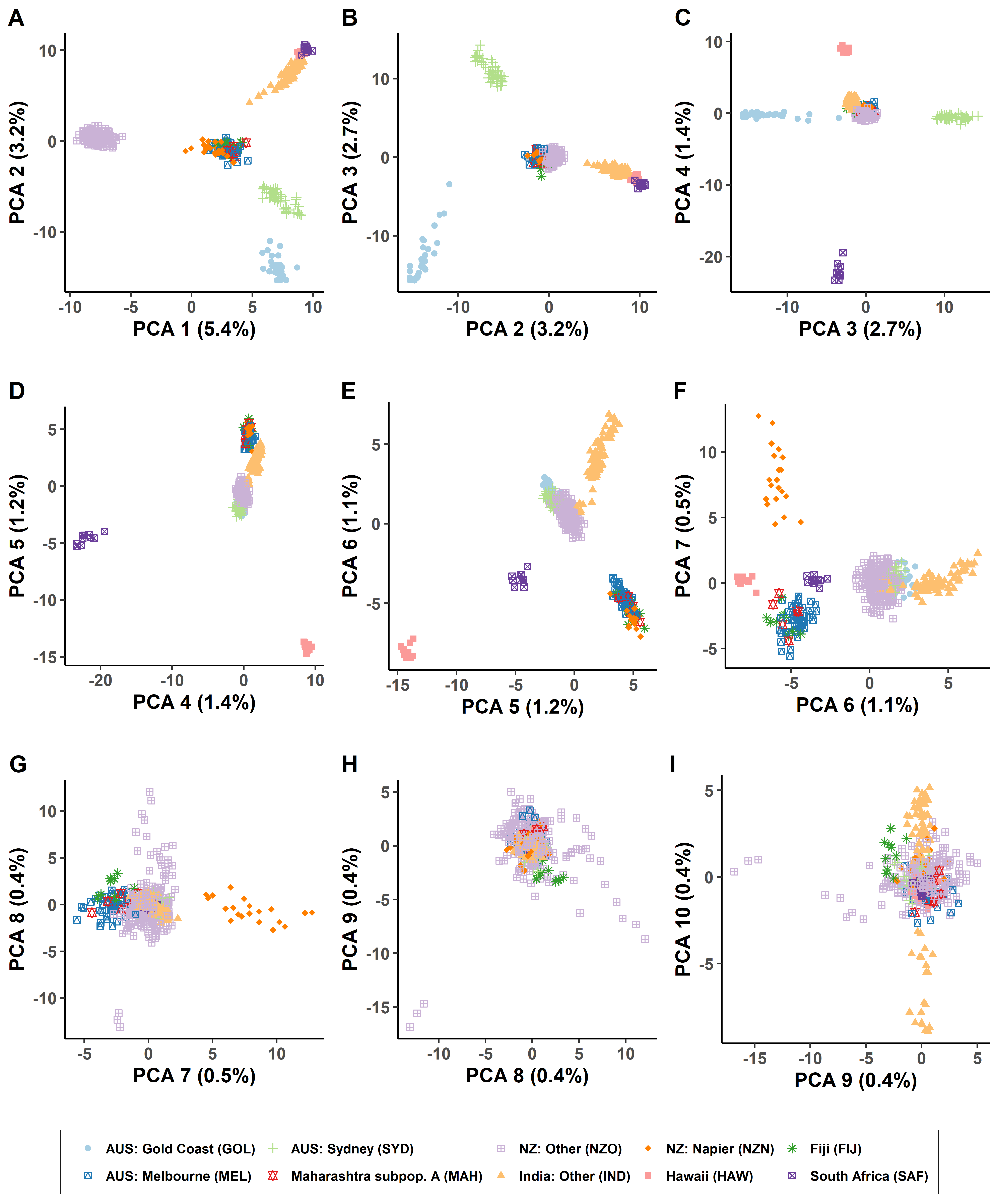


Figure S8.24 PCA plots of the ALL dataset without SNP thinning (32,470 SNPs). PCA A) 1 vs 2, B) 2 vs 3, C) 3 vs 4, D) 4 vs 5, E) 5 vs 6, F) 6 vs 7, G) 7 vs 8, H) 8 vs 9, and I) 9 vs 10. Samples are labelled based on popdef2, same as in Figure 5A in the main text.


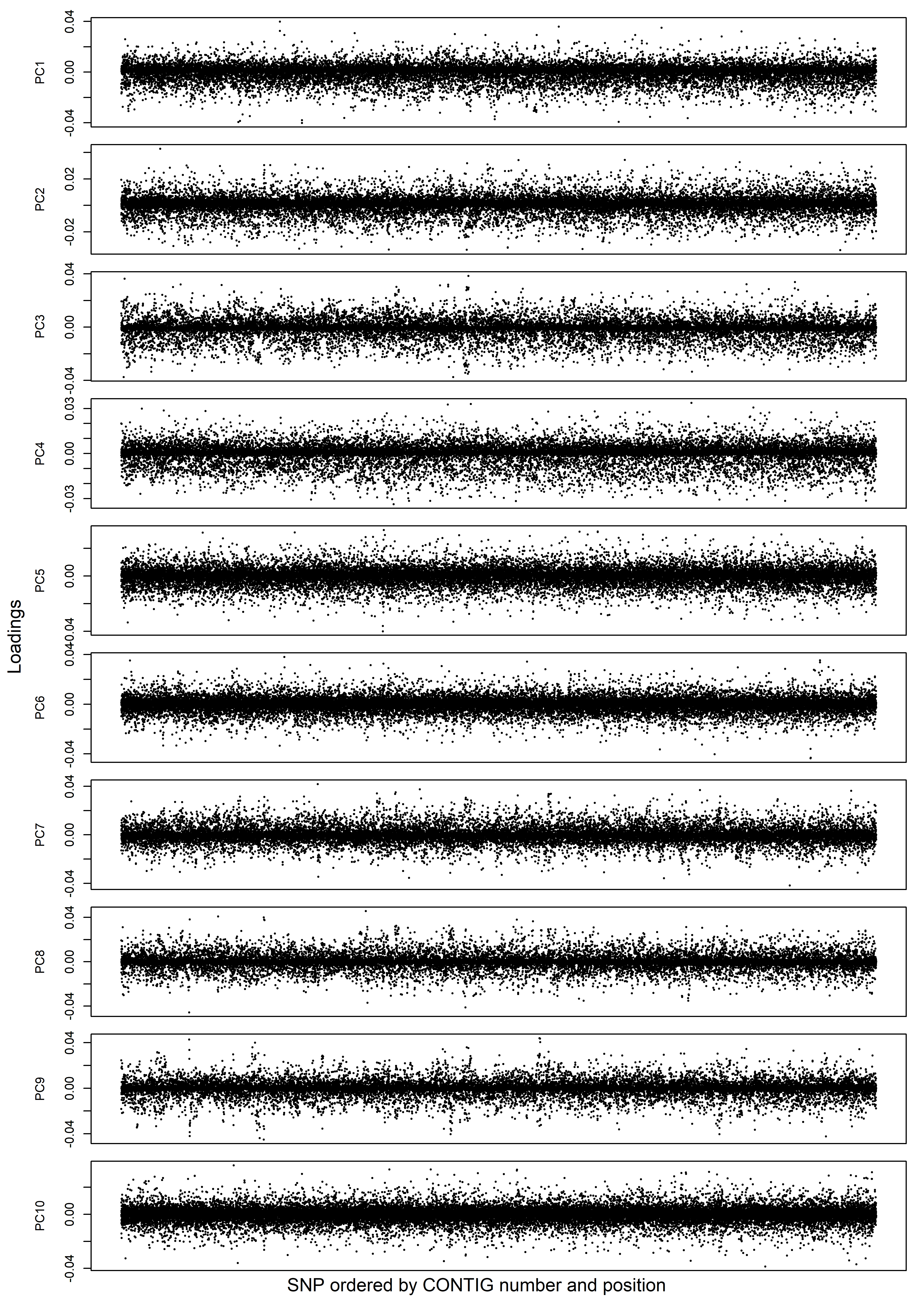


Figure S8.25 Loadings of PC1-10 from the PCA on the ALL dataset without SNP thinning (32,470 SNPs). Note that in there are potential patterns influenced by linkage, especially on PC3 and PC9 (clusters of SNPs with high loadings).


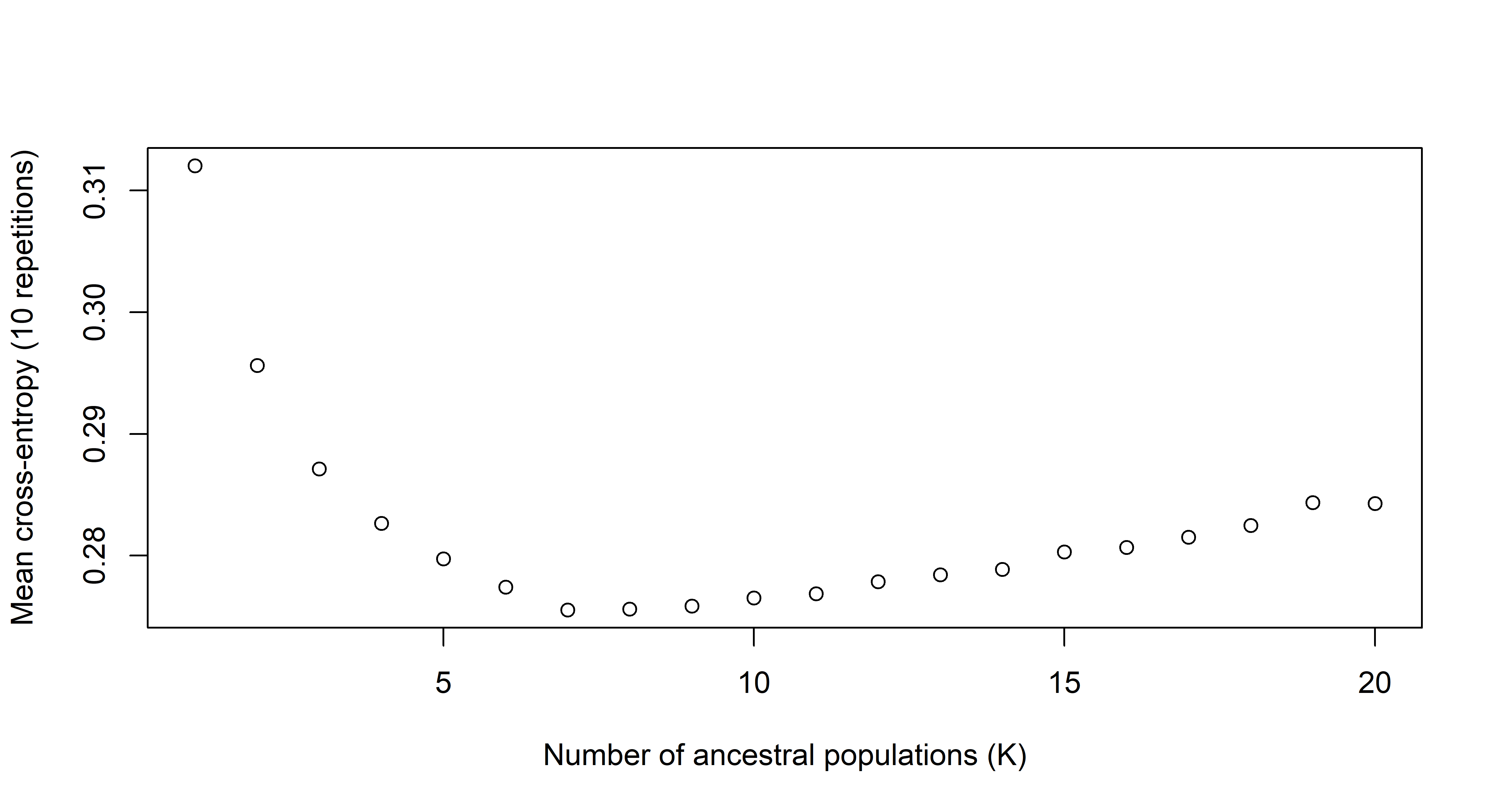


Figure S8.26 Mean cross-entropy plot of the sNMF analysis on the ALL dataset without SNP thinning (32,470 SNPs), averaged across 10 repetitions for each K value. Lowest cross-entropy value indicate best support of particular K value.


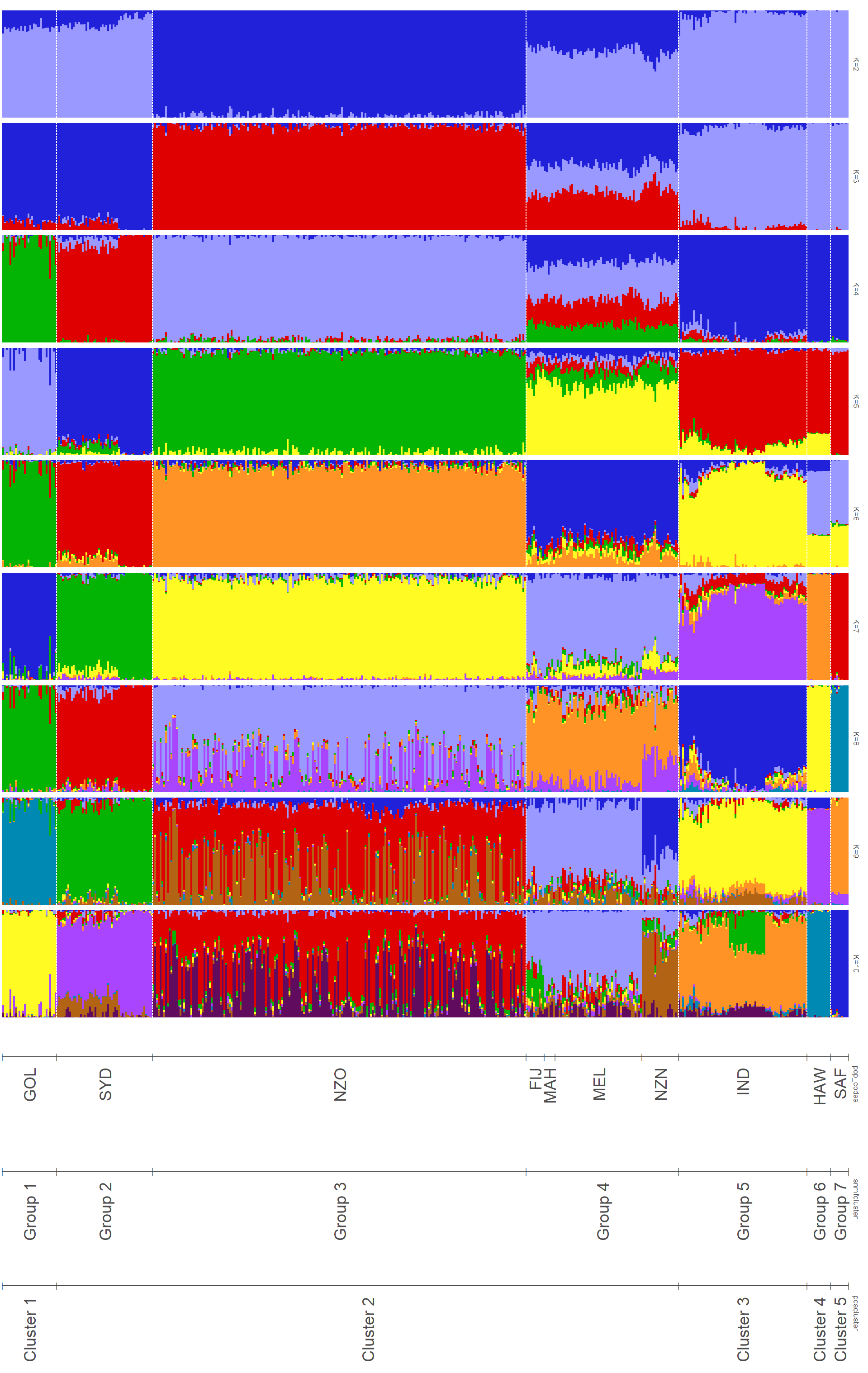


Figure S8.27 sNMF population structure plot for the ALL dataset without SNP thinning (32,470 SNPs).

## ALL dataset: nmax = 20, MAF > 0.05, thinned

A total of 4488 SNPs were retained.


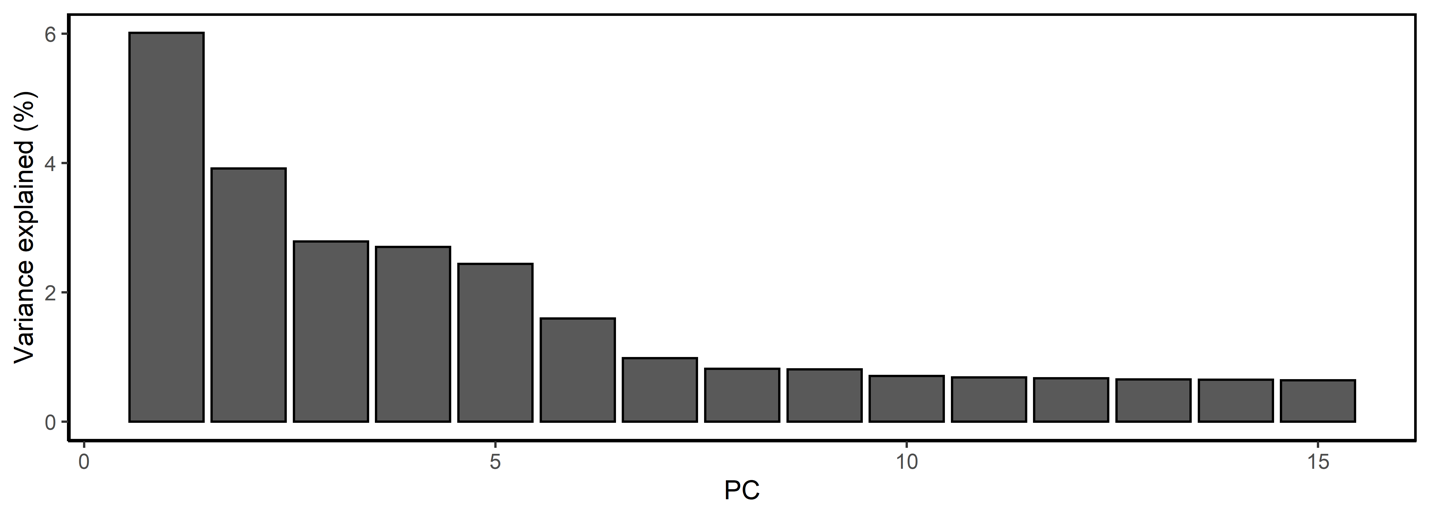


Figure S8.28 Scree plot of variance explained of the first 15 principal components from the PCA on the ALL dataset, subset to n ≤ 20 for introduced populations, as defined by popdef2, MAF > 0.05, and thinned to retain one SNP every 100,000 base pairs. The same samples were retained as the dataset used to create Figure 5A in the main text.


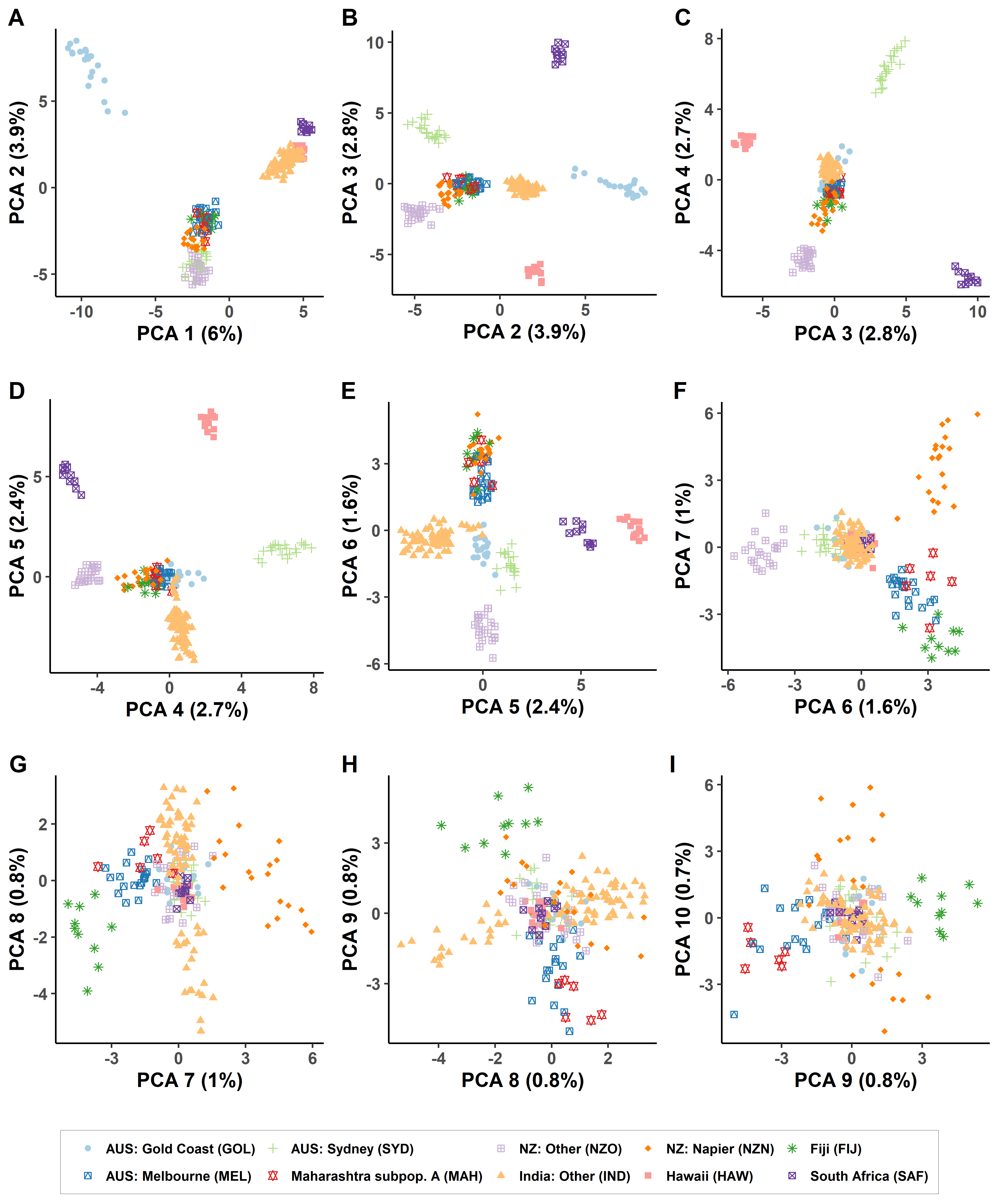


Figure S8.29 PCA plots of the ALL dataset, subset to n ≤ 20 for introduced populations, as defined by popdef2, MAF > 0.05, and thinned to retain one SNP every 100,000 base pairs. The same samples were retained as the dataset used to create Figure 5A in the main text. PCA A) 1 vs 2, B) 2 vs 3, C) 3 vs 4, D) 4 vs 5, E) 5 vs 6, F) 6 vs 7, G) 7 vs 8, H) 8 vs 9, and I) 9 vs 10. Samples are labelled based on popdef2.


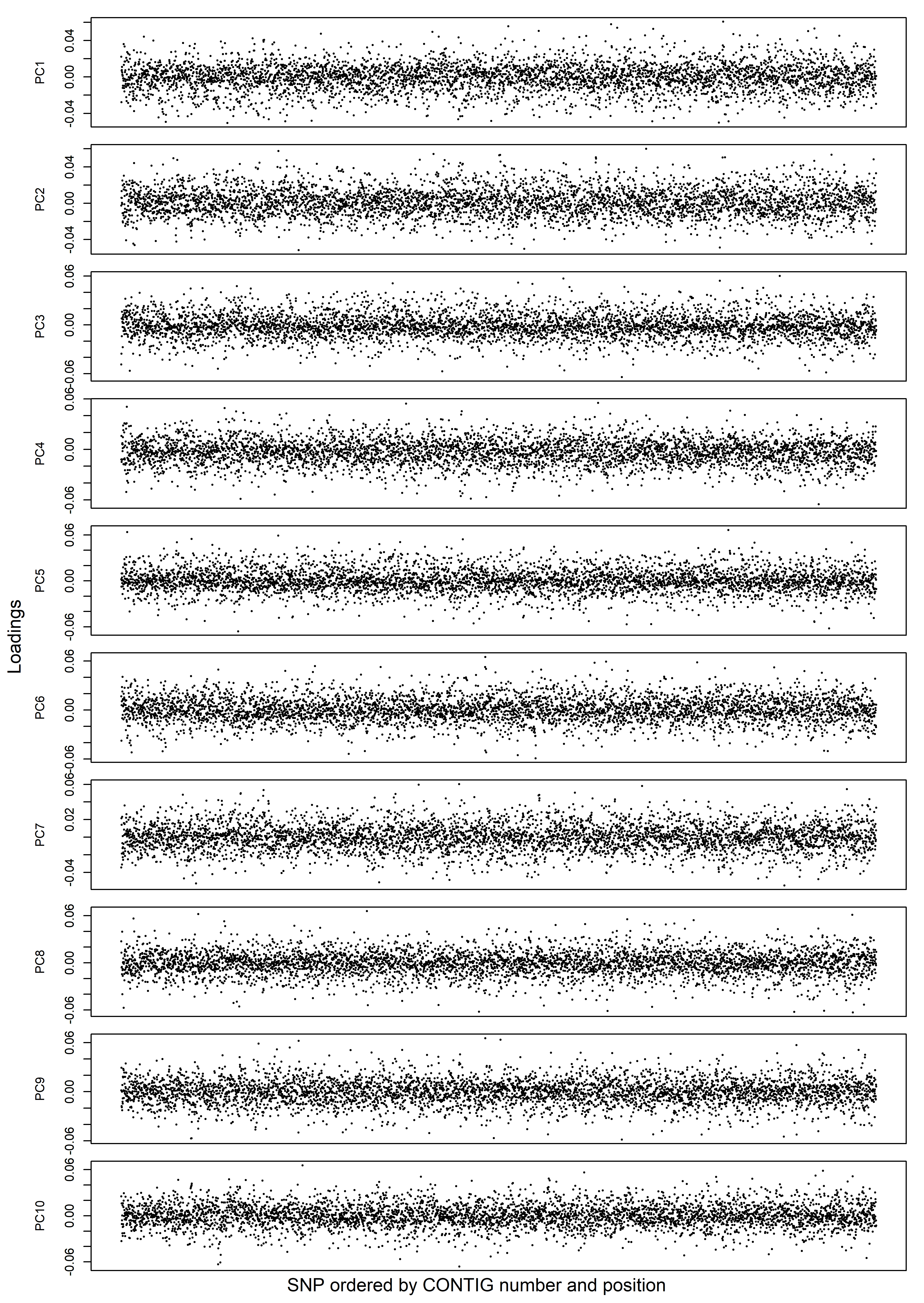


Figure S8.30 Loadings of PC1-10 from the PCA on the ALL dataset, subset to n ≤ 20 for introduced populations, as defined by popdef2, MAF > 0.05, and thinned to retain one SNP every 100,000 base pairs.


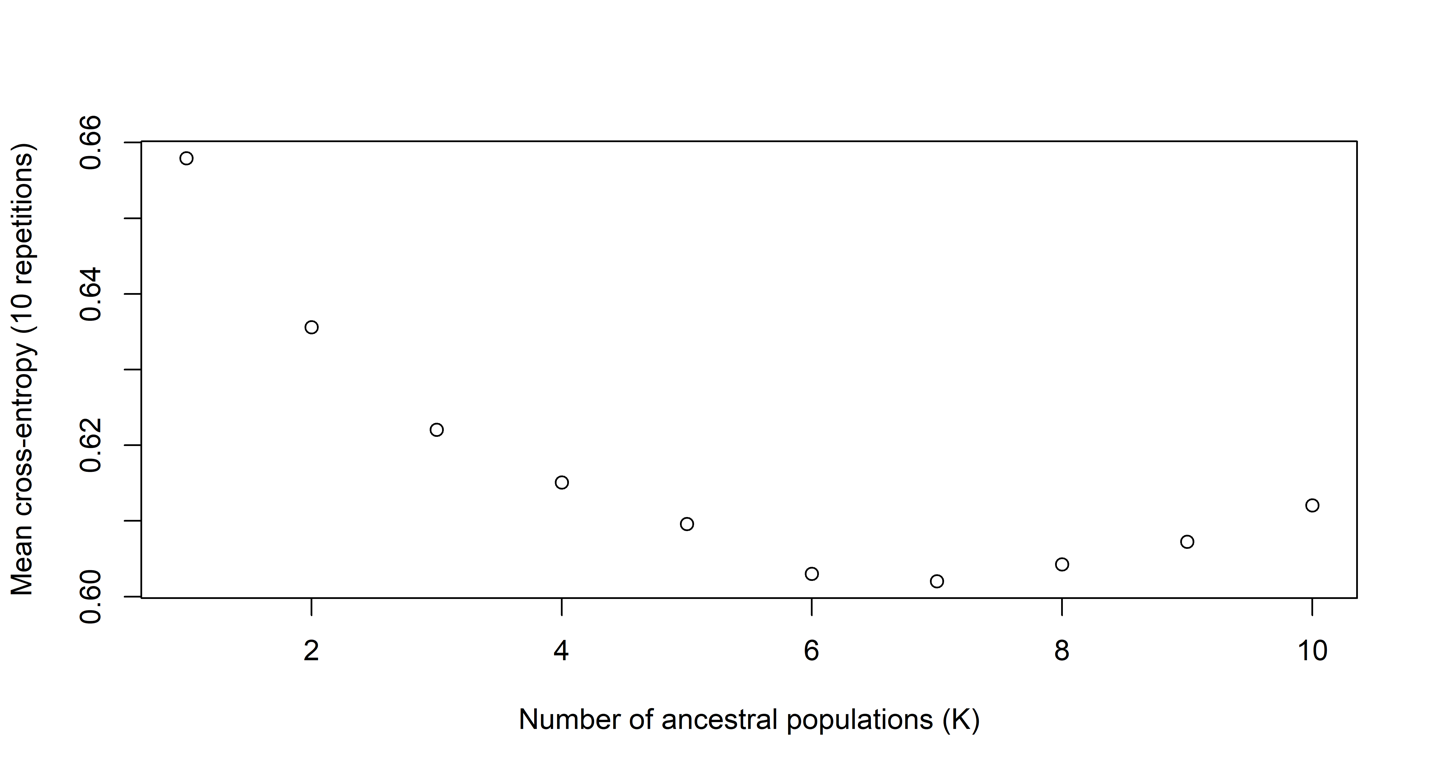


Figure S8.31 Mean cross-entropy plot of the sNMF analysis on the ALL dataset, subset to n ≤ 20 for introduced populations, as defined by popdef2, MAF > 0.05, and thinned to retain one SNP every 100,000 base pairs, averaged across 10 repetitions for each K value. Lowest cross-entropy value indicate best support of particular K value (K=7).


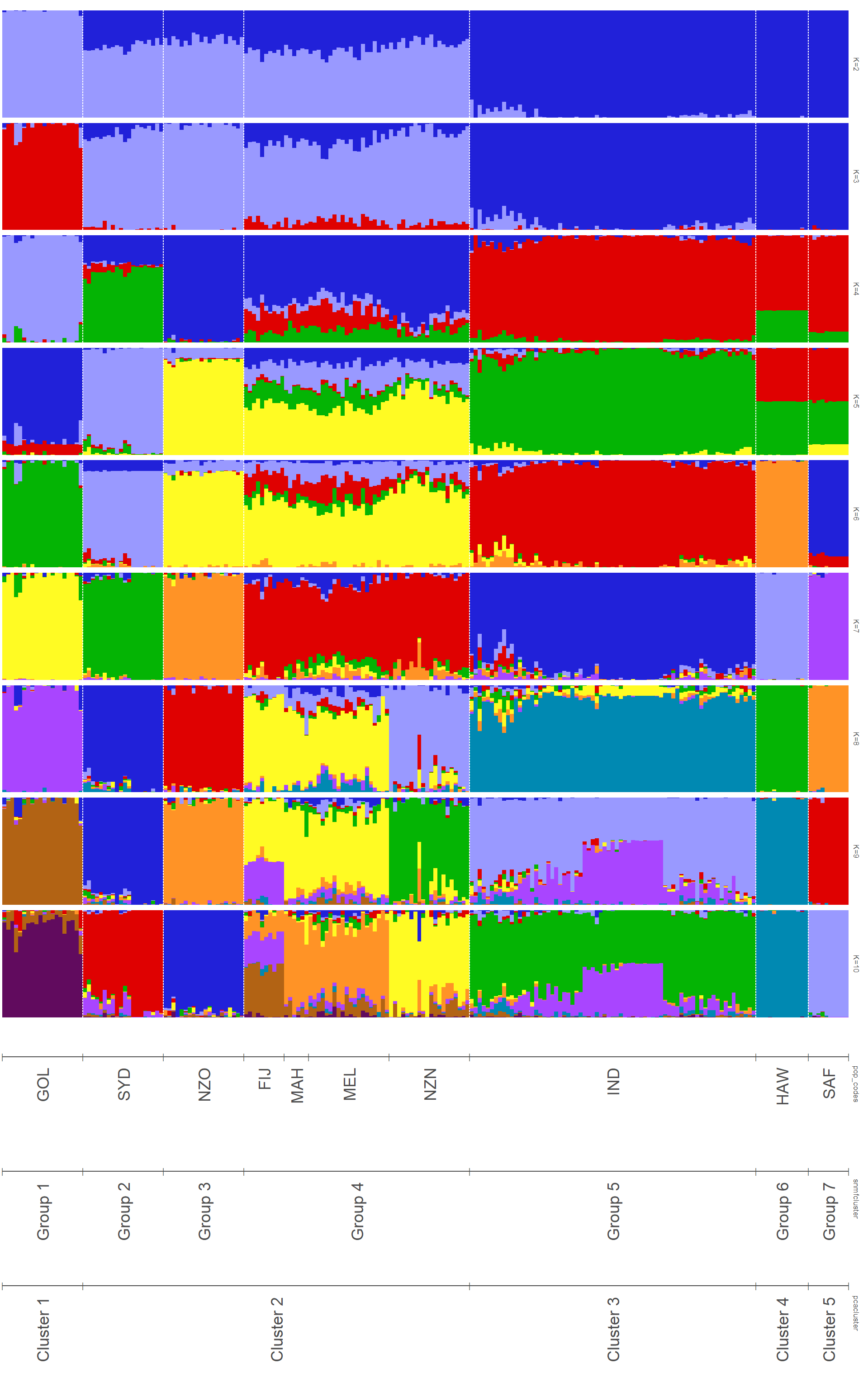


Figure S8.32 sNMF population structure plot for the ALL dataset, subset to n ≤ 20 for introduced populations, as defined by popdef2, MAF > 0.05, and thinned to retain one SNP every 100,000 base pairs, averaged across 10 repetitions for K = 2-10. Lowest mean cross-entropy value: K = 7.

## ALL dataset: nmax = 20, MAF > 0.1, thinned

A total of 3553 SNPs were retained.


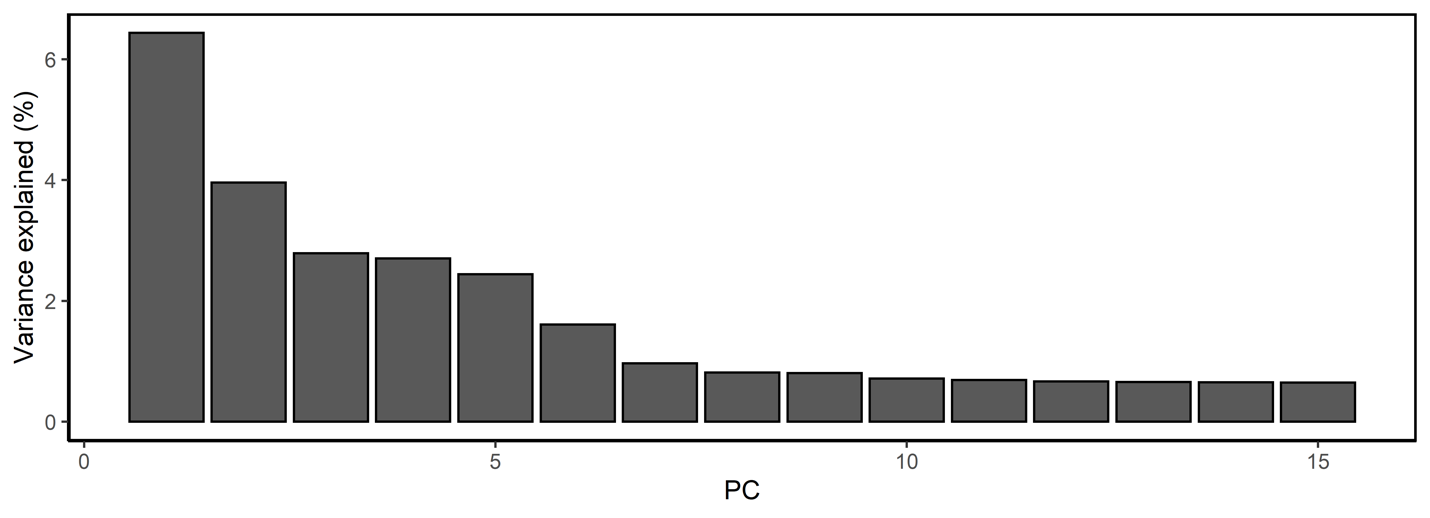


Figure S8.33 Scree plot of variance explained of the first 15 principal components from the PCA on the ALL dataset, subset to n ≤ 20 for introduced populations, as defined by popdef2, MAF > 0.1, and thinned to retain one SNP every 100,000 base pairs. The same samples were retained as the dataset used to create Figure 5A in the main text.


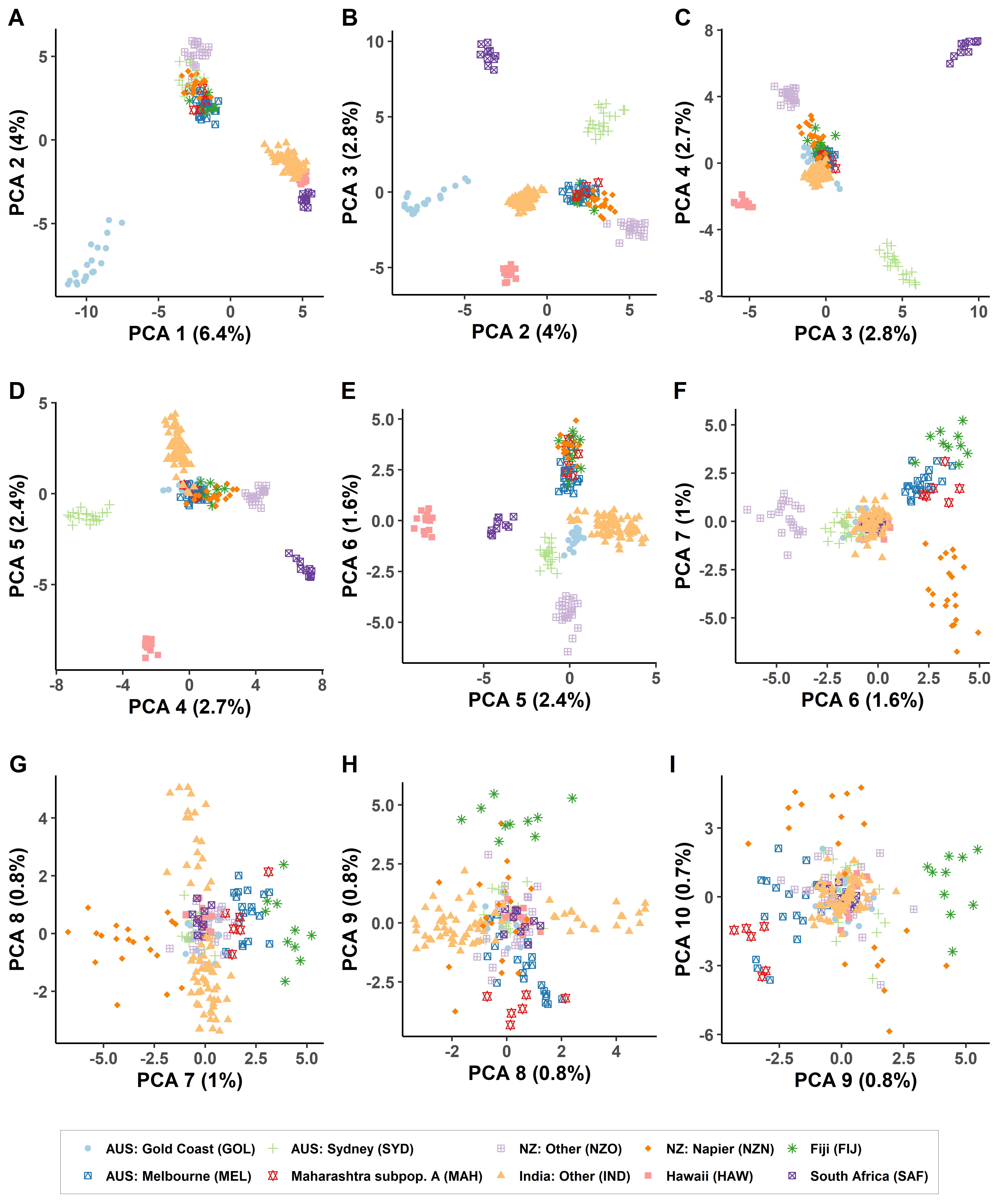


Figure S8.34 PCA plots of the ALL dataset, subset to n ≤ 20 for introduced populations, as defined by popdef2, MAF > 0.1, and thinned to retain one SNP every 100,000 base pairs. The same samples were retained as the dataset used to create Figure 5A in the main text. PCA A) 1 vs 2, B) 2 vs 3, C) 3 vs 4, D) 4 vs 5, E) 5 vs 6, F) 6 vs 7, G) 7 vs 8, H) 8 vs 9, and I) 9 vs 10. Samples are labelled based on popdef2.


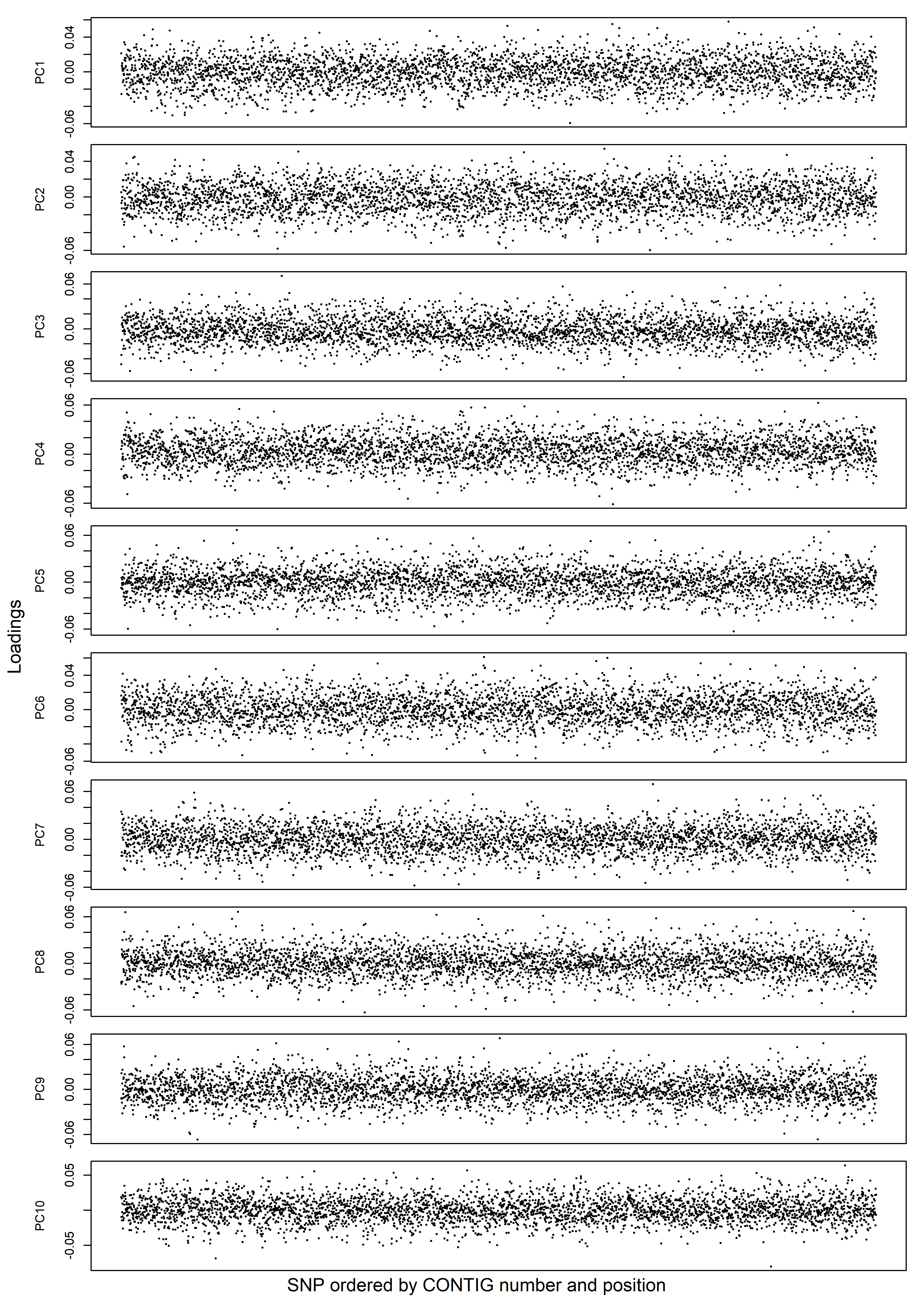


Figure S8.35 Loadings of PC1-10 from the PCA on the ALL dataset, subset to n ≤ 20 for introduced populations, as defined by popdef2, MAF > 0.1, and thinned to retain one SNP every 100,000 base pairs.


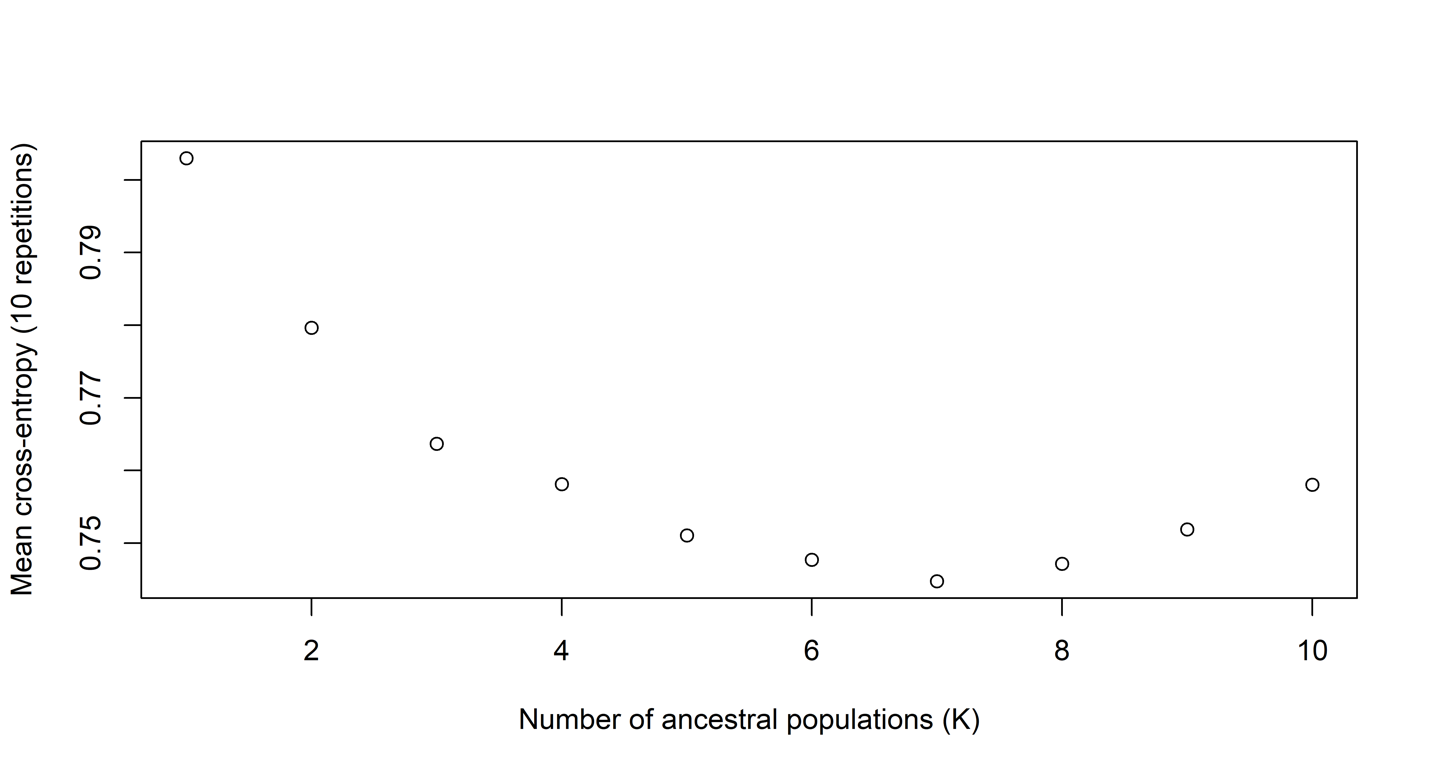


Figure S8.36 Mean cross-entropy plot of the sNMF analysis on the ALL dataset, subset to n ≤ 20 for introduced populations, as defined by popdef2, MAF > 0.1, and thinned to retain one SNP every 100,000 base pairs, averaged across 10 repetitions for each K value. Lowest cross-entropy value indicate best support of particular K value (K=7).


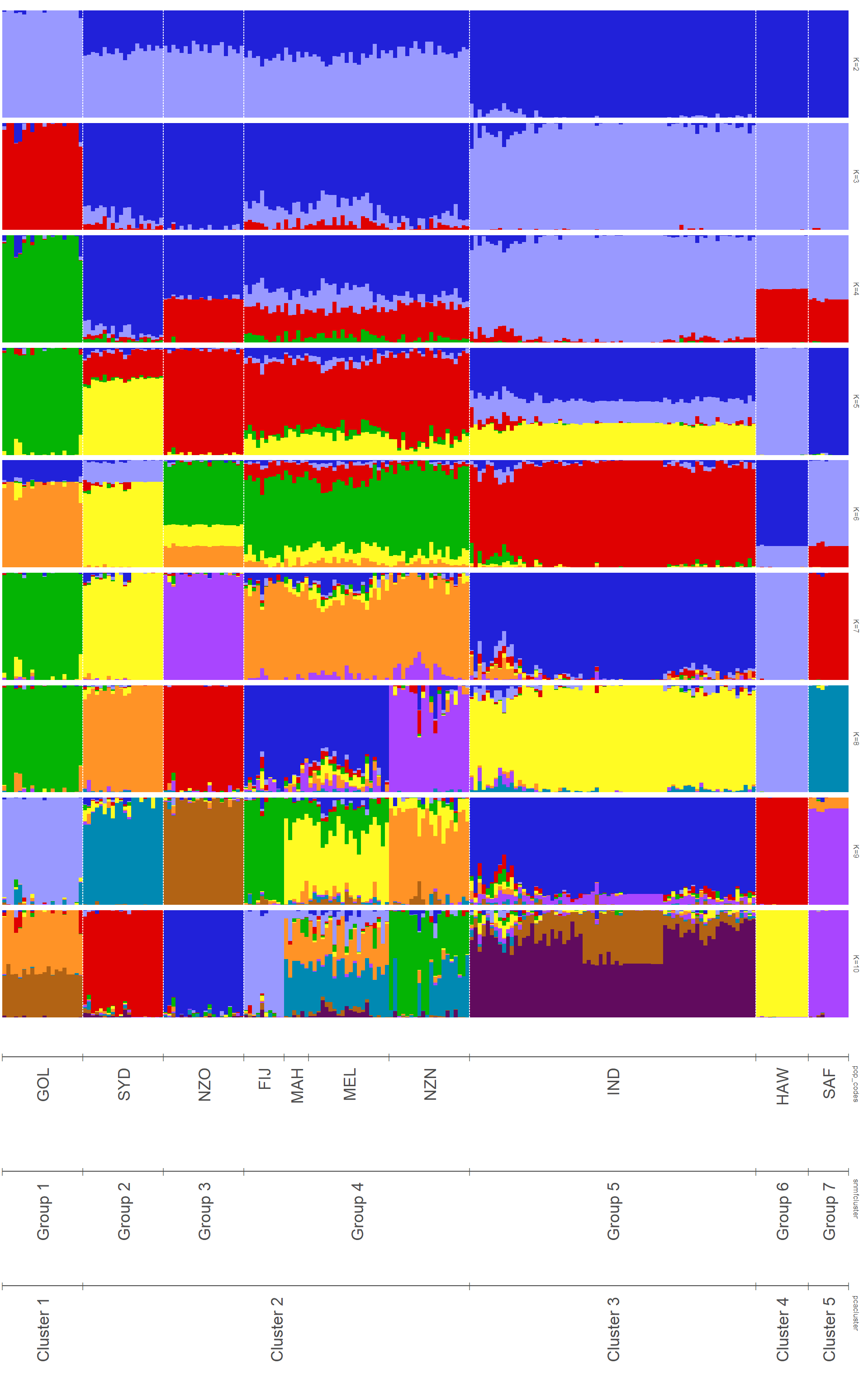


Figure S8.37 sNMF population structure plot for the ALL dataset, subset to n ≤ 20 for introduced populations, as defined by popdef2, MAF > 0.1, and thinned to retain one SNP every 100,000 base pairs, averaged across 10 repetitions for K = 2-10. Lowest mean cross-entropy value: K = 7.

# Appendix 9 – Genetic diversity indices

Table S 9.1 Genetic diversity indices in populations in the NZ dataset. Populations with fewer than 6 samples were removed. Populations were defined based on popdef1. H_o_ = mean observed heterozygosity, H_e_ = mean expected heterozygosity. *Calculated using the PopGenReport R package. **Calculated using the rarefaction method in HP-rare software with number of alleles = 12. Only SNPs with at least 80% call rates were retained for mean allelic richness, mean private allelic richness and median proportion of polymorphic calculations. Median proportion of polymorphic loci were calculated after subsampling populations to the smallest population size (Thames: n = 7) 100 times.

| **Population** | **H_o_** | **H_e_** | **Mean allelic richness** | | **Mean private allelic richness**** | **Median proportion of polymorphic loci** |
| --- | --- | --- | --- | --- | --- | --- |
|  |  |  | **R*** | **HP-rare**** |  |  |
| **Auckland (ROM)** | 0.206 | 0.198 | 1.594 | 1.621 | 0.003 | 0.648 |
| **Great Barrier Island** | 0.203 | 0.200 | 1.588 | 1.599 | 0.003 | 0.623 |
| **Hamilton (ROM)** | 0.212 | 0.198 | 1.593 | 1.627 | 0.003 | 0.652 |
| **Helensville** | 0.207 | 0.204 | 1.609 | 1.620 | 0.002 | 0.646 |
| **Leigh** | 0.207 | 0.206 | 1.617 | 1.623 | 0.003 | 0.648 |
| **Napier** | 0.210 | 0.204 | 1.621 | 1.655 | 0.027 | 0.685 |
| **Napier (ROM)** | 0.213 | 0.205 | 1.630 | 1.666 | 0.036 | 0.692 |
| **Ngunguru** | 0.206 | 0.206 | 1.615 | 1.621 | 0.002 | 0.646 |
| **Thames** | 0.208 | 0.194 | 1.580 | 1.626 | 0.003 | 0.654 |
| **Waiheke** | 0.207 | 0.196 | 1.587 | 1.615 | 0.002 | 0.635 |
| **Waitakeres** | 0.207 | 0.200 | 1.598 | 1.619 | 0.003 | 0.645 |

Table S 9.2 Genetic diversity indices in populations in the IND dataset. Populations with fewer than 6 samples were removed. Populations were defined based on popdef1. H_o_ = mean observed heterozygosity, H_e_ = mean expected heterozygosity. *Calculated using the PopGenReport R package. **Calculated using the rarefaction method in HP-rare software with number of alleles = 10. Only SNPs with at least 80% call rates were retained for mean allelic richness, mean private allelic richness and median proportion of polymorphic calculations. Median proportion of polymorphic loci were calculated after subsampling populations to the smallest population size (Maharahstra subpopulation A: n = 6) 100 times.

| **Population** | **H_o_** | **H_e_** | **Mean allelic richness** | | **Mean private allelic richness**** | **Median proportion of polymorphic loci** |
| --- | --- | --- | --- | --- | --- | --- |
|  |  |  | **R*** | **HP-rare**** |  |  |
| **Odisha** | 0.121 | 0.113 | 1.369 | 1.412 | 0.024 | 0.442 |
| **Andhra Pradesh** | 0.118 | 0.118 | 1.374 | 1.401 | 0.023 | 0.437 |
| **Maharashtra** | 0.120 | 0.114 | 1.366 | 1.409 | 0.025 | 0.433 |
| **Madhya Pradesh** | 0.119 | 0.116 | 1.361 | 1.395 | 0.021 | 0.431 |
| **West Bengal** | 0.119 | 0.115 | 1.361 | 1.396 | 0.021 | 0.430 |
| **Uttar Pradesh** | 0.117 | 0.116 | 1.364 | 1.391 | 0.019 | 0.428 |
| **Karnataka** | 0.118 | 0.116 | 1.366 | 1.392 | 0.024 | 0.422 |
| **Tamil Nadu** | 0.115 | 0.115 | 1.352 | 1.376 | 0.028 | 0.407 |
| **Maharashtra subpop. A** | 0.111 | 0.104 | 1.300 | 1.331 | 0.022 | 0.354 |

Table S 9.3 Genetic diversity indices in populations in the ALL dataset. Populations with fewer than 6 samples were removed. Populations were defined based on popdef1. H_o_ = mean observed heterozygosity, H_e_ = mean expected heterozygosity. *Calculated using the PopGenReport R package. **Calculated using the rarefaction method in HP-rare software with number of alleles = 10. Only SNPs with at least 80% call rates were retained for mean allelic richness, mean private allelic richness and median proportion of polymorphic calculations. Median proportion of polymorphic loci were calculated after subsampling populations to the smallest population size (Maharahstra subpopulation A: n = 6) 100 times.

| **Population** | **H_o_** | **H_e_** | **Mean allelic richness** | | **Mean private allelic richness**** | **Median proportion of polymorphic loci** |
| --- | --- | --- | --- | --- | --- | --- |
|  |  |  | **R*** | **HP-rare**** |  |  |
| **Odisha** | 0.106 | 0.099 | 1.325 | 1.363 | 0.017 | 0.393 |
| **Maharashtra** | 0.104 | 0.099 | 1.321 | 1.359 | 0.017 | 0.389 |
| **Andhra Pradesh** | 0.103 | 0.101 | 1.324 | 1.349 | 0.017 | 0.384 |
| **Uttar Pradesh** | 0.102 | 0.100 | 1.322 | 1.346 | 0.012 | 0.380 |
| **Madhya Pradesh** | 0.105 | 0.100 | 1.311 | 1.341 | 0.015 | 0.373 |
| **West Bengal** | 0.103 | 0.099 | 1.309 | 1.340 | 0.011 | 0.373 |
| **Karnataka** | 0.104 | 0.100 | 1.316 | 1.339 | 0.017 | 0.370 |
| **Tamil Nadu** | 0.100 | 0.098 | 1.308 | 1.329 | 0.022 | 0.357 |
| **Maharashtra subpop. A** | 0.094 | 0.088 | 1.262 | 1.291 | 0.001 | 0.313 |
| **Melbourne** | 0.098 | 0.096 | 1.286 | 1.289 | 0.002 | 0.310 |
| **Melbourne (ROM)** | 0.098 | 0.092 | 1.269 | 1.289 | 0.001 | 0.310 |
| **Fiji** | 0.095 | 0.089 | 1.261 | 1.275 | 0.001 | 0.295 |
| **Napier (ROM)** | 0.093 | 0.090 | 1.258 | 1.271 | 0.001 | 0.291 |
| **Napier** | 0.094 | 0.090 | 1.258 | 1.271 | 0.001 | 0.290 |
| **Thames** | 0.089 | 0.084 | 1.239 | 1.257 | 0.000 | 0.274 |
| **Auckland (ROM)** | 0.089 | 0.085 | 1.244 | 1.255 | 0.000 | 0.271 |
| **Hamilton (ROM)** | 0.091 | 0.086 | 1.242 | 1.255 | 0.000 | 0.271 |
| **Waitakeres** | 0.090 | 0.087 | 1.246 | 1.254 | 0.000 | 0.270 |
| **Ngunguru** | 0.090 | 0.089 | 1.250 | 1.253 | 0.000 | 0.269 |
| **Helensville** | 0.090 | 0.088 | 1.248 | 1.252 | 0.000 | 0.269 |
| **Leigh** | 0.089 | 0.089 | 1.250 | 1.252 | 0.000 | 0.267 |
| **Waiheke** | 0.090 | 0.085 | 1.240 | 1.250 | 0.000 | 0.265 |
| **Great Barrier Island** | 0.088 | 0.087 | 1.240 | 1.245 | 0.000 | 0.259 |
| **Hawaii** | 0.085 | 0.083 | 1.223 | 1.230 | 0.012 | 0.240 |
| **Sydney** | 0.085 | 0.083 | 1.222 | 1.224 | 0.002 | 0.235 |
| **Sydney (ROM)** | 0.081 | 0.081 | 1.211 | 1.216 | 0.002 | 0.226 |
| **South Africa** | 0.077 | 0.074 | 1.188 | 1.195 | 0.013 | 0.202 |
| **Gold Coast** | 0.071 | 0.070 | 1.182 | 1.184 | 0.000 | 0.193 |


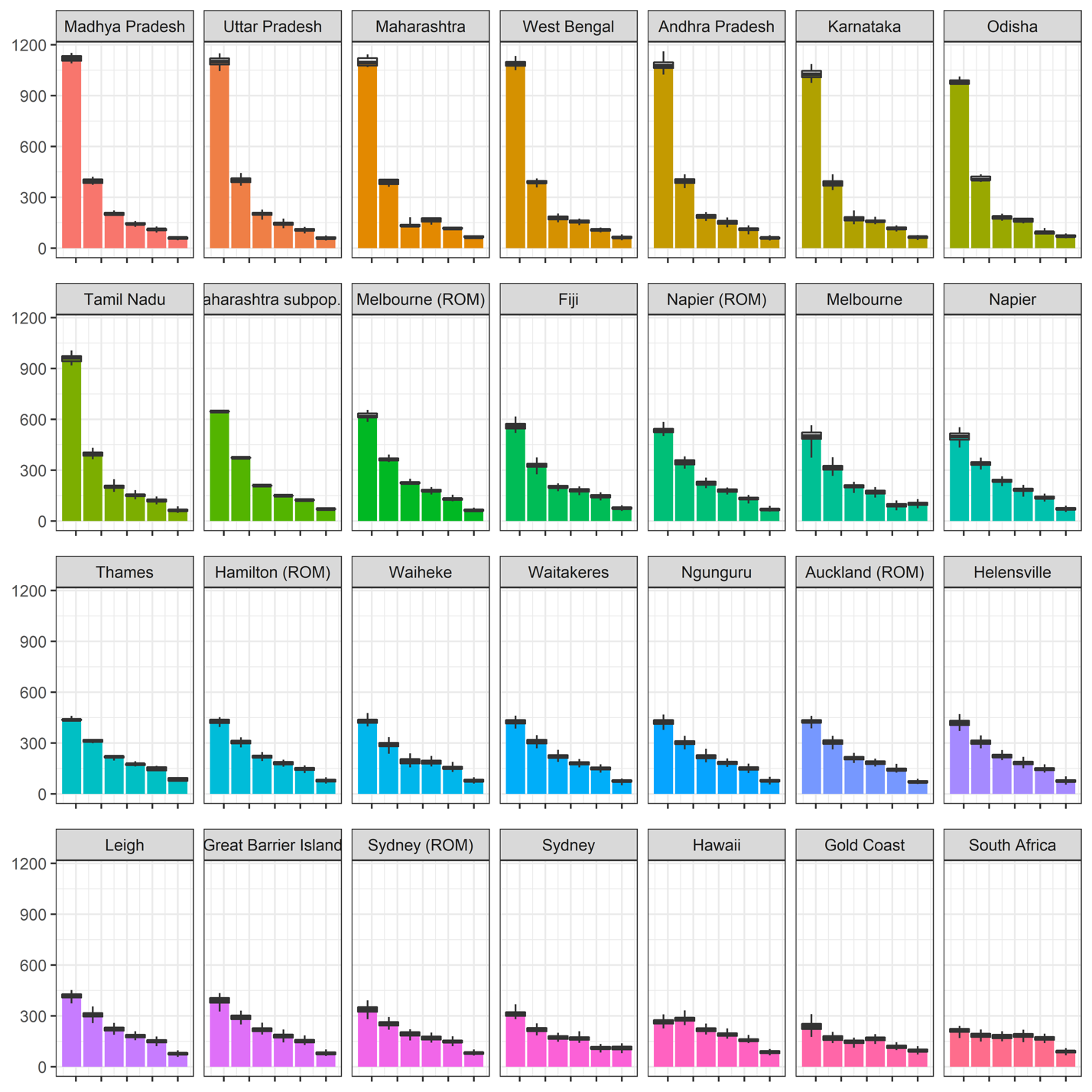


Figure S9.1 SFS of the ALL dataset. Populations with fewer than 6 samples were removed. Populations were defined based on popdef1.

# Appendix 10 – PCA and sNMF from other SNP calling pipelines

## STACKS NZ dataset


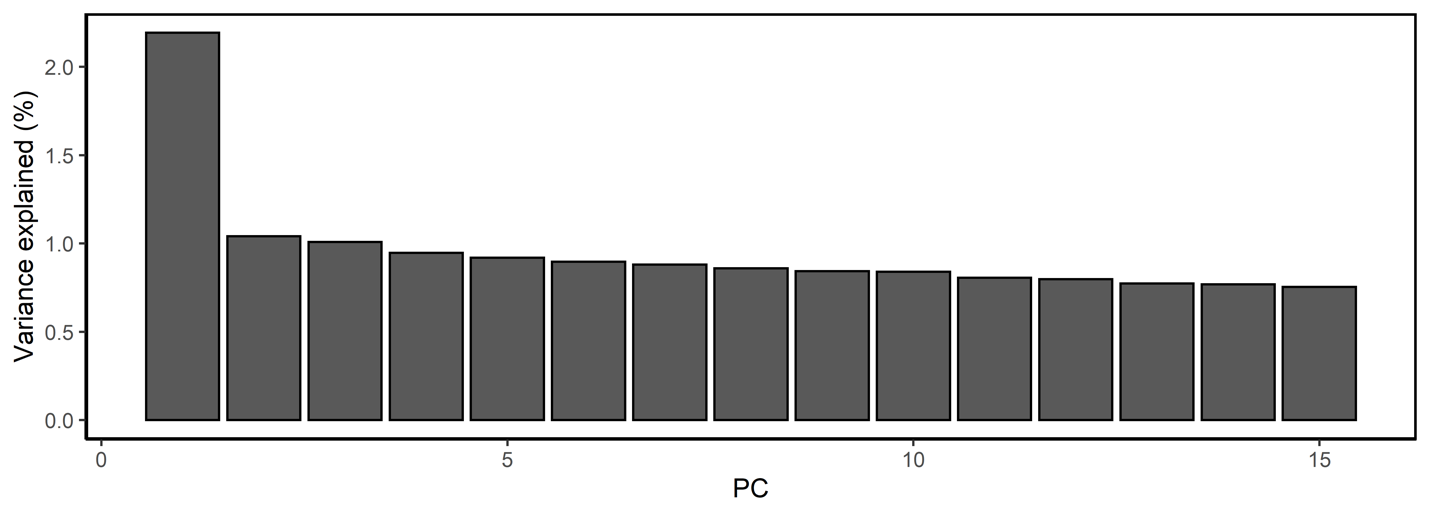


Figure S10.1

Scree plot of variance explained of the first 15 principal components from the PCA on the STACKS NZ dataset.


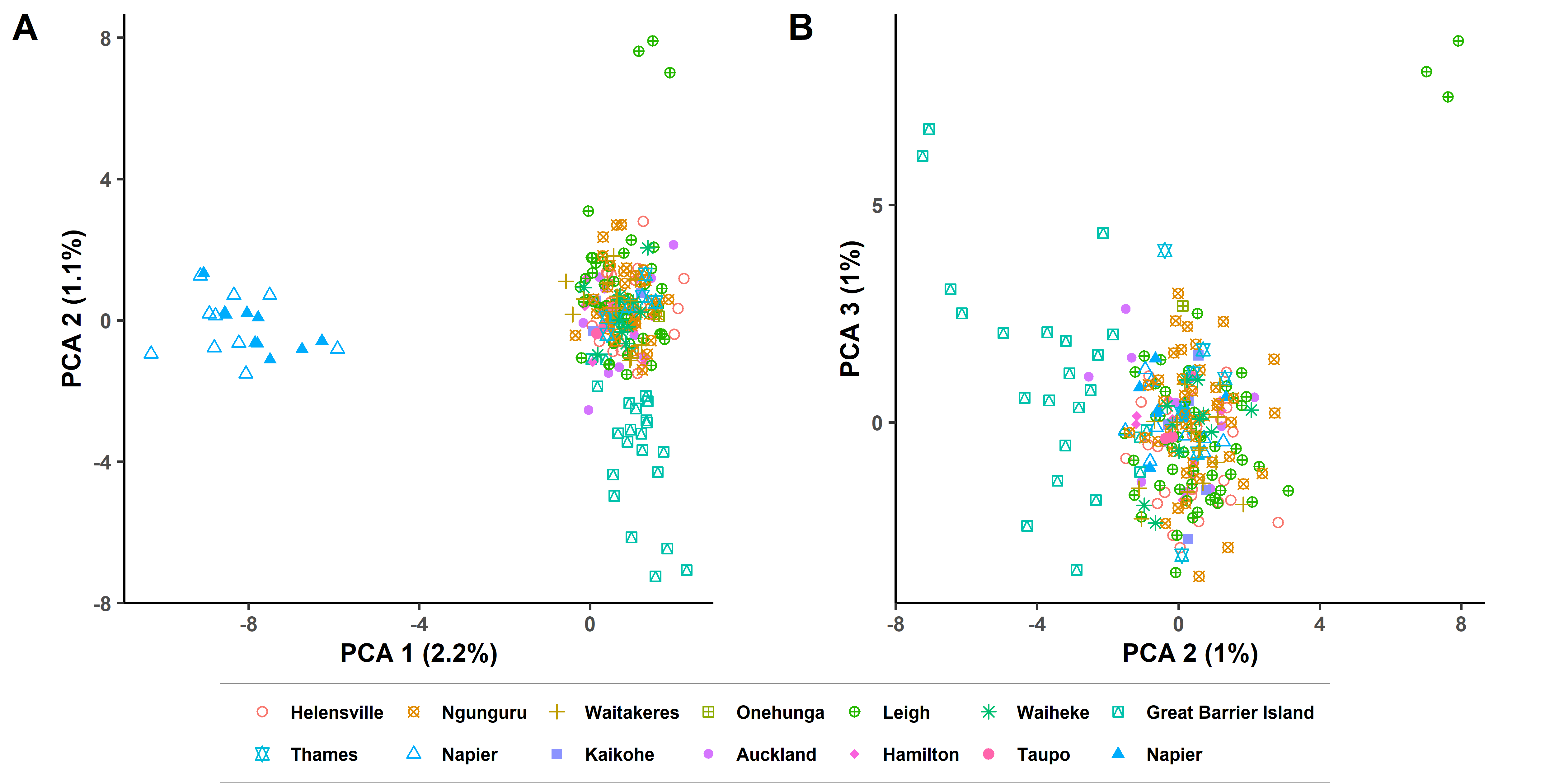


Figure S10.2 PCA plots of the STACKS NZ dataset. A) PCA1 vs PCA2, and B) PCA2 vs PCA3 of the NZ dataset. Filled shapes represent data from ROM.


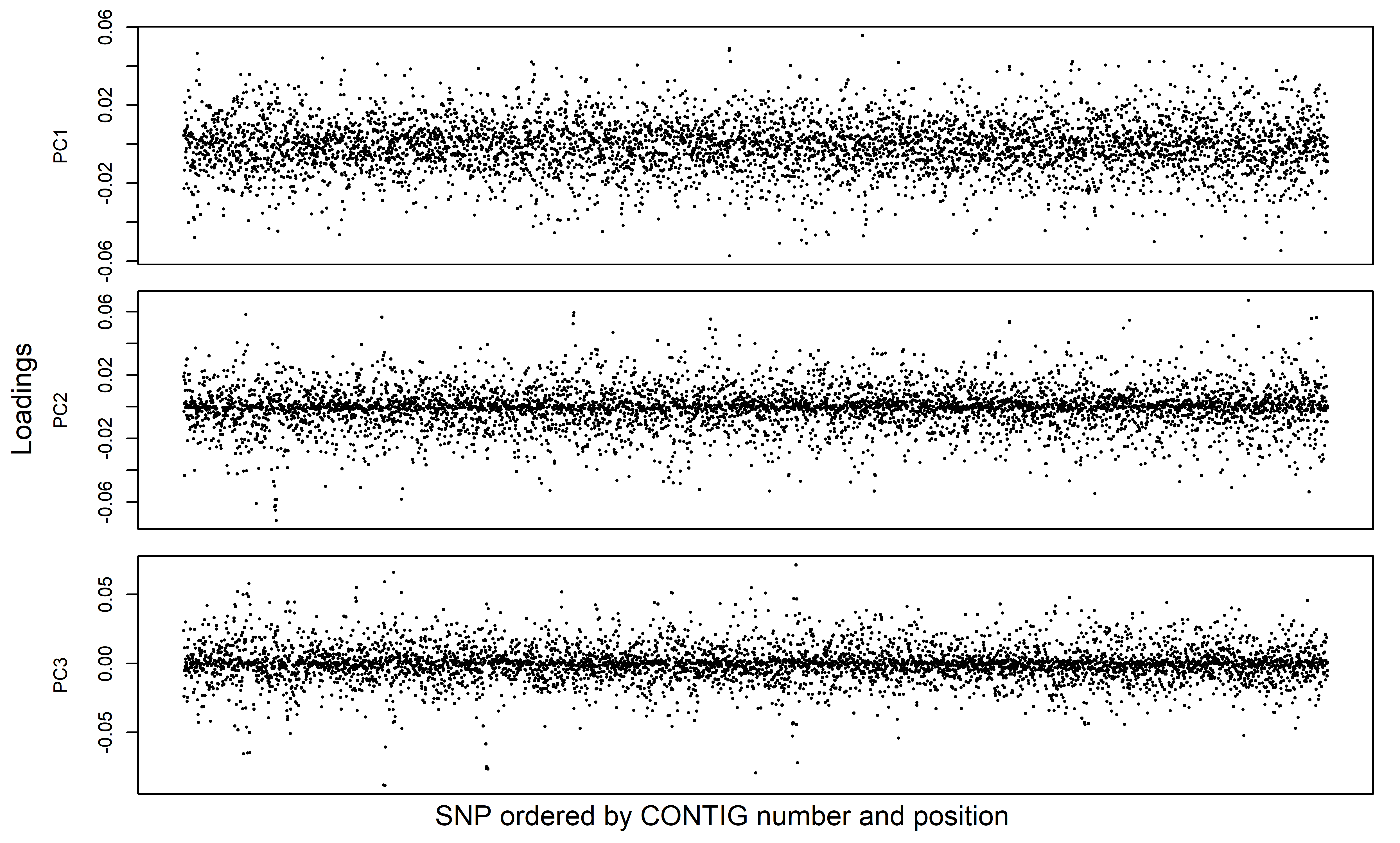


Figure S10.3 Loadings of PC1-3 for PCA on the STACKS NZ dataset.


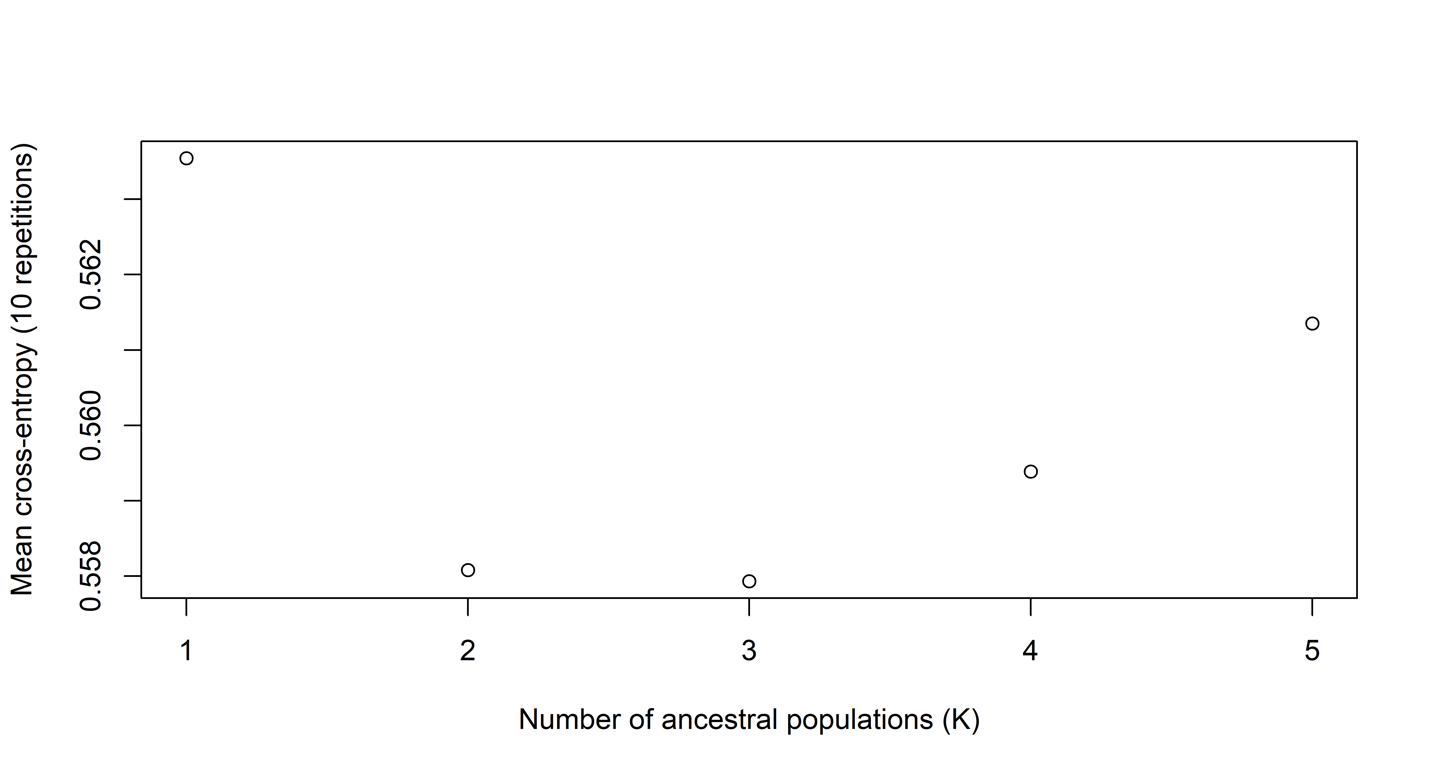


Figure S10.4 Mean cross-entropy plot of sNMF analysis on the STACKS NZ dataset, averaged across 10 repetitions for each K value. Lowest cross-entropy value indicate best support of particular K value.


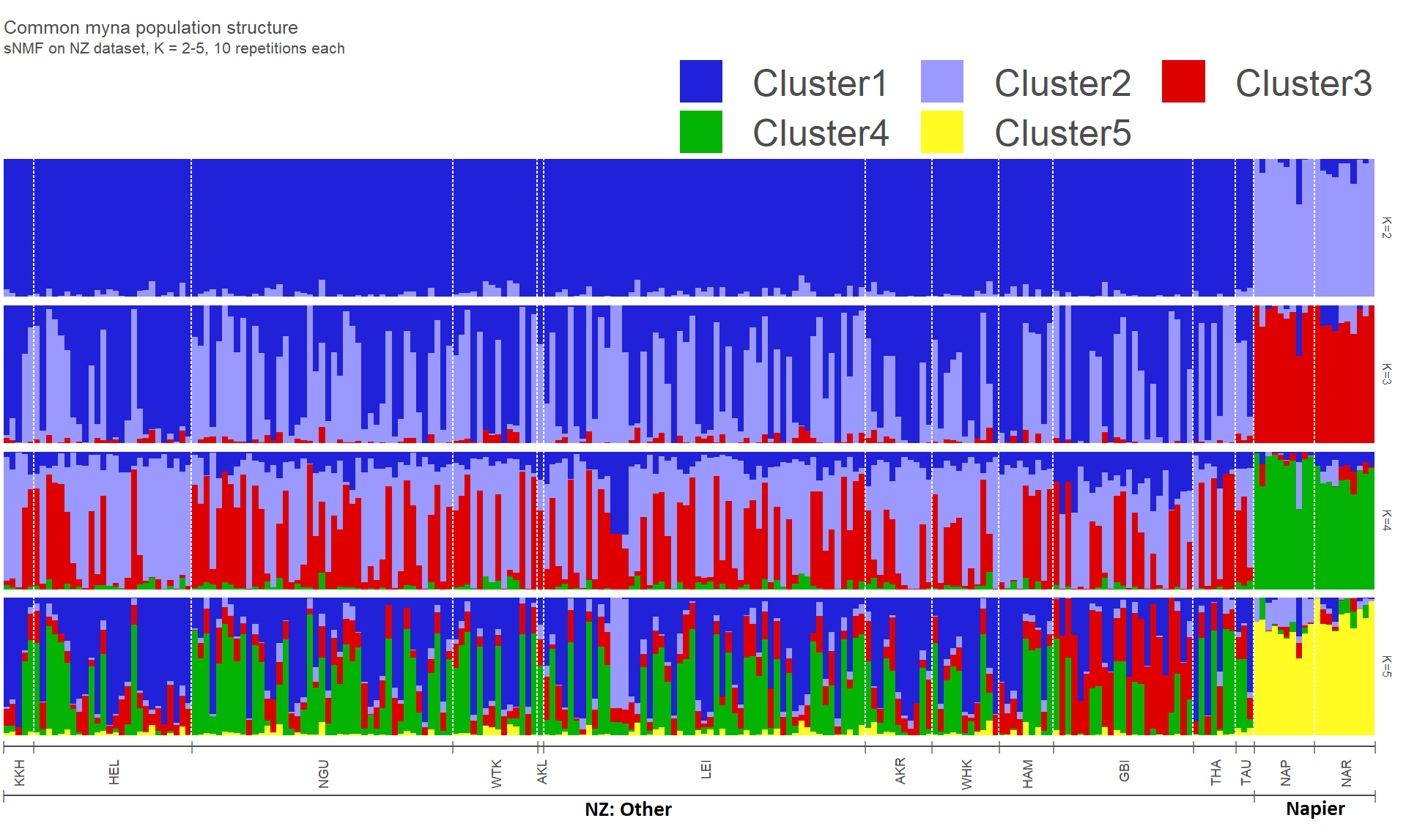


Figure S10.5 sNMF population structure plot for the STACKS NZ dataset, k = 2-5, average of 10 repetitions. Three-letter codes labelled beneath the figure refers to the labels in Figure 2 in the main text. KKH = Kaikohe (ROM), HEL = Helensville, NGU = Ngunguru, WTK = Waitakeres, AKL = Auckland, LEI = Leigh, AKR = Auckland (ROM), WHK = Waiheke, HAM = Hamilton (ROM), GBI = Great Barrier Island, THA = Thames, TAU = Taupo (ROM), NAP = Napier, NAR = Napier (ROM)

## STACKS IND dataset


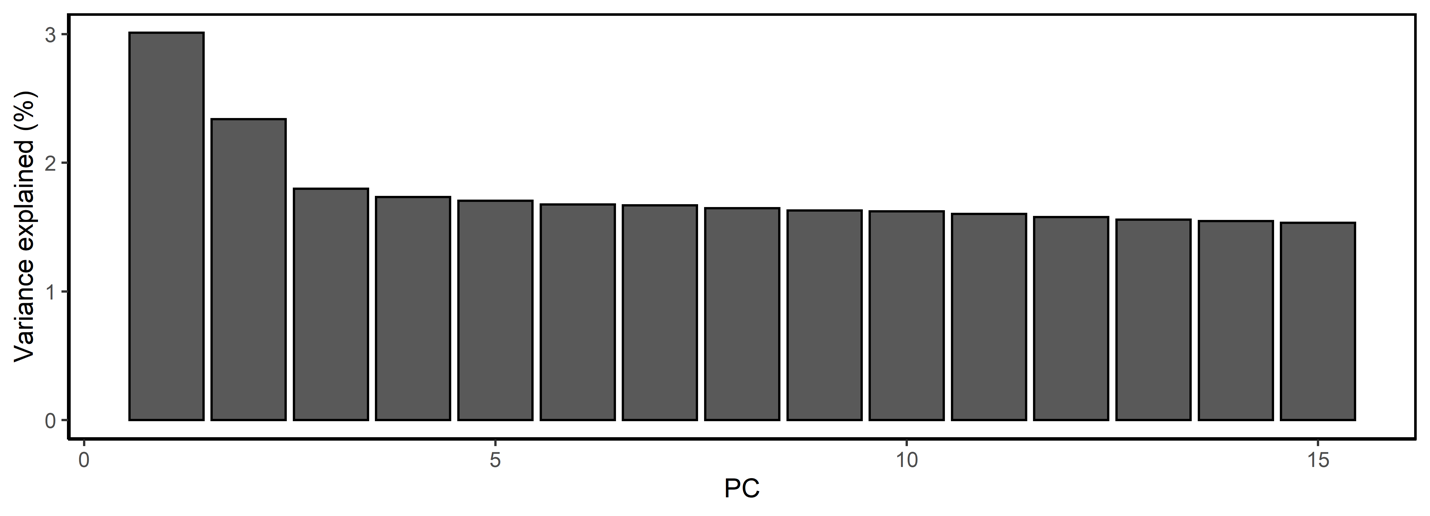


Figure S10.6 Scree plot of variance explained of the first 15 principal components from the PCA on the STACKS IND dataset.


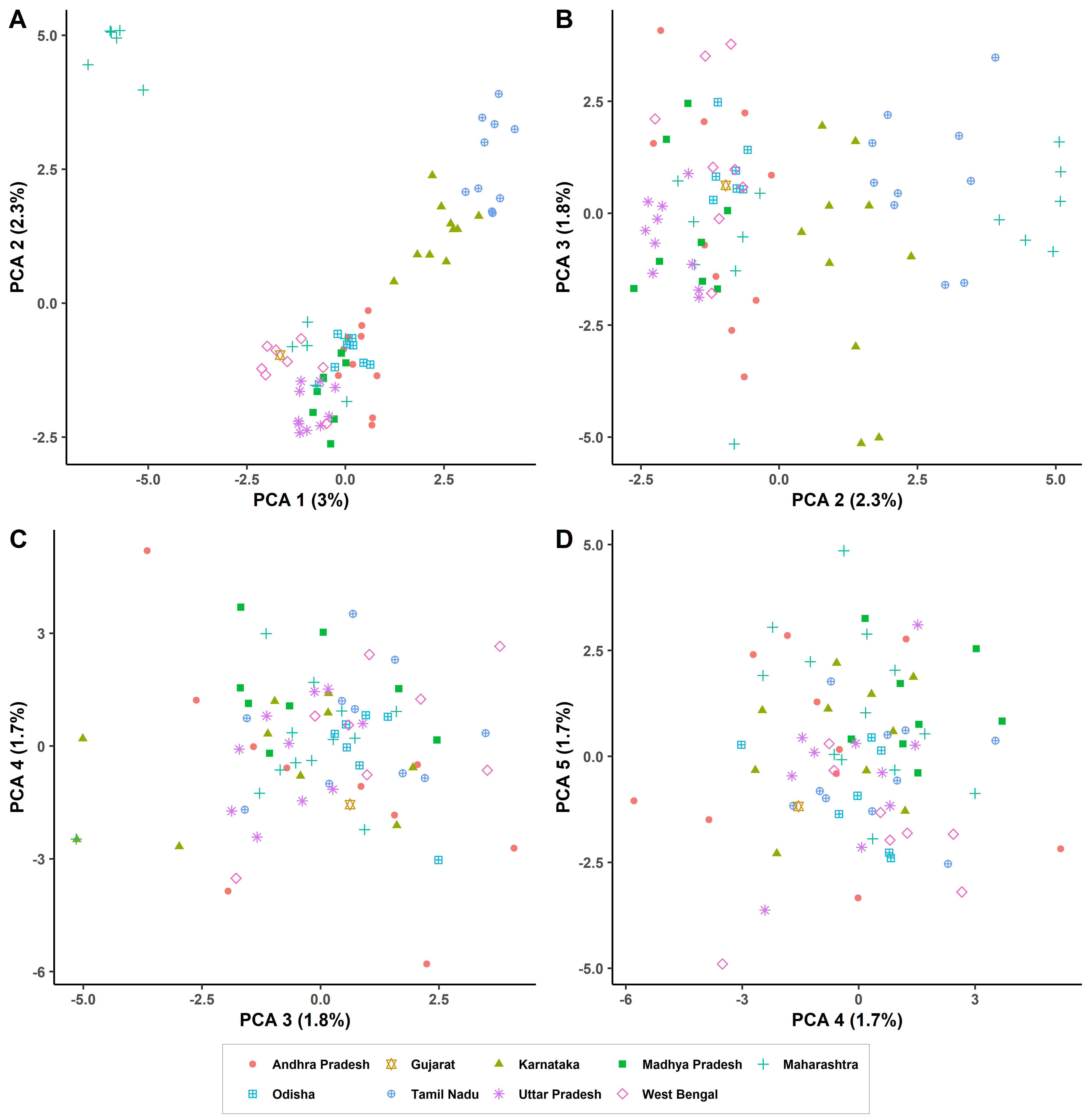


Figure S10.7 PCA plots of the STACKS IND dataset. A) PCA1 vs 2, B) PCA2 vs 3, C) PCA3 vs 4, and D) PCA4 vs 5. Samples are labelled based on location sampled. Note that six samples from Maharashtra cluster separately in PCA1 vs 2, corresponding to Maharashtra subpopulation A which cluster with Melbourne, Fiji, and Napier.


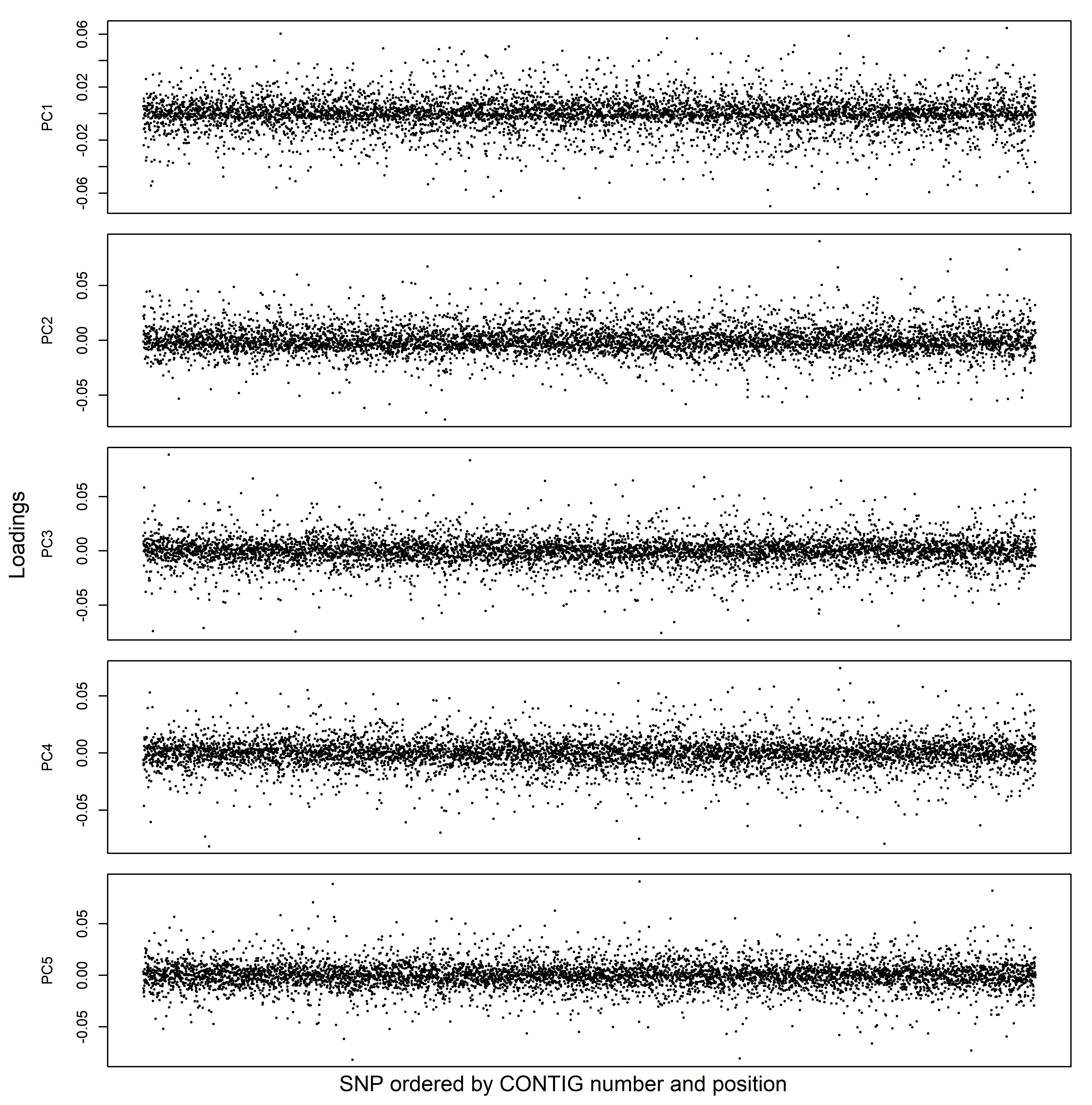


Figure S10.8 Loadings of PC1-5 from PCA on the STACKS IND dataset.


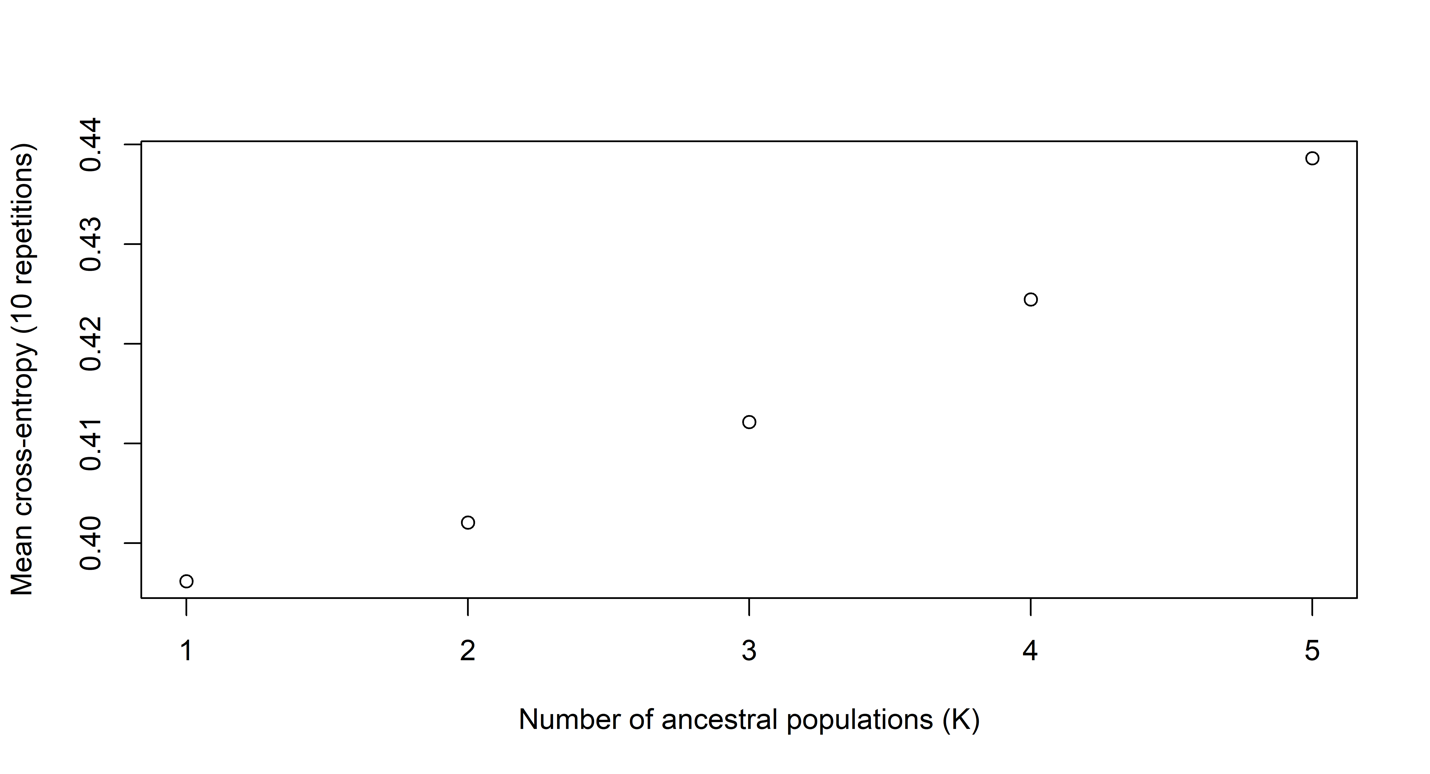


Figure S10.9 Mean cross-entropy plots of 10 repetitions at k = 1-5 of sNMF analysis on the STACKS IND dataset.


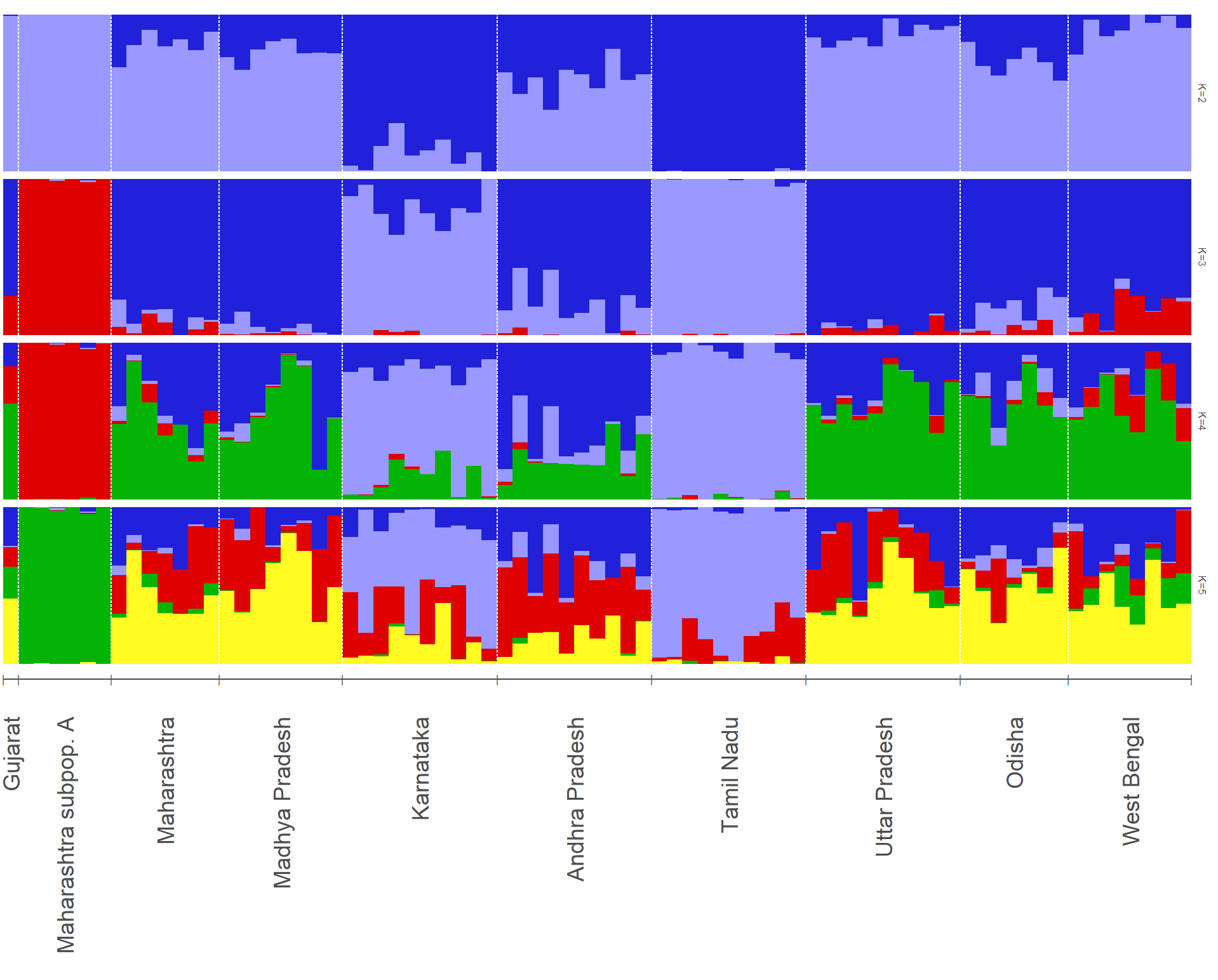


Figure S10.10 sNMF population structure plot for the STACKS IND dataset, k = 2-5, average of 10 repetitions. Populations are defined based on popdef1 population definition.

## STACKS ALL dataset

As with the BCFtools dataset that was shown in the main text, PCA was performed on the subsampled dataset with no more than 20 individuals per introduced populations. sNMF was performed on the full dataset.


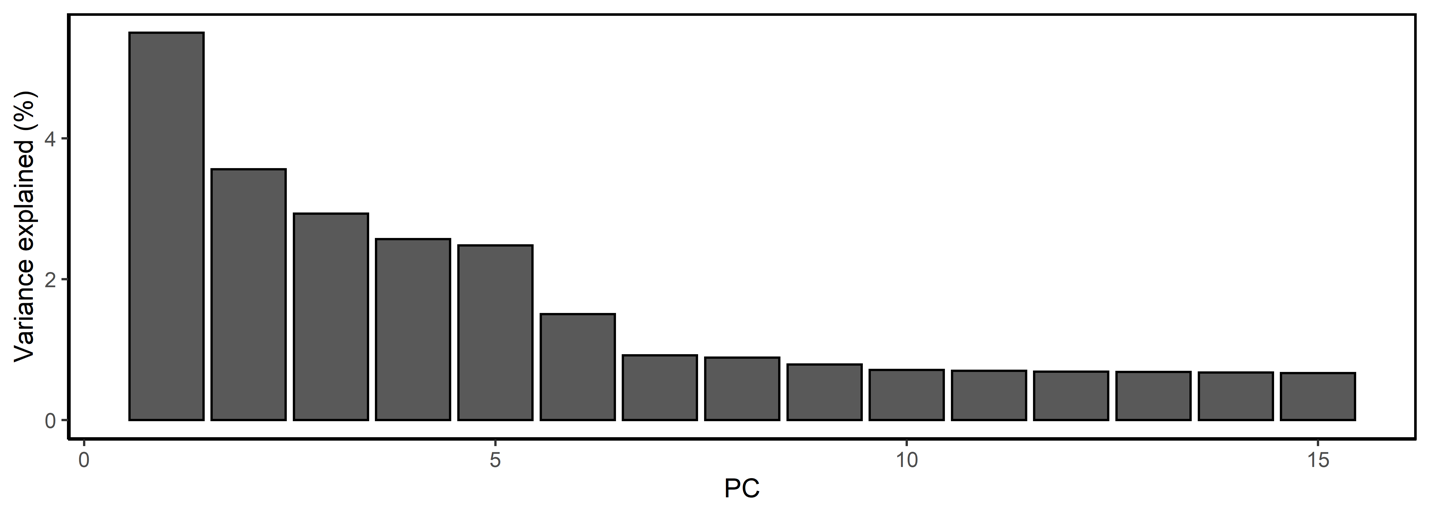


Figure S10.11 Scree plot of variance explained of the first 15 principal components from the PCA on the STACKS ALL dataset (subset to n ≤ 20 for introduced populations, as defined by popdef2).


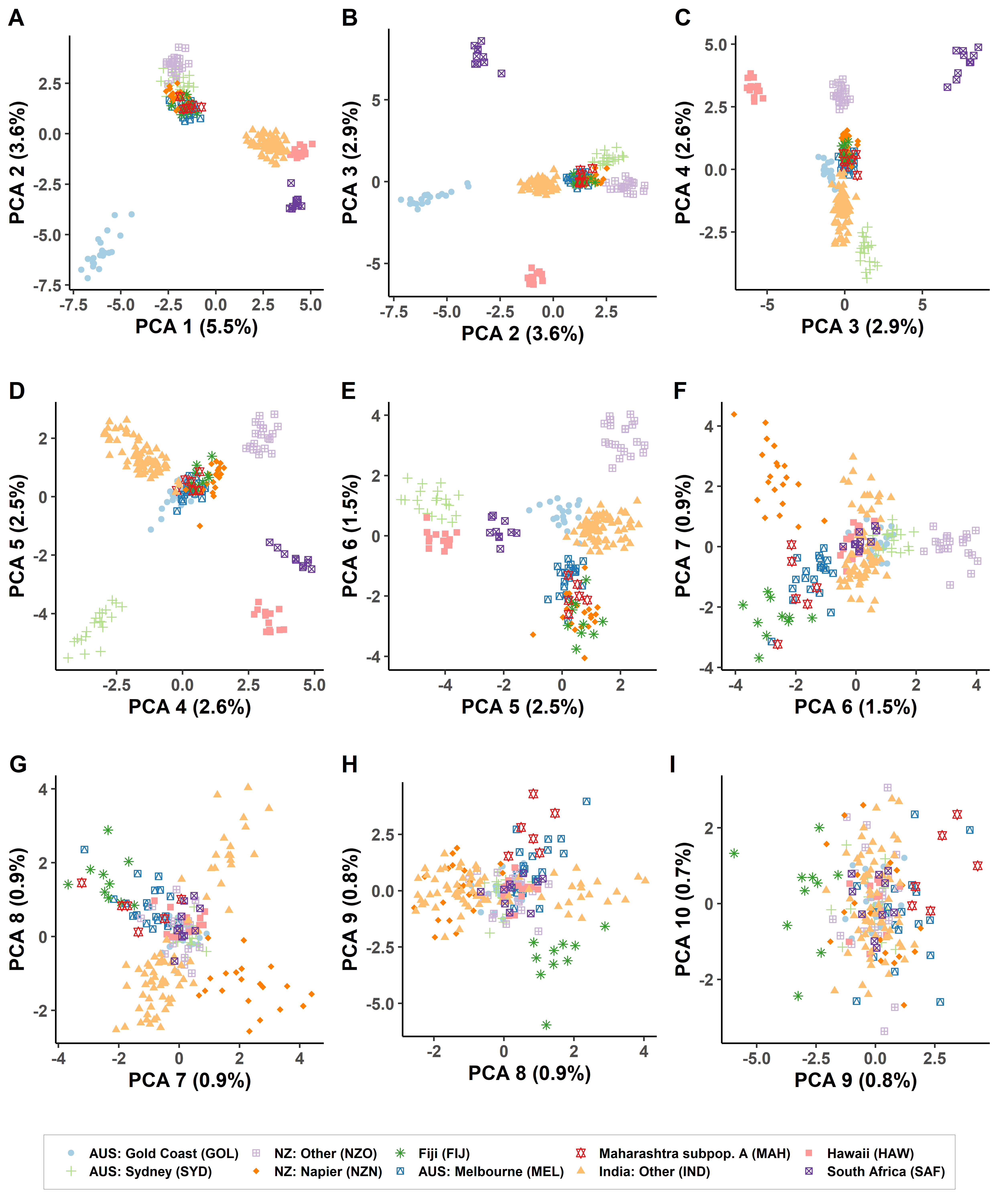


Figure S10.12 PCA plots of the ALL dataset. PCA A) 1 vs 2, B) 2 vs 3, C) 3 vs 4, D) 4 vs 5, E) 5 vs 6, F) 6 vs 7, G) 7 vs 8, H) 8 vs 9, and I) 9 vs 10. Samples are labelled based on popdef2, same as in Figure 5A in the main text.


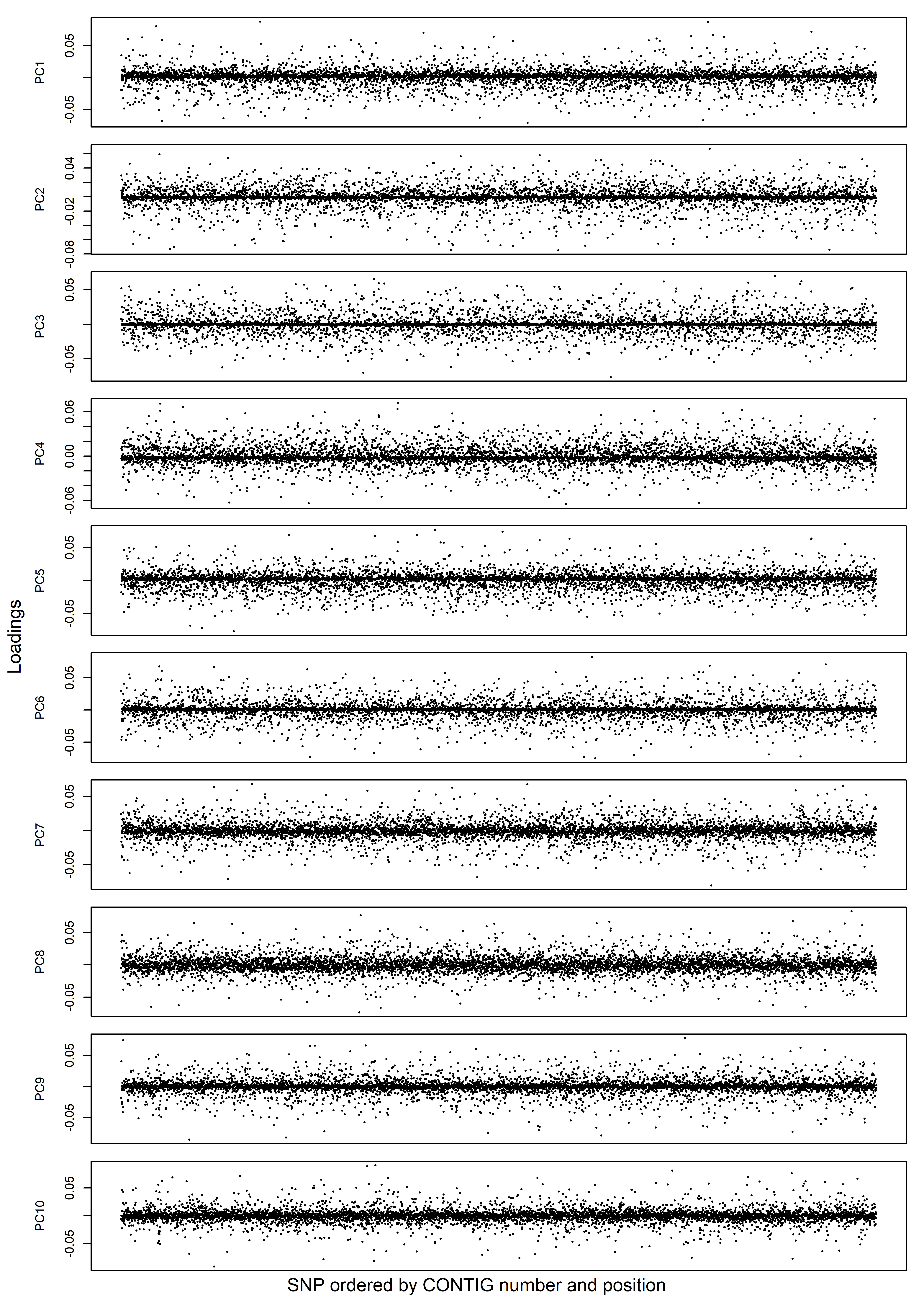


Figure S10.13 Loadings of PC1-10 from the PCA on the STACKS ALL dataset.


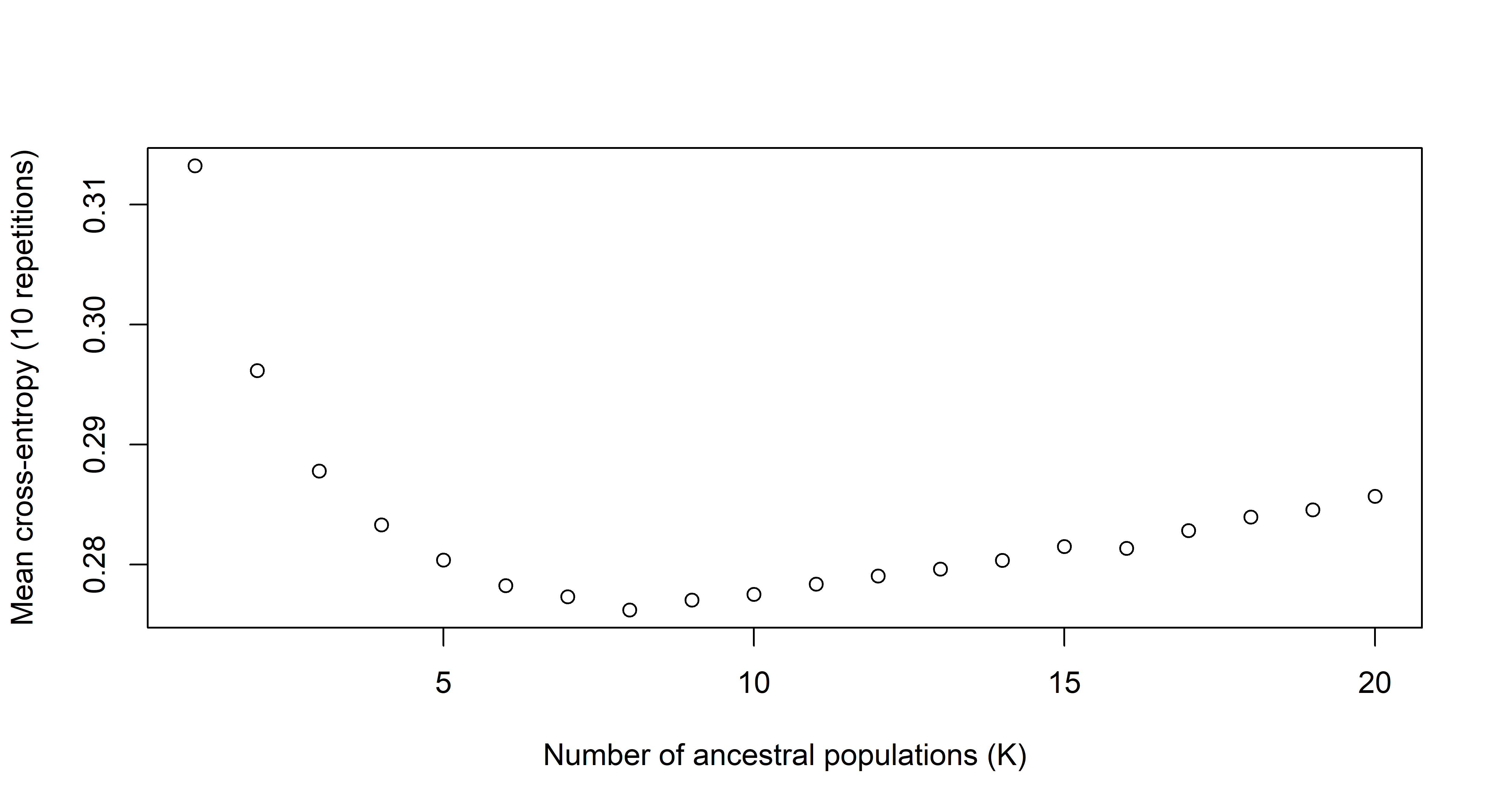


Figure S10.14 Mean cross-entropy plots of 10 repetitions at k = 1-20 of sNMF analysis on the STACKS ALL dataset.


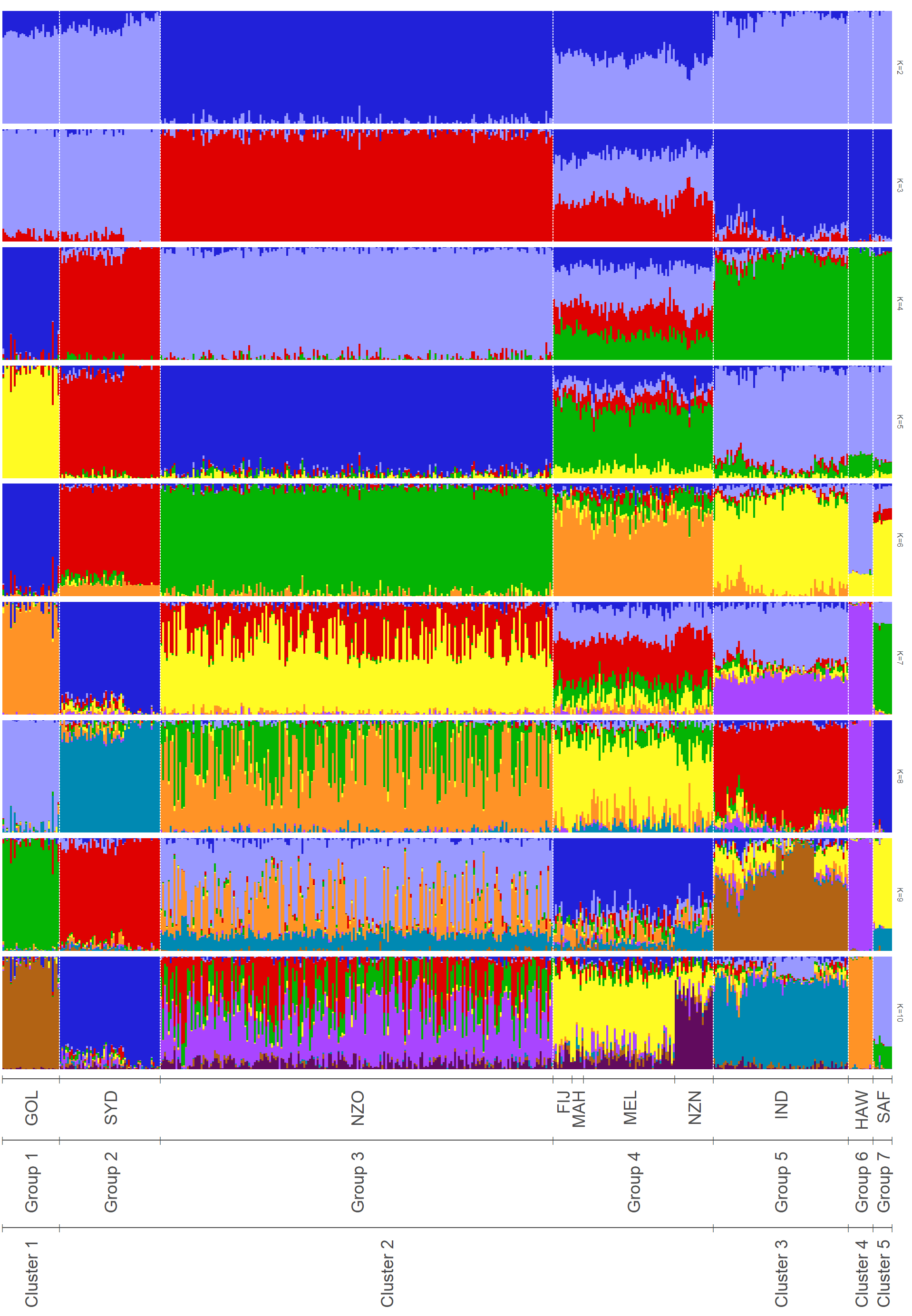


Figure S10.15 sNMF population structure plot for the STACKS ALL dataset. The labels correspond to labels in Figure 5 of the main text.

## DART NZ dataset


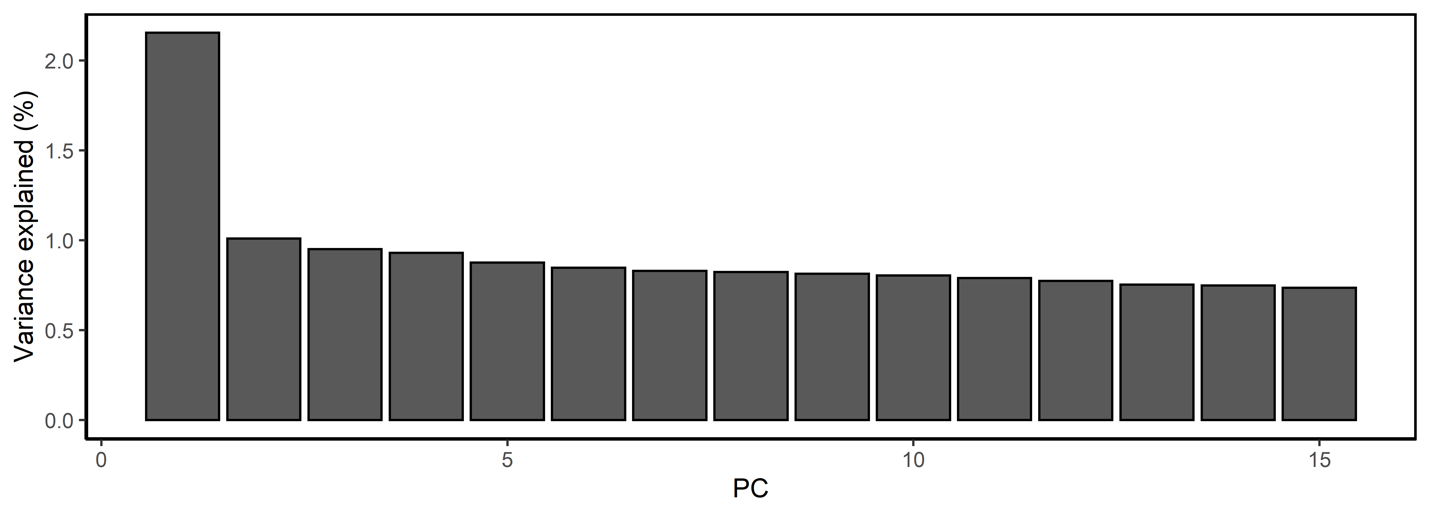


Figure S10.16 Scree plot of variance explained of the first 15 principal components from the PCA on the STACKS NZ dataset.


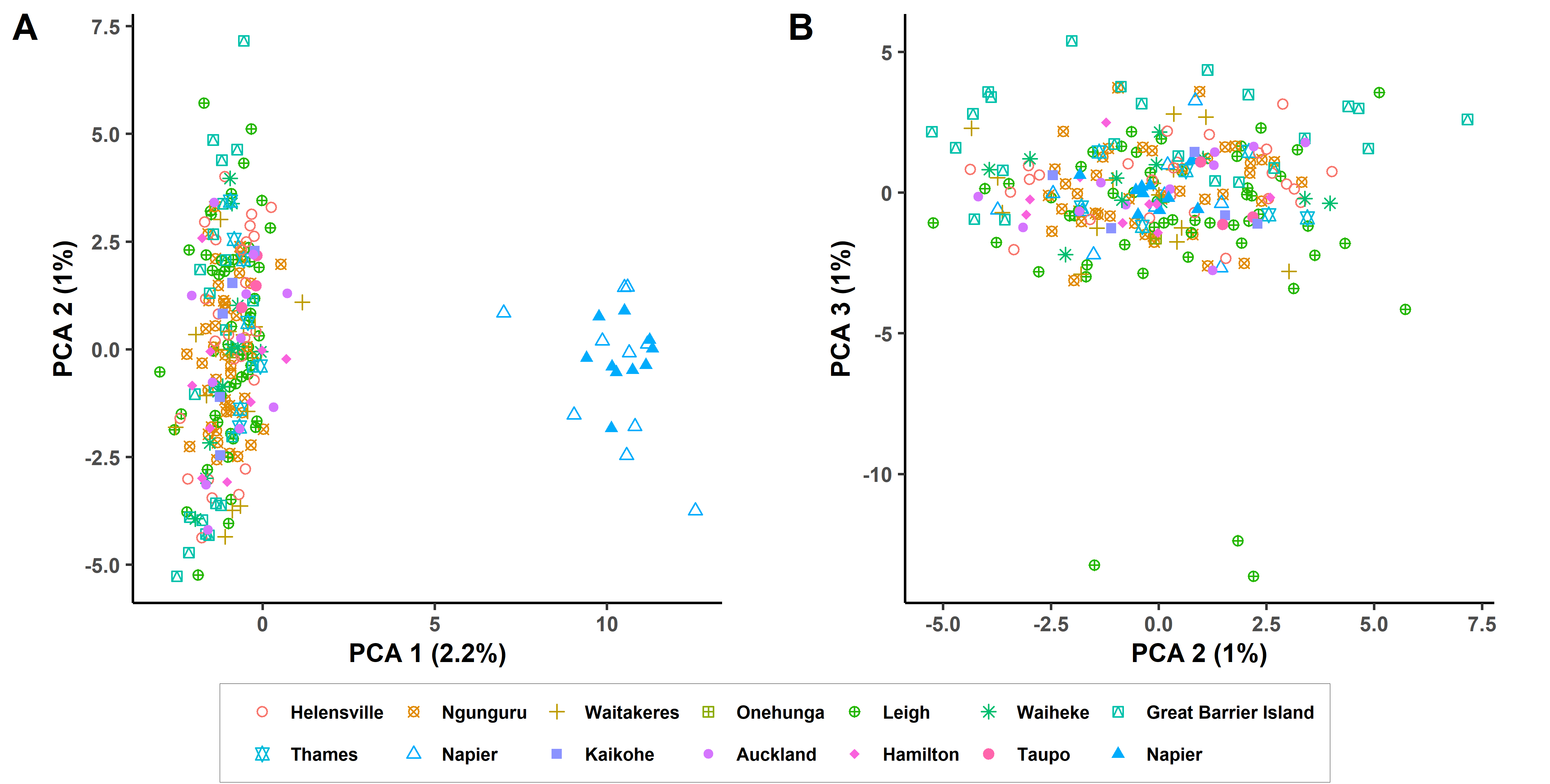


Figure S10.17 PCA plots of the DART IND dataset. A) PCA1 vs 2, B) PCA2 vs 3, C) PCA3 vs 4, and D) PCA4 vs 5. Samples are labelled based on location sampled. Note that six samples from Maharashtra cluster separately in PCA1 vs 2, corresponding to Maharashtra subpopulation A which cluster with Melbourne, Fiji, and Napier.


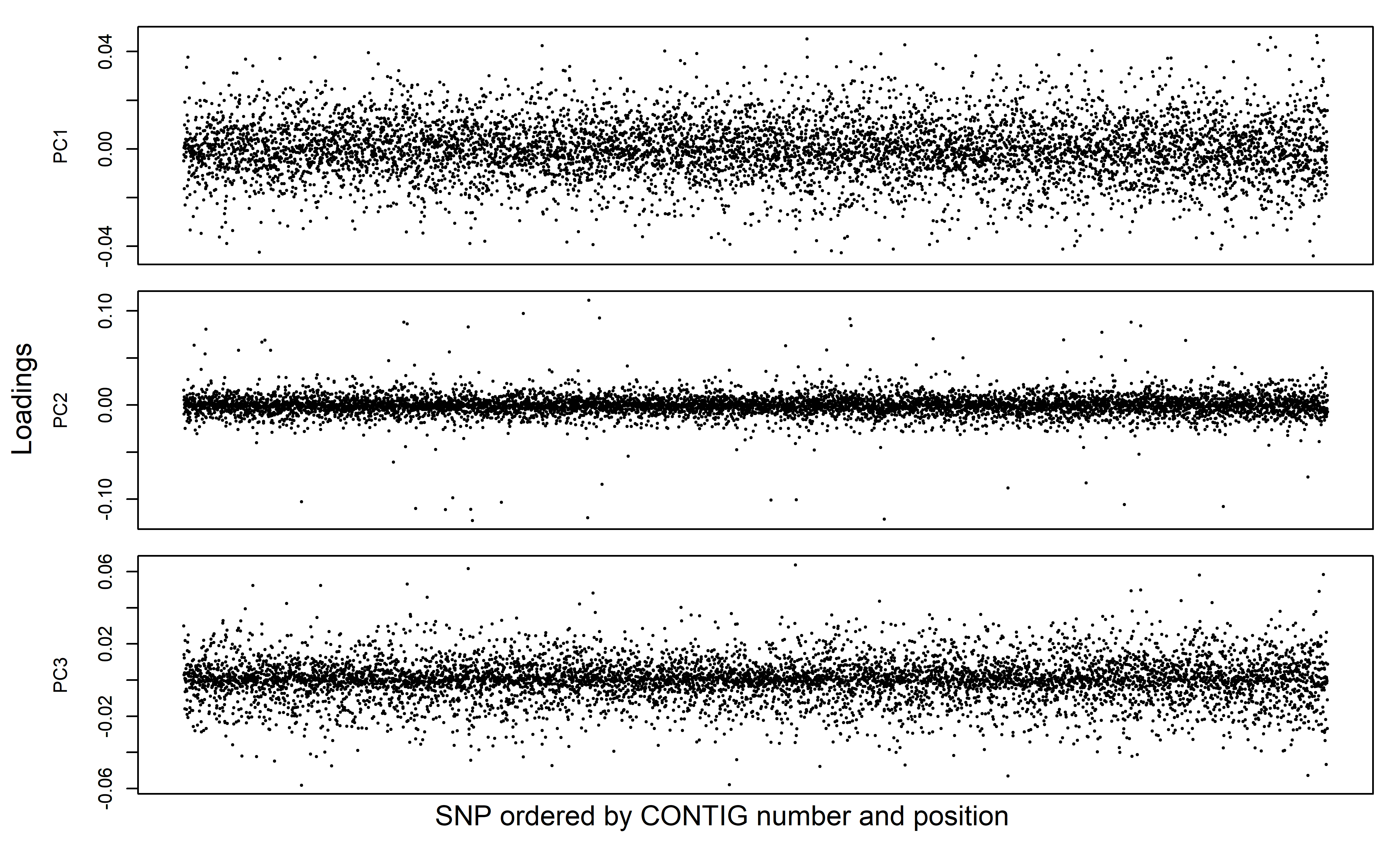


Figure S10.18 Loadings of PC1-3 for PCA on the DART NZ dataset.


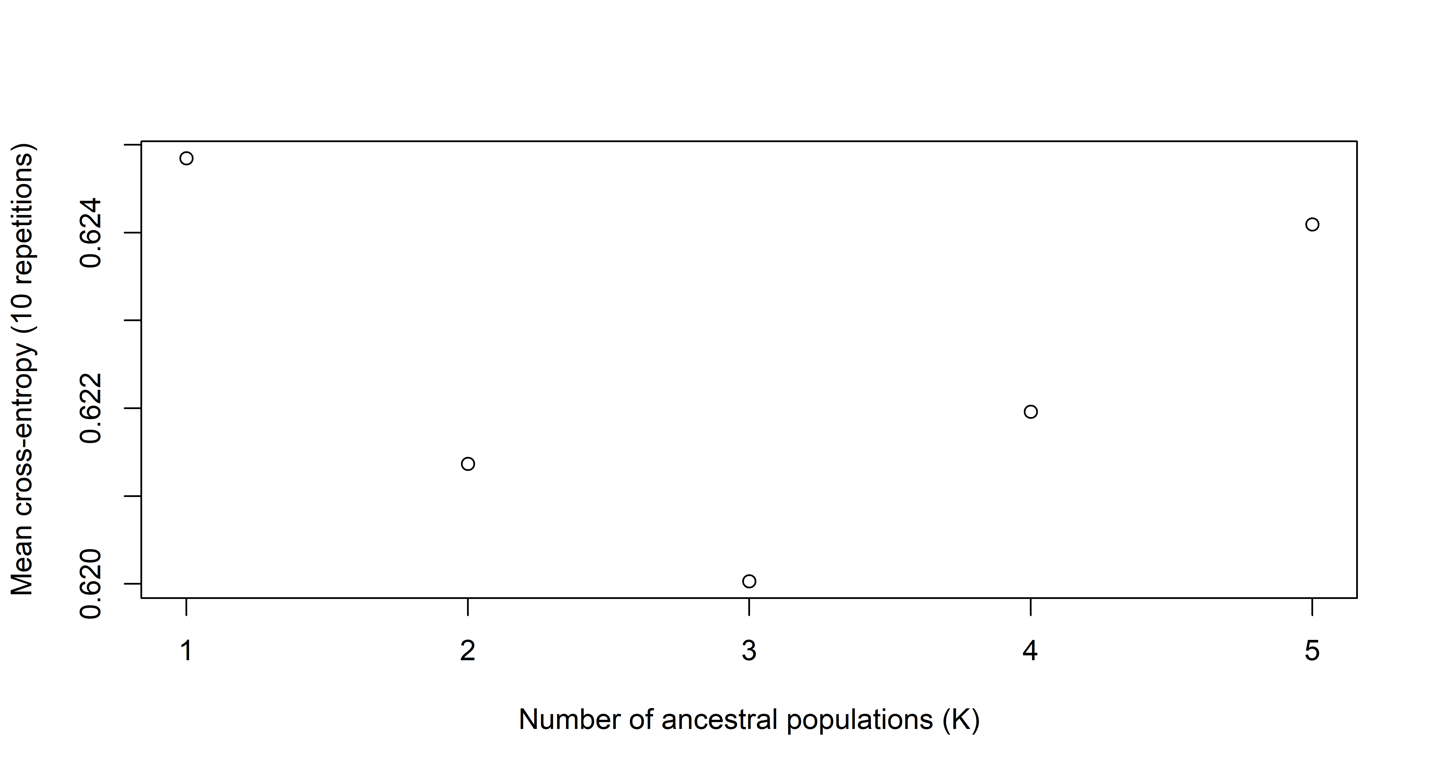


Figure S10.19 Mean cross-entropy plot of sNMF analysis on the DART NZ dataset, averaged across 10 repetitions for each K value. Lowest cross-entropy value indicate best support of particular K value.


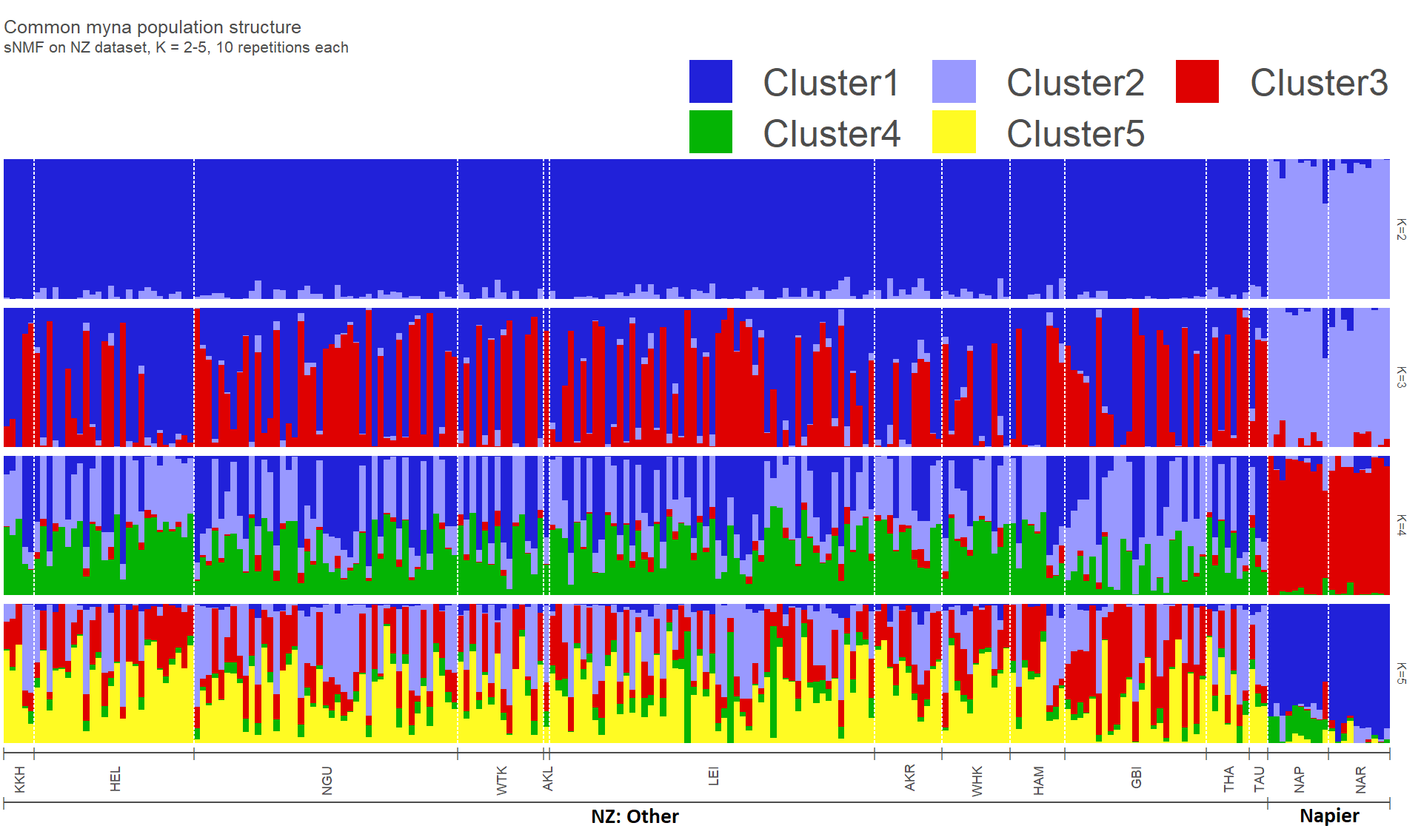


Figure S10.20 sNMF population structure plot for the DART NZ dataset, k = 2-5, average of 10 repetitions. Three-letter codes labelled beneath the figure refers to the labels in Figure 2 in the main text. KKH = Kaikohe (ROM), HEL = Helensville, NGU = Ngunguru, WTK = Waitakeres, AKL = Auckland, LEI = Leigh, AKR = Auckland (ROM), WHK = Waiheke, HAM = Hamilton (ROM), GBI = Great Barrier Island, THA = Thames, TAU = Taupo (ROM), NAP = Napier, NAR = Napier (ROM)

## DART IND dataset


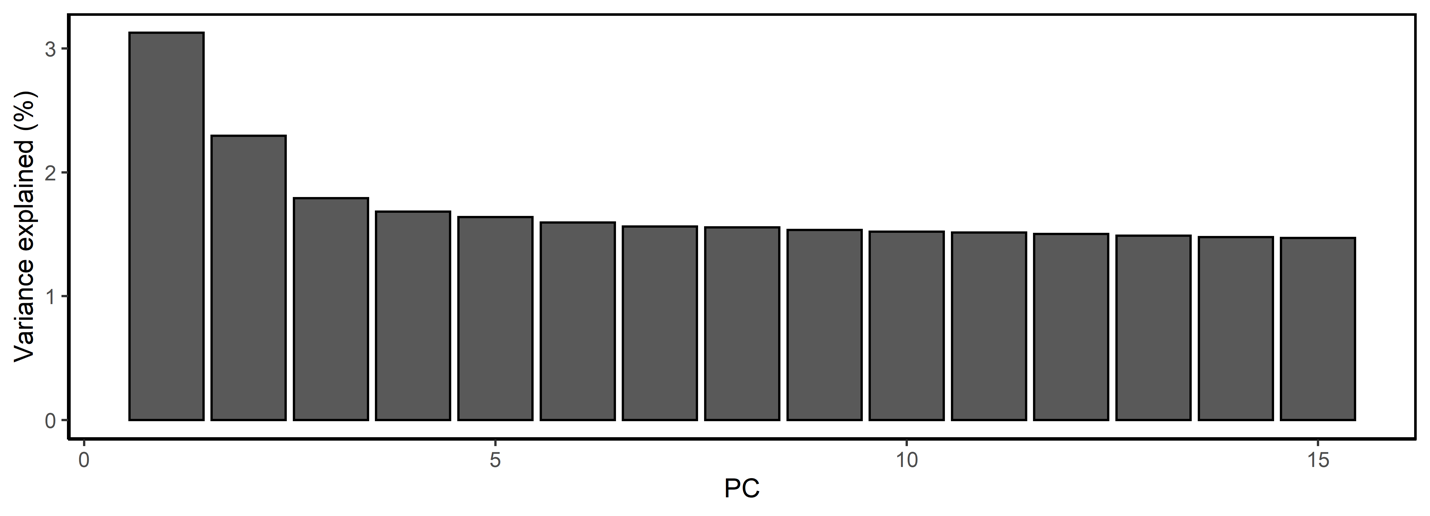


Figure S10.21 Scree plot of variance explained of the first 15 principal components from the PCA on the DART IND dataset


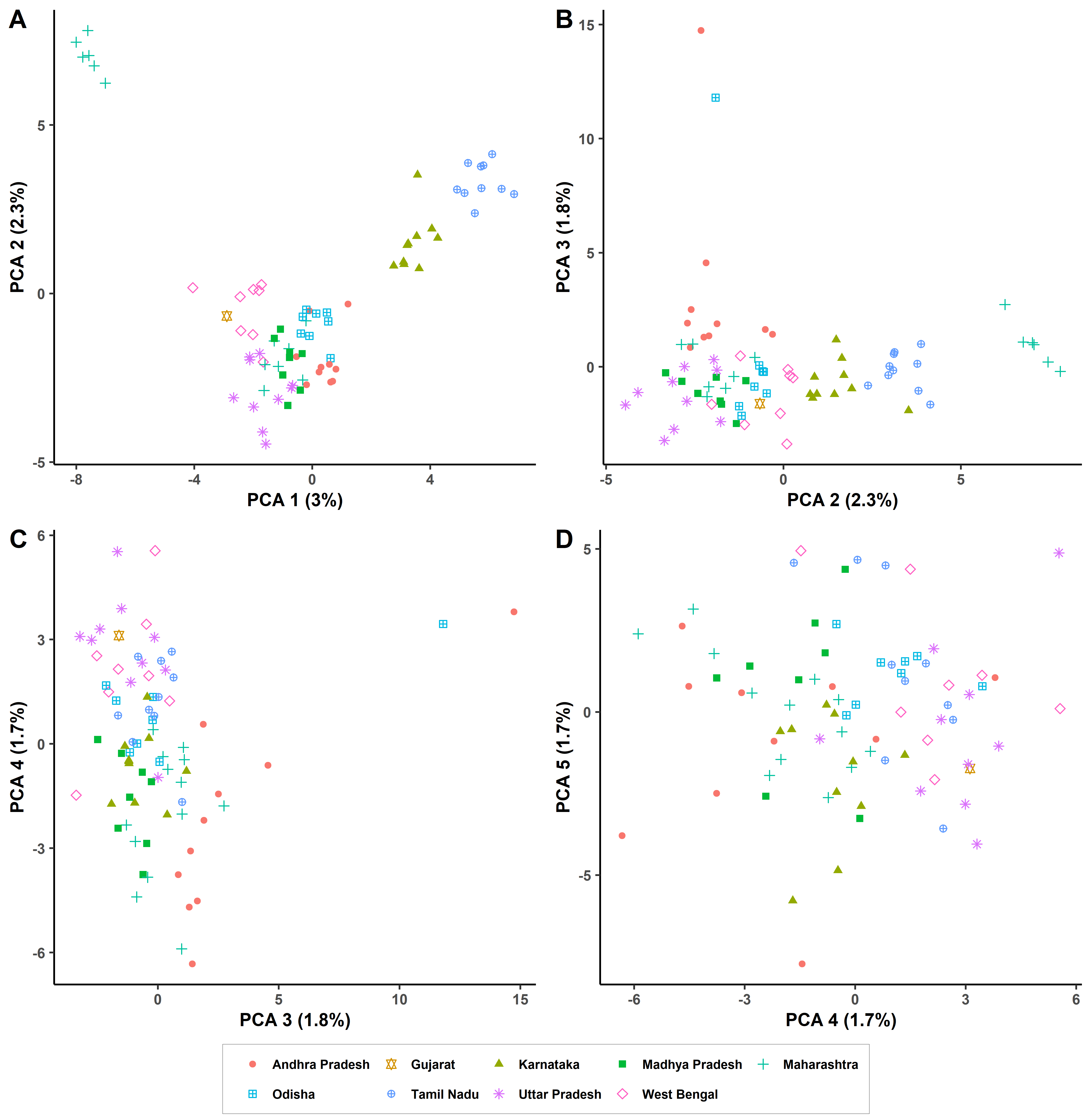


Figure S10.22 PCA plots of the DART IND dataset. A) PCA1 vs 2, B) PCA2 vs 3, C) PCA3 vs 4, and D) PCA4 vs 5. Samples are labelled based on location sampled. Note that six samples from Maharashtra cluster separately in PCA1 vs 2, corresponding to Maharashtra subpopulation A which cluster with Melbourne, Fiji, and Napier.


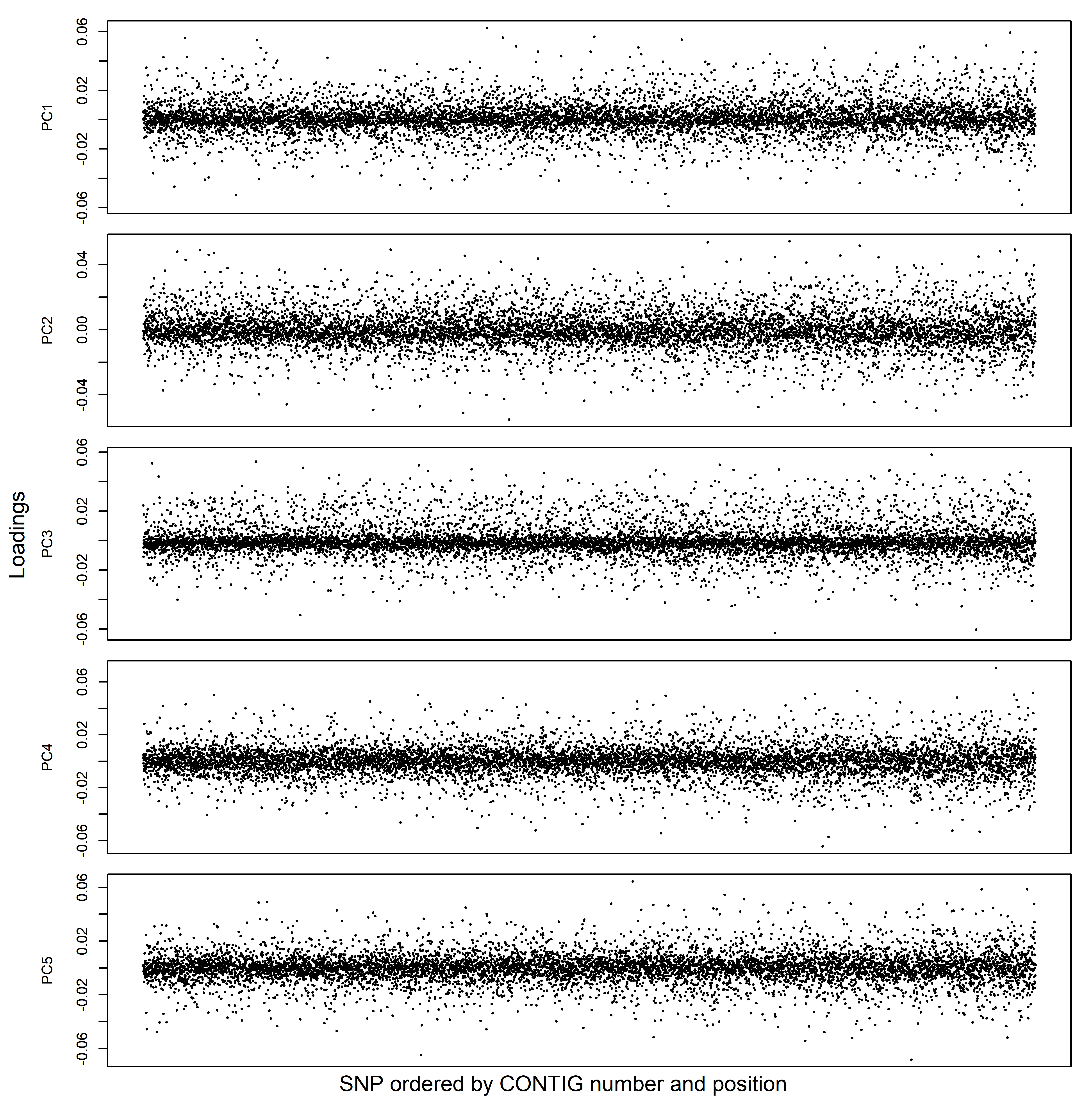


Figure S10.23 Loadings of PC1-5 from PCA on the DART IND dataset.


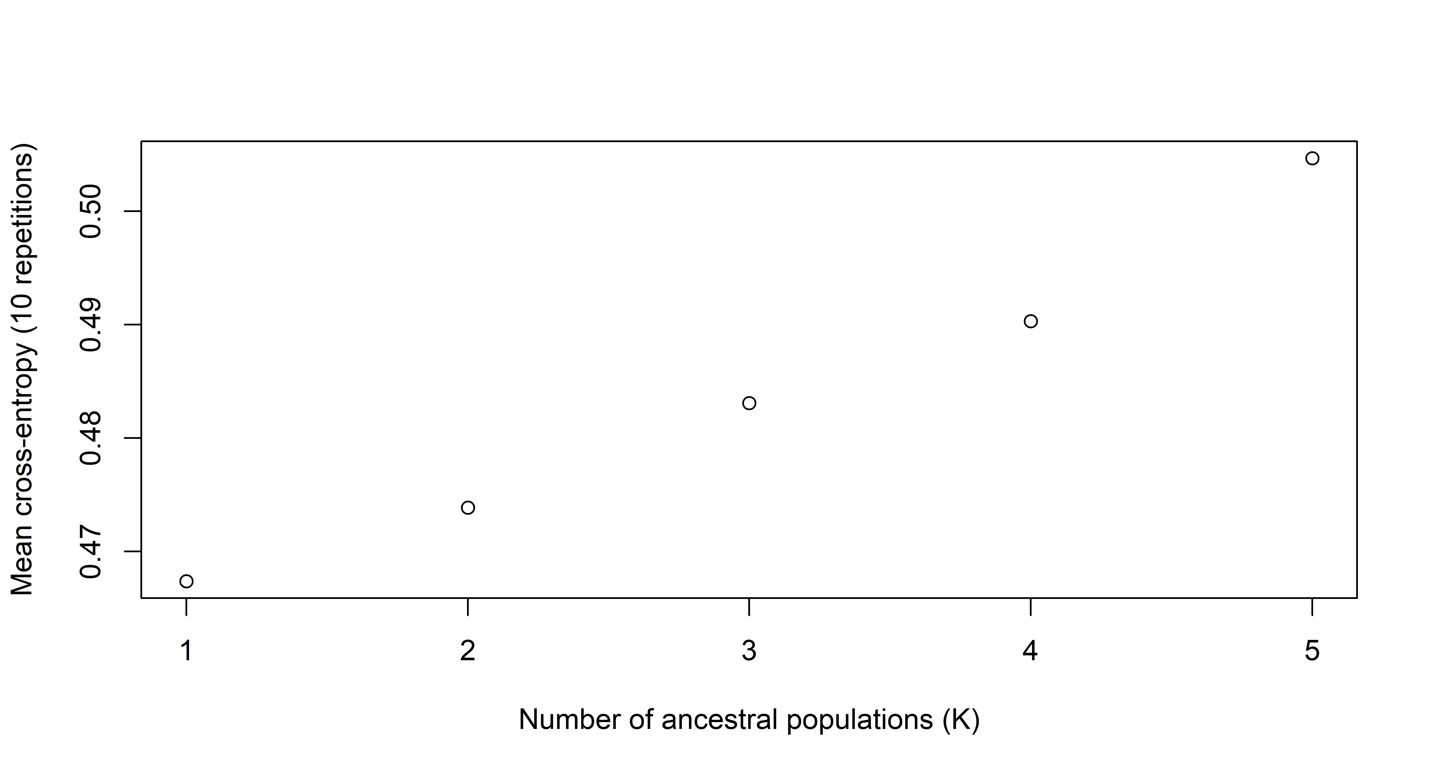


Figure S10.24 Mean cross-entropy plots of 10 repetitions at k = 1-5 of sNMF analysis on the DART IND dataset.


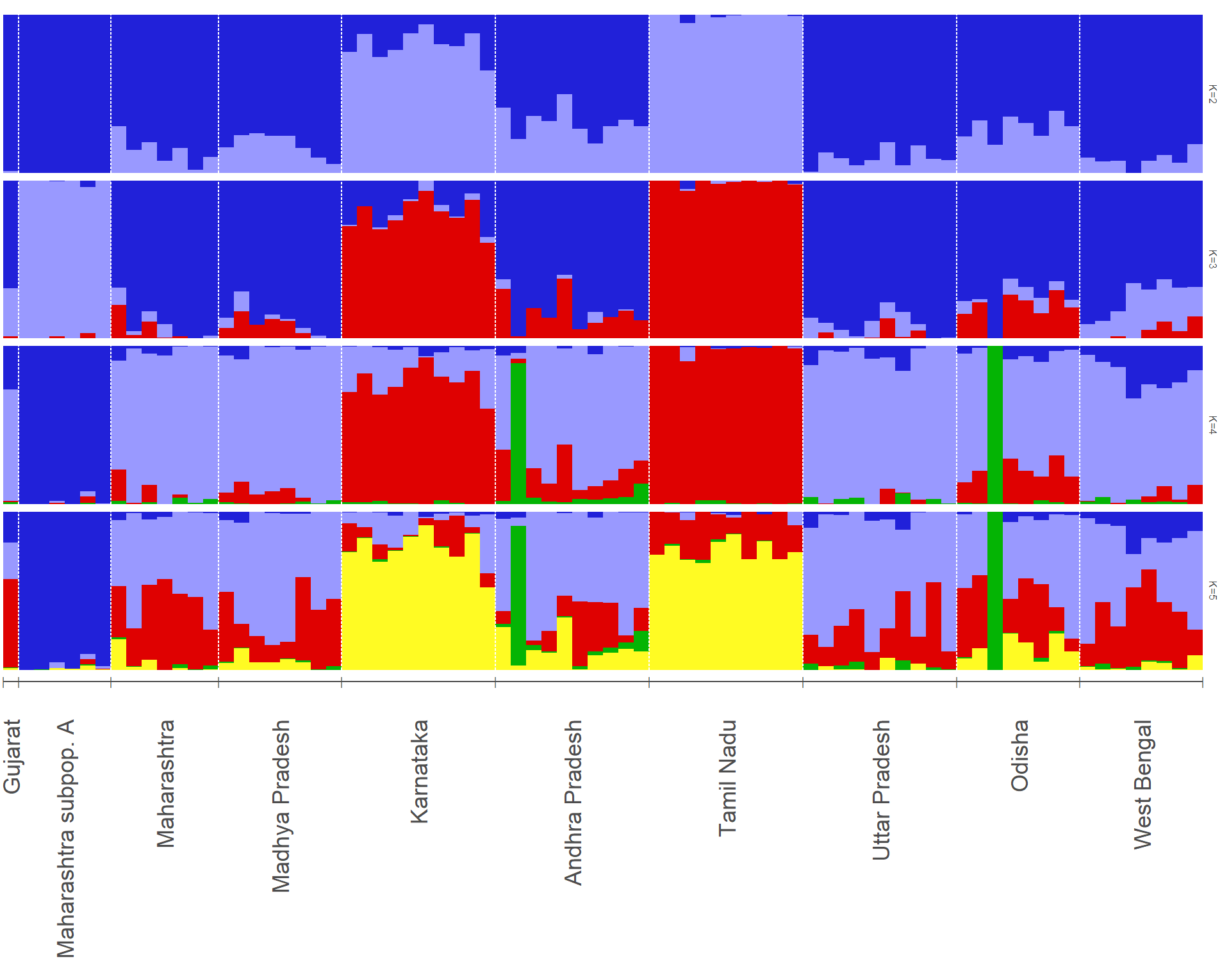


Figure S10.25 sNMF population structure plot for the DART IND dataset, k = 2-5, average of 10 repetitions. Populations are defined based on popdef1 population definition.

## DART ALL dataset

As with the BCFtools and STACKS dataset, PCA was performed on the subsampled dataset with no more than 20 individuals per introduced populations. sNMF was performed on the full dataset.


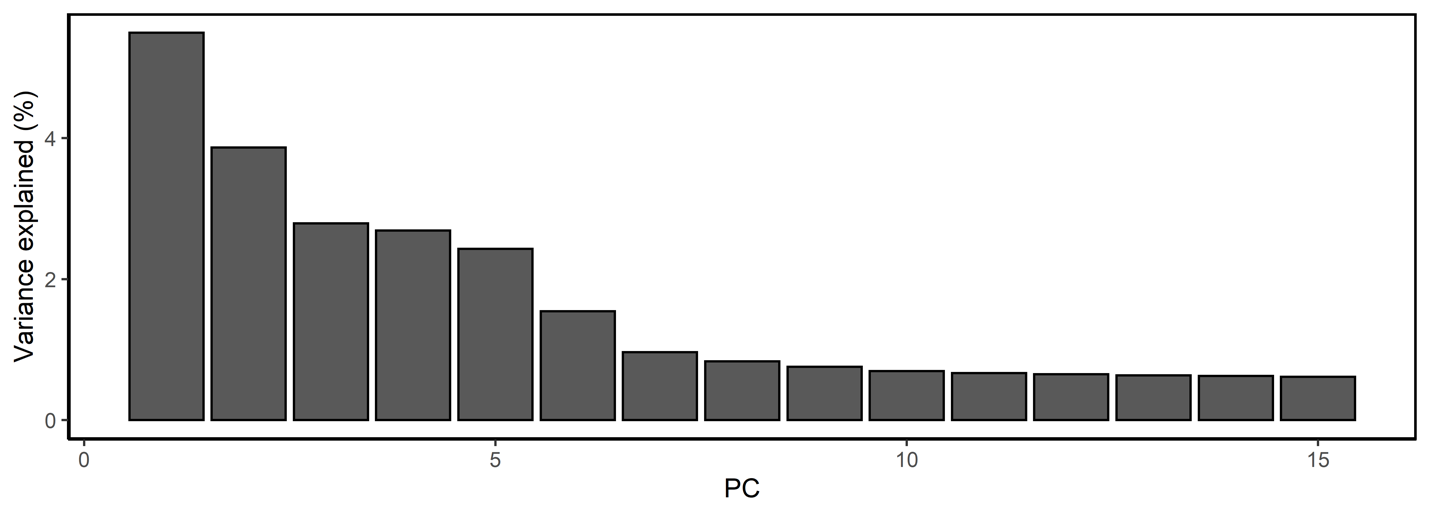


Figure S10.26 Scree plot of variance explained of the first 15 principal components from the PCA on the DART ALL dataset (subset to n ≤ 20 for introduced populations, as defined by popdef2).


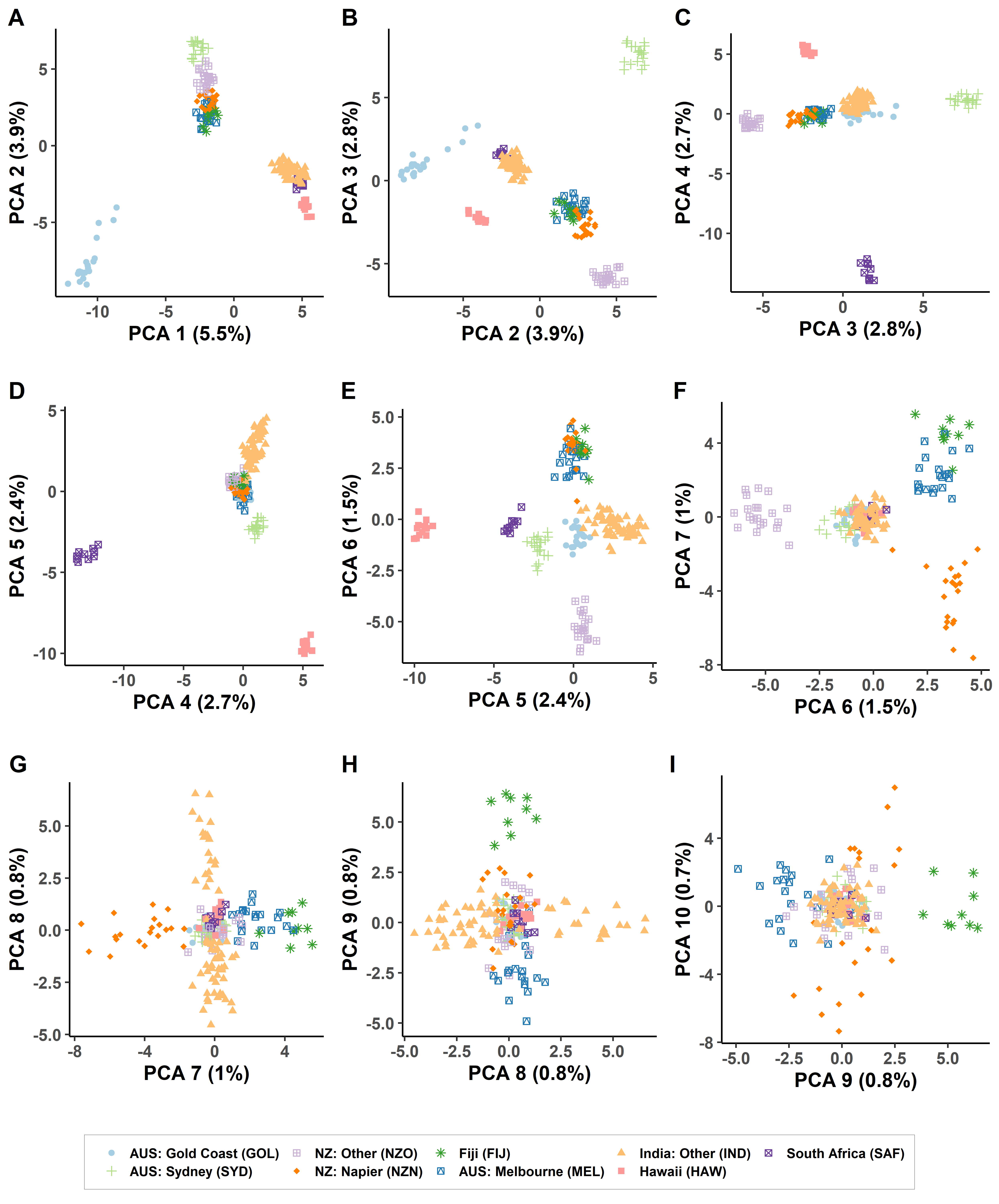


Figure S10.27 PCA plots of the ALL dataset. PCA A) 1 vs 2, B) 2 vs 3, C) 3 vs 4, D) 4 vs 5, E) 5 vs 6, F) 6 vs 7, G) 7 vs 8, H) 8 vs 9, and I) 9 vs 10. Samples are labelled based on popdef2, same as in Figure 5A in the main text.


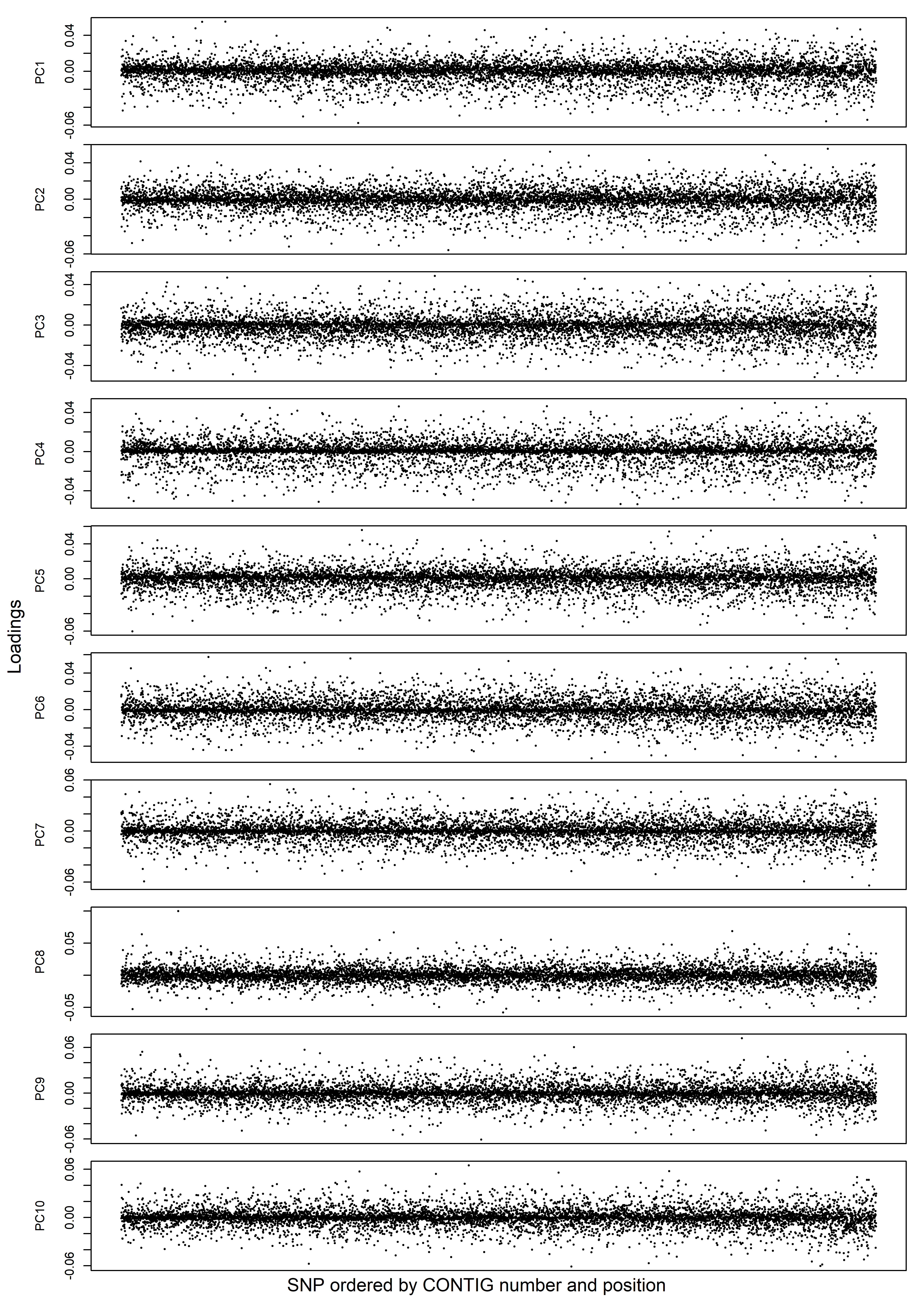


Figure S10.28 Loadings of PC1-10 from the PCA on the DART ALL dataset.


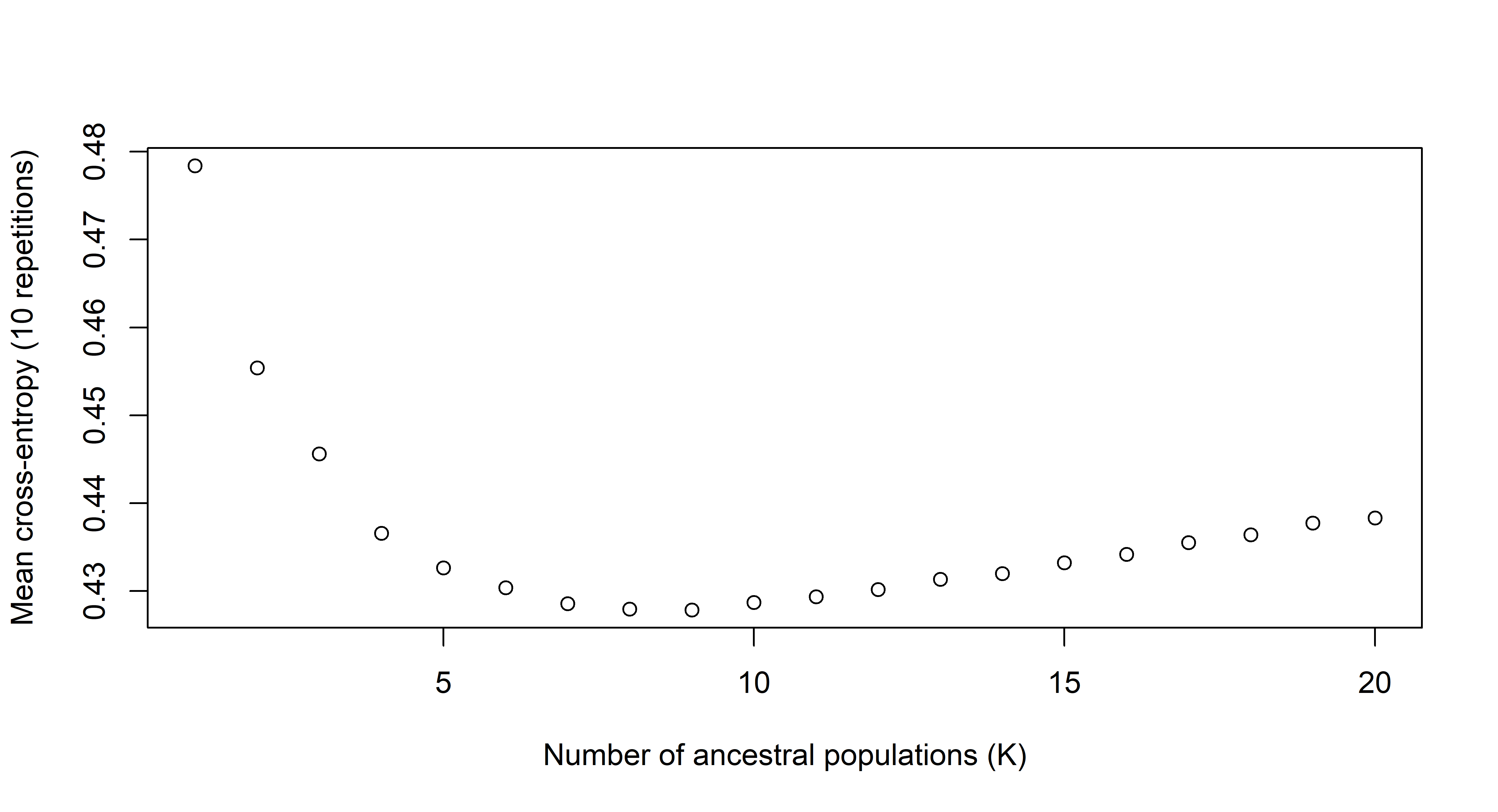


Figure S10.29 Mean cross-entropy plots of 10 repetitions at k = 1-20 of sNMF analysis on the DART ALL dataset.


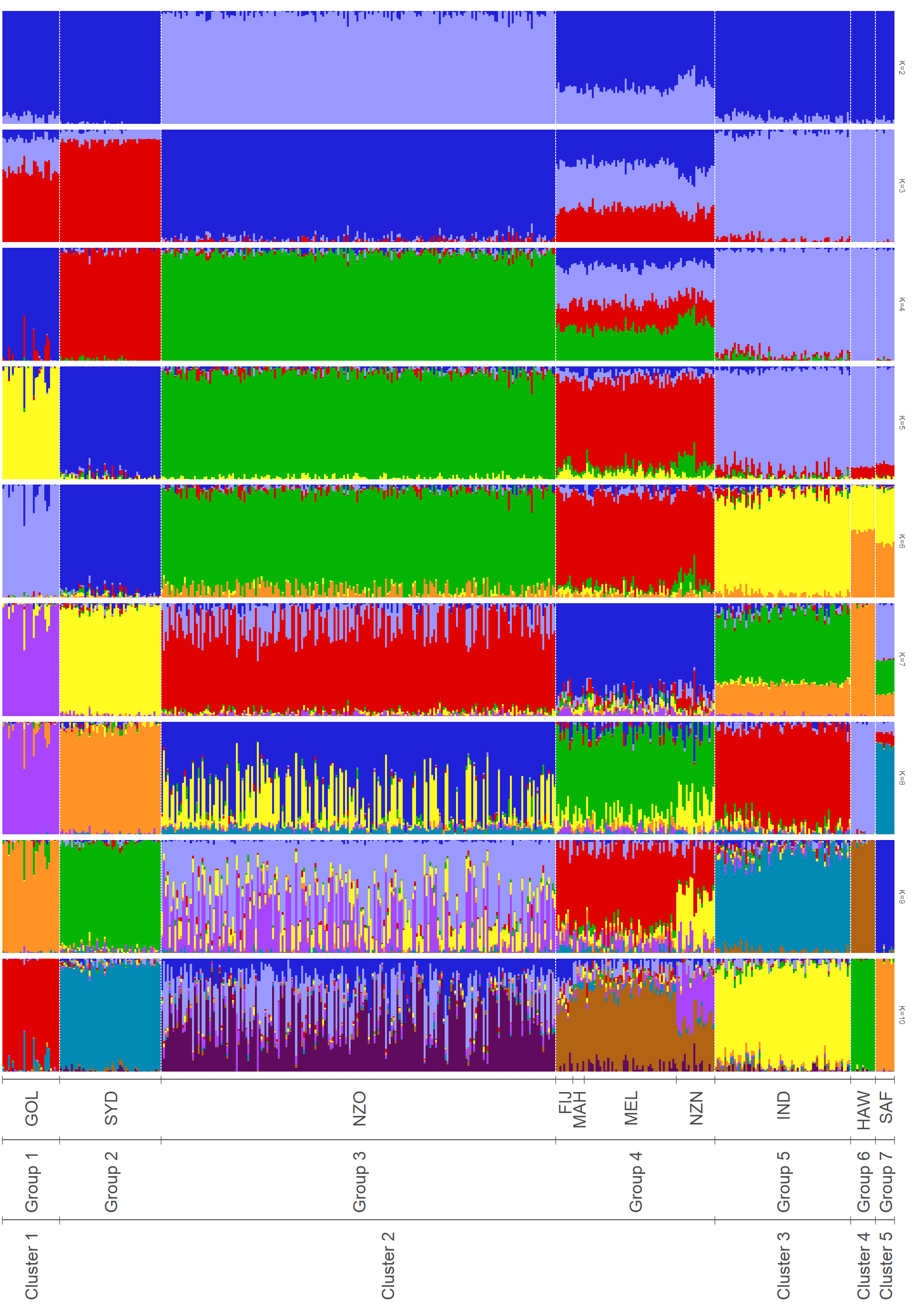


Figure S10.30 sNMF population structure plot for the DART ALL dataset. The labels correspond to labels in Figure 5 of the main text.
